# Development of a Culturally Adapted Smartphone App (IndigeQuit) Designed to Help American Indian and Alaska Native People Quit Commercial Cigarettes: User-Centered Mixed Methods Study

**DOI:** 10.2196/88768

**Published:** 2026-03-24

**Authors:** Jonathan B Bricker, Margarita Santiago-Torres, Brianna M Sullivan, Kristin E Mull, Hershel W Clark, Camille A Fogel, Soo Bin Hwang, Alison R Keith, Chase Kornacki, Trivia Afraid of Lightning-Craddock, Sydney A Martinez, Dean S Seneca, Crystal M Stanford, Christeine Terry, Sierra L Wilcox, Lonnie Nelson, Patricia Nez Henderson

**Affiliations:** 1 Fred Hutchinson Cancer Center Seattle, WA United States; 2 Department of Psychiatry and Behavioral Sciences University of Washington Seattle, WA United States; 3 IndigeQuit Community Advisory Board Cancer Consortium Seattle, WA United States; 4 Navajo Nation Window Rock, AZ United States; 5 Descendant of the Squaxin Island Tribe Shelton, WA United States; 6 Mniconjou Mato Tipila, WY United States; 7 Lakota Mato Tipila, WY United States; 8 Cherokee Nation Tahlequah, OK United States; 9 University of Oklahoma Oklahoma City, OK United States; 10 Seneca Nation Salamanca, NY United States; 11 Seneca Scientific Solutions+ Cattaraugus, NY United States; 12 University of Rochester Medical Center Rochester, NY United States; 13 University at Buffalo, State University of New York Buffalo, NY United States; 14 Chiricahua Apache Santa Clara, NM United States; 15 Black Hills Center for American Indian Health Rapid City, SD United States

**Keywords:** American Indian, Alaska Native, ceremonial tobacco, commercial cigarettes, cultural adaptation, iCanQuit, Indigenous, IndigeQuit, Native, smartphone apps, smoking cessation, tailoring, Tribal communities

## Abstract

**Background:**

Due to the colonization of tobacco plants by European settlers and the subsequent intensive marketing of commercial tobacco products to American Indian and Alaska Native (AI/AN) communities in the United States, commercial cigarette smoking accounts for half of all deaths among AI/AN people. Limited awareness, access to treatment, and the absence of culturally relevant, effective smoking cessation interventions contribute to these high death rates.

**Objective:**

This study aims to culturally adapt iCanQuit, a smartphone smoking cessation app proven efficacious for the general population, for AI/AN people.

**Methods:**

A user-centered and community-based participatory research (CBPR) mixed methods approach was applied to culturally adapt iCanQuit for AI/AN people in collaboration with a community advisory board (CAB) of AI/AN individuals using a 3-step process. Step 1 identified ways to culturally adapt the iCanQuit for AI/AN people through 1-on-1 qualitative interviews with 8 prior iCanQuit AI/AN participants. Step 2 involved developing prototypes of cultural refinements identified in step 1 through regular biweekly meetings of the CAB, research, and app development teams. The prototypes were then evaluated with a separate group of 4 prior iCanQuit AI/AN participants through 1-on-1 qualitative interviews. Step 3 involved beta testing the app through a 6-day diary study followed by 1-on-1 qualitative interviews with a nationally recruited group of 7 AI/AN adults who smoke commercial cigarettes. The development work associated with step 3 was further informed by the CAB and the research and app development teams.

**Results:**

Key findings identified 5 cultural refinements that informed subsequent app development and testing: (1) stories featuring AI/AN adults and elders emphasizing culture, spirituality, family, and community; (2) honoring the Earth as a motivator for cessation; (3) a guide character representative of AI/AN people; (4) clear distinction between ceremonial and commercial tobacco use; and (5) use of earth tones in visual design. In Step 3, all 7 (100%) diary study participants rated the beta version of the app as excellent or good/meets expectations (5/7, 71%, and 2/7, 29%, respectively) and that it felt made for them. They suggested 6 modifications which were incorporated into the final version of the app: (1) include vaping frequently asked questions, (2) feature motivation icons more prominently, (3) increase notification frequency, (4) track today’s cigarettes rather than yesterday’s, (5) allow users to update how much they spend per pack of cigarettes; and (6) rename the medications tool to reflect the inclusion of AI/AN traditional healing modalities.

**Conclusions:**

A user-centered and CBPR development process yielded IndigeQuit—one of the first known apps developed specifically to help AI/AN adults quit commercial cigarette smoking.

**Trial Registration:**

ClinicalTrials.gov NCT06145763; https://clinicaltrials.gov/ct2/show/NCT06145763

## Introduction

### Background

Due to the appropriation of tobacco plants by European settlers and the subsequent intensive marketing of chemically engineered commercial tobacco products by the tobacco industry to American Indian and Alaska Native (AI/AN) populations in the United States, AI/AN communities have a commercial cigarette smoking rate 3 times higher than that of the general population (36.5% vs 11.5%) [1-12]. For instance, research analyses of secret tobacco industry documents by Lempert and Glantz et al [9] revealed specific promotional strategies used to exploit Native imagery and target these populations, thereby contributing to the higher-than-average rates of commercial cigarette smoking among AI/AN people compared with any other race in the United States. Consequently, AI/AN communities have 6 times higher rates of smoking-related cancers, as well as smoking-related respiratory and heart disease [13-15], and are only half as likely to quit smoking as other races and ethnicities [16,17]. The result is that commercial cigarette smoking now accounts for half of all deaths among AI/AN communities nationwide [13,18,19].

There are complex historical, social, and cultural factors that have contributed to these longstanding inequities in rates of commercial smoking and related deaths. For instance, due to federal policies that forbade cultural ceremonial practices, the use of ceremonial tobacco has become infrequent and has largely been replaced by commercial cigarette smoking [1-8]. Decades-long predatory marketing by the tobacco industry has further exacerbated smoking rate inequities among AI/AN communities [8-10]. Examples include the use of advertising with Native imagery in tobacco products and the sponsorship of cultural events targeting marginalized populations, including racial and ethnic minorities, people of low socioeconomic status, and youth. Additionally, historical trauma, racism, and discrimination have fostered mistrust of and fear of the health care system among AI/AN communities, resulting in limited access to efficacious smoking cessation treatment [20-24]. The inaccessibility of treatment is further compounded by lack of insurance coverage and lack of systems for universal assessment, referral, and integration of services into routine care for these communities [25-29].

One method for AI/AN communities to access evidence-based smoking cessation treatments is via smartphone-based smoking cessation apps [30,31]. Smartphone apps can deliver all the smoking cessation content now found on websites and SMS text messaging interventions—other common digital intervention delivery modalities [30-33]. Apps are an important technological advance over these modalities because they do not always require internet access and have more engaging features than websites and SMS text messaging interventions [34-42]. These apps can reduce smoking inequities because they do not require interaction with the health care system, can be freely accessed through an app store, and are available anytime at the convenience of the user. The reach of smoking cessation apps for AI/AN adults who smoke is climbing rapidly, greatly aided by growing smartphone ownership in Native communities [43-45].

To help address the reach of digital smoking cessation treatments for all adults who smoke in the United States, our team developed iCanQuit, a smoking cessation smartphone app based on the principles of acceptance and commitment therapy (ACT) [46]. ACT for smoking cessation teaches skills for allowing cravings to pass without smoking and motivates individuals to quit by appealing to deeply held personal values, such as family, community, cultural traditions, and health [47-51]. iCanQuit is the only app proven efficacious for smoking cessation in a randomized controlled trial (RCT) with long-term 12-month follow-up among 2415 adults recruited from all 50 US states. Compared with participants using the National Cancer Institute (NCI) QuitGuide app based on the US Clinical Practice Guidelines (USCPG) for smoking cessation, iCanQuit participants had 1.5 times higher odds of quitting smoking at 12 months (28% vs 21% quit rate; odds ratio [OR] 1.49, 95% CI 1.22-1.83; *P*<.001) [46].

A subsequent secondary analysis of the iCanQuit RCT among the subsample of AI/AN adults (N=165) recruited from 32 US states (25% residing on Tribal land) showed very encouraging results. AI/AN participants assigned to iCanQuit had 2.6 times higher odds of quitting smoking compared with AI/AN participants assigned to QuitGuide at 6 months (25% vs 11%; OR 2.62, 95% CI 1.06-6.45; *P*=.04) and correspondingly higher odds of quitting smoking at 12 months (30% vs 18%; OR 1.96, 95% CI 0.90-4.26; *P*=.09) [52]. Treatment engagement (number of sessions, 32.4 vs 8.5; *P*<.001) and satisfaction with the assigned app (92% vs 65%; *P*<.001) were also significantly higher among AI/AN participants using iCanQuit compared with those using QuitGuide [52]. Changes in the ACT-based process of accepting bodily sensations, emotions, and thoughts that cue smoking significantly mediated the effect of iCanQuit on cessation outcomes compared with QuitGuide (all *P*<.01).

### Objectives

Although encouraging, iCanQuit was not developed specifically to help AI/AN adults quit commercial cigarette smoking. To better support AI/AN adults quitting commercial cigarette smoking, we implemented a user-centered and community-based participatory research (CBPR) development process to culturally adapt iCanQuit and make the app-based smoking cessation intervention more relevant, inclusive, and meaningful for AI/AN communities. Here, we report the details of the 3-step iterative development process grounded in CBPR principles, AI/AN cultures, and an ACT-based behavioral approach for smoking cessation that resulted in the IndigeQuit app—one of the first known apps developed specifically for AI/AN adults quitting commercial cigarette smoking. The IndigeQuit app is currently being tested against QuitGuide in a full-scale randomized trial among 776 nationally recruited AI/AN adults who smoke commercial cigarettes and want to quit.

## Methods

### IndigeQuit Trial Overview

The cultural adaptation of iCanQuit for AI/AN communities preceded the launch of a large-scale RCT currently testing the efficacy of the culturally adapted smartphone app for commercial cigarette smoking cessation, named IndigeQuit, against the NCI-developed USCPG-based smartphone app (QuitGuide).

### Ethical Considerations

Study procedures were approved by the Institutional Review Board at Fred Hutchinson Cancer Center (protocol number FHIRB0020279/RG1123796; ClinicalTrials.gov: NCT06145763). Written and verbal informed consent was obtained from participants. To protect participants’ privacy and confidentiality, all collected data were deidentified and stored in a password-protected format.

### Community Advisory Board

#### Formation

The IndigeQuit RCT is being implemented by a multidisciplinary community advisory board (CAB) and research team consisting of AI/AN and non-AI/AN investigators. We partnered with AI/AN community members to culturally adapt the intervention through a collaborative and iterative process following CBPR principles [53]. To form the CAB, AI/AN investigators extended invitations to AI/AN community members within their networks who had an interest in improving the health of AI/AN people by reducing commercial tobacco use in their communities. Prior AI/AN participants in the iCanQuit parent RCT who had agreed to be contacted for future research were also recruited for CAB participation by study staff. Others were identified by active CAB members.

Ten individuals accepted the invitation via email. Study staff then followed up with an official invitation letter and a CAB agreement outlining the study aims and expectations, as well as the CAB members’ roles and responsibilities. Each CAB member was asked to read and sign the CAB agreement before joining, which included a 1-year minimum commitment. The first meeting was conducted in June 2024, and the CAB has met biweekly to the present day. In early meetings, facilitators highlighted the value of input from the community and introduced details about the parent RCT, including the background and significance of the work ahead. CAB members are compensated US $100/hour for their time.

#### CAB Members

CAB members hold a wide variety of roles and perspectives, including current and former commercial cigarette users, prior iCanQuit RCT participants, family members of people who smoke, tobacco treatment specialists, physicians, public health professionals, and leaders of health organizations that serve the AI/AN community, as well as national AI/AN leaders in the campaign for cessation of commercial tobacco within Tribal Nations. CAB members represent diverse Tribal affiliations across North America, including Cherokee, Lakota, Navajo, Coast Salish, and Seneca, as well as geographic residences in Arizona, California, Kansas, New Mexico, New York, Oklahoma, South Dakota, and Washington.

### User-Centered and CBPR Development Process

#### Overview

The mixed methods research for culturally adapting iCanQuit for AI/AN communities followed a 3-step development process consistent with user-centered design and CBPR principles. The steps were further informed by the CAB, research team, and app development team through regular biweekly meetings between June 2024 and July 2025. Each step in the iterative development process is described below.

#### Step 1: Needed Changes

##### Overview

Step 1 aimed to identify changes needed to make the iCanQuit app more culturally relevant, acceptable, and inclusive for AI/AN people through 1-on-1 semistructured qualitative interviews with prior AI/AN participants in the iCanQuit parent RCT.

##### Participants

iCanQuit RCT participants who (1) self-identified as AI/AN; (2) received the iCanQuit app; (3) agreed to be recontacted in the future; and (4) were willing and able to participate in a 60-minute video call were invited to participate. The full inclusion criteria for the iCanQuit RCT have been previously published [46,52]. Briefly, these included smoking 5 or more commercial cigarettes per day in the past year and wanting to quit.

##### Recruitment

The recruitment process started with an email invitation to randomly selected prior iCanQuit RCT AI/AN participants who met the eligibility criteria. If a participant expressed interest, study staff asked about their availability, access to a device for a video call, and willingness to be interviewed and recorded. Of the participants contacted, 19 individuals expressed interest in participating, 9 were available and scheduled for an interview, and 8 completed the interviews. Thematic saturation was not used as a stopping criterion. Rather, the number of interviews was dependent on prior iCanQuit AI/AN participants’ availability, logistical constraints (eg, time, budget, personnel), and our limited goal of producing actionable cultural refinements to the iCanQuit app.

##### Interviews

The interview guide was developed collaboratively by AI/AN and non-AI/AN investigators with expertise in behavioral interventions, digital health, and commercial tobacco cessation. The semistructured interview guide included open-ended questions that began with participants’ overall experiences with the iCanQuit app, followed by more specific questions about how each section of the iCanQuit app–based intervention could be made more culturally relevant, acceptable, and inclusive of AI/AN people (Multimedia Appendix 1). All interviews were conducted via a Health Insurance Portability and Accountability Act (HIPAA)–compliant video call, and verbal consent was obtained from each participant before the interview. Data were collected in September 2022. Participants were compensated US $100 for their time.

#### Step 2: Prototype Iterative Development

##### Overview

Building on the qualitative findings from step 1, we engaged in an iterative process of adaptation and development in collaboration with the study CAB from June 2024 to August 2025. We co-developed prototype versions of the refinements, which were then evaluated by a separate group of 4 prior iCanQuit RCT AI/AN participants through 1-on-1 qualitative interviews conducted in November 2024. The results of these interviews were shared with the CAB, research team, and app development team, and suggested edits were incorporated. The CAB continued to provide input through biweekly meetings, generating additional refinements to strengthen culturally appropriate contextual fit. Results from the 6-day diary study and qualitative interviews conducted in June 2025 and described in step 3 were also presented to the CAB, which provided final feedback. This 14-month process culminated in August 2025 with the CAB’s review and final approval of the app.

##### CAB Meetings

Three investigators (2 of whom self-identify as AI/AN and 1 non-AI/AN) jointly led the biweekly meetings with the CAB (n=10), research team (n=10), and app development team (n=3), which served as a collaborative forum to review app features and provide feedback. Meetings were held virtually via a HIPAA-compliant, passcode-enabled Zoom room (Zoom Communications). Each meeting lasted approximately 1.5 hours. Throughout these meetings, the app development team used CAB feedback to iterate on new app designs and features, which were subsequently brought back to the group for additional review. In parallel, app content was iteratively reviewed, and additional feedback was solicited to ensure the tone, language, and messaging resonated with AI/AN users. Over the course of 25 virtual meetings and a 2-day in-person retreat in February 2025, 8 design iterations of the app were generated, yielding the beta version of the app, which was subsequently tested in the 6-day diary study described in step 3.

##### Interviews

Midway through the prototype development with the CAB, qualitative interviews were conducted with a separate group of 4 prior iCanQuit RCT AI/AN participants. The purpose of these interviews was to collect feedback on the new prototype designs for the app (ie, home screen, avatar “guide” persona, motivational values, and images) and to identify any further refinements needed for clarity, relevance, relatability, and usefulness. The eligibility criteria, recruitment, and enrollment process were identical to step 1. Of the 87 participants contacted, 9 individuals expressed interest in participating, 8 were eligible and scheduled for an interview, and 4 completed the interviews. For this step, a more structured interview guide was implemented that included open-ended questions focusing on the evaluation of the prototypes developed in step 2 (Multimedia Appendix 2). Data were collected in November 2024. Participants were compensated US $100 for their time.

#### Step 3: Beta Testing

##### Overview

Step 3 involved a 6-day diary study followed by 1-on-1, semistructured 45-minute qualitative interviews. The purpose of the diary study was to test the beta version of the app with a nationally recruited group of AI/AN adults who smoke commercial cigarettes and had no prior experience with the iCanQuit app. Throughout the diary study, participants provided daily ratings for each app feature via online surveys assessing clarity, relevance, relatability, and usefulness. At the end of the diary study, 1-on-1 qualitative interviews explored participants’ experiences with the app in greater depth.

##### Participants

Eligibility criteria were as follows: (1) self-identify as AI/AN, either alone or in combination with other races; (2) age 18 years or older; (3) have smoked commercial cigarettes in the past 30 days; (4) have daily access to their own Android or iPhone; (5) be able to download a smartphone app; (6) be willing and able to read English; and (7) have no other household or family member participating.

##### Recruitment and Enrollment

Guided by the CAB, social media ads featuring images of IndigeQuit AI/AN testimonial characters were designed to refer AI/AN individuals to the screening survey. Interests associated with AI/AN communities were selected for ad targeting. These included Native languages; hobbies such as beading, woodworking, leatherworking, and rodeo; and areas of study such as Native American studies and herbalism.

Those who were eligible were contacted for phone screening and then scheduled for onboarding interviews, during which participants verbally provided informed consent and received instructions for completing diary entries and downloading the beta version of the culturally adapted app. Of the 31 eligible participants identified through online screening, 13 completed phone screening, and 10 remained eligible. Eight completed onboarding interviews, and 7 completed the diary study. Data were collected in June 2025. Participants were compensated US $30 for the onboarding interview, US $15 for each completed daily survey, and US $50 for completing the exit interview.

##### Six-Day Diary Study

For 6 consecutive days, participants were asked to complete an online survey that combined closed- and open-ended prompts to evaluate user experience ratings, perceived usefulness, and engagement with the app. Daily surveys focused on specific app features. Across the 6 days, the surveys collected data through (1) usage checks (eg, “Did you set a quit date?”); (2) Likert-type ratings on a 5-point scale to measure perceived experience and app feature value (eg, rating the value of audio exercises from “1=not very valuable” to “5=very valuable”); and (3) ranking tasks (eg, “Please rank the following types of notifications from 1=most liked to 5=least liked”). Open-ended items (eg, “Please tell us why you chose that rating. We’d like you to be as detailed as possible”) helped supplement the insights provided during the 1-on-1 interviews.

##### Interviews

Both the diary surveys and interview guide were collaboratively developed by the CAB and research team. The semistructured interview guide included open-ended questions about the participant’s overall experience with the app, followed by more specific questions on how each section of the app was perceived (Multimedia Appendix 3).

### Coding and Analysis

Audio recordings of the 1-on-1 interviews in steps 1-3 were professionally transcribed and inductively coded according to a thematic analysis paradigm [54]. Participants’ write-in responses to daily diary prompts in step 3 were treated in a similar manner. Inductive thematic analysis was selected to aid in hypothesis generation on actionable cultural refinements to the app. It also allowed us to identify and characterize patterns across participants’ recommendations and CAB members’ input to make the iCanQuit app more culturally relevant, acceptable, and inclusive of AI/AN people.

Authors CAF and ARK conducted the analysis. After familiarizing themselves with the data, they independently applied line-by-line open coding to identify preliminary themes that described participants’ experiences with the app and their recommendations for how it could be improved. Both are long-term members of the research group and have contributed to various smoking cessation and weight loss clinical trials, including qualitative fidelity coding of hundreds of telephone-based counseling sessions. ARK has coordinated multiple smoking cessation trials. CAF holds a master’s degree in experimental psychology and has led multiple qualitative research projects. The 2 coders then met and collaboratively developed, reviewed, and defined a final set of themes and recommendations. For each step, results were presented to the CAB to ensure credibility of the findings through member checking and to stimulate additional conversation about how participant feedback could be incorporated into the app.

## Results

### Step 1: Needed Changes

#### Participant Characteristics

The 8 iCanQuit parent RCT interview participants were from 7 US states (Florida, Kansas, North Carolina, Nevada, Oklahoma, Texas, and Washington). On average, participants were 40.5 (13.7) years old, including 5 women and 3 men. There were 5 Tribal affiliations represented: Cherokee, Cree, Creek, Lakota Sioux, and Tsimshian. According to participants’ ZIP codes linked to federally recognized Tribal lands, 1 of the 8 participants was residing on Tribal land. Regarding smoking status at the time of the interviews, 5 had quit commercial cigarette smoking, 2 were in the process of quitting, and 1 was currently smoking commercial cigarettes.

#### Summary of Results

##### Content Analysis

Content analysis revealed the number of times concepts were mentioned during the interviews (from most to least mentioned): (1) stories from elders; (2) importance of nature; (3) more detailed information about commercial smoking cessation medications; (4) voice recordings or testimonials from Natives; (5) differences between traditional and commercial tobacco use and the lack of education regarding commercial tobacco use; (6) inclusion of appropriate and relevant Tribal symbols; (7) importance of family and community; and (8) changes to the color palette and the addition of imagery of AI/AN people clothed in Native fashion or styles. Four additional interpretive themes emerged from the analysis.

##### Theme 1: Nature, Elders, and Storytelling Are Important

Although participants came from different Tribes with varying traditions, most talked about connecting with Mother Earth, the importance of elders, and the significance of sharing stories and traditions. Across Tribes, storytelling is a traditional and highly respected means of passing down wisdom from generation to generation. Many stories include themes about being connected to one’s community and the earth. Some participants said these cultural stories may be a way to share messages about commercial smoking. One participant explained that Indigenous people have a particular cadence and way of speaking, suggesting that including the voices of people telling their stories may create additional opportunities to build trust and connectedness with the app.

##### Theme 2: Medications Are Commonly Misunderstood

A lack of trust in commercial smoking cessation medications was a common theme across individuals interviewed. Participants reported a lack of trust, a lack of education, and limited access to health care as major barriers to using medications to quit commercial smoking. Several participants also stated that trust could be built by including direct statements about medication. Instead of just listing medications, the app could state what the medications do and how they work. One participant shared: “I was chewing gum and smoking a cigarette thinking it was gum. I was like no, it doesn’t work like that. This is medication that’s actually trying to help you.”

##### Theme 3: Culture and Connectedness

Participants commonly mentioned Mother Earth, elders, family, and community, and described Mother Earth and family as positive ways to connect with AI/AN culture. In Indigenous cultures, Mother Earth refers to the Earth as a nurturing and life-giving entity. Adding stories about how quitting commercial smoking benefits the environment and community may be a way to incorporate AI/AN culture. Example quote: “Honoring Mother Earth by not ruining the ground with filters that are filled with chemicals...The plastic and cigarette butts will be here long after we are.”

##### Theme 4: Distinction Between Traditional and Commercial Tobacco

Several participants stated that their Tribes did not actively educate members about the difference between traditional ceremonial tobacco use and commercial tobacco use, with one noting that traditional tobacco is no longer used in their Tribe. One participant explained how the ceremonial context makes a key distinction: “If I go to a ceremonial situation where there’s going to be a tobacco pipe used,...if it’s part of the tradition, I can participate in that and then walk away from it because I’m doing it as a religious observation. It’s just like if you go to church and take communion, you’re not going to immediately go out and chug a bottle of wine.”

### Step 2: Prototype Development

#### Cultural Refinements to the iCanQuit App

The themes identified in step 1 informed 5 specific cultural refinements to the iCanQuit app that served as the starting point for discussion and design iterations in collaboration with the CAB in step 2: (1) modify the app’s stories to feature AI/AN adults and elders who quit commercial smoking, emphasizing the values of spirituality, family, and community as motivators; (2) add honoring the Earth as a motivator to quit smoking; (3) make the appearance of the app’s “guide” character more representative of AI/AN people; (4) add information distinguishing ceremonial and commercial tobacco; and (5) use earth tones in the app’s colors. The suggested cultural refinements were incorporated through changes to the app’s visual design and content.

#### Iterative Cultural Adaptation of Visual Design

**Figure 1:**
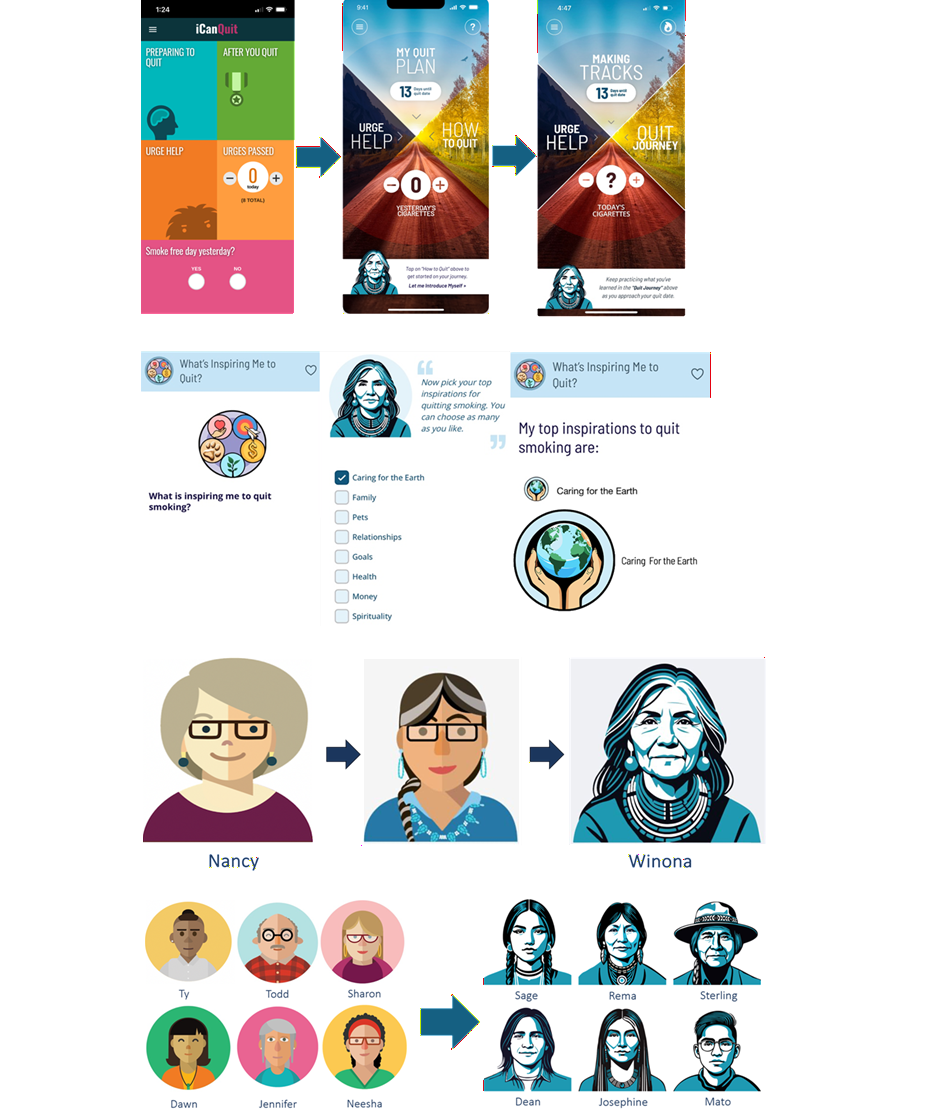
Iterative visual design updates used to create step 2 prototypes. (A) (Home screen) This sequence illustrates the evolution of the app’s home screen across design iterations. The left image shows the original iCanQuit home screen. The middle image reflects an early revision informed by participant and community advisory board (CAB) feedback, featuring the Medicine Wheel—a circular symbol divided into 4 quadrants that represents wellness for many Native people—as the central image and navigation menu. The right image shows the final revised design. (B) (What’s Inspiring Me to Quit?) An interactive feature in which users select 1 or more personal motivations for quitting smoking from a set of illustrated, values-based icons. The iconography was updated to include Native symbols. The "Caring for the Earth" icon, shown here, emphasizes connections between smoking cessation and honoring the earth as part of supporting balance, health, and responsibility to future generations. Selected inspirations are displayed back to users to reinforce motivation during their quit journey. (C) (Guide avatar) The original iCanQuit guide avatar, "Nancy" (left), was redesigned as "Winona" (right), a Native-named elder intended to feel more familiar to Native users and to serve as a source of wisdom and support. The sequence illustrates the evolution of Nancy across design iterations leading to the final Winona design. (D) (Additional avatars) The left image shows the original iCanQuit set of additional avatars. The right image shows the revised set developed through iterative design informed by participant and CAB feedback to reflect Native features, hairstyles, and names.

The visual design of the app was culturally adapted to include Native imagery, symbolism, and color palettes. The app home screen, icons for values-based motivations, and the appearance of the avatar characters were the most heavily revised visuals (Figure 1). The original landing home screen page in iCanQuit was updated to feature the Medicine Wheel, a circular symbol divided into 4 quadrants that signifies living in balance for Indigenous populations, and it was used as the central image and navigation menu (Figure 1A). While interpretations of the Medicine Wheel may vary across Tribes, such as the 4 Directions (East, South, West, and North) or aspects of life (spiritual, emotional, mental, and physical), it is a powerful image representing wellness for Native people [55]. Bright earth-tone colors were incorporated throughout the app, echoing colors commonly seen in ceremonial regalia.

Nature motifs were incorporated throughout to reflect user feedback that the nature theme should be represented in both the visual design and the app content. For instance, the values-based motivation iconography was updated to include Native symbols such as the Medicine Wheel to represent wellness and a mountain to represent spirituality. The “Caring for the Earth” motivation icon was designed to emphasize honoring nature through connections between smoking cessation and stewardship of the land, respect for natural elements, and the role of honoring the Earth in supporting balance, health, and responsibility to future generations. Motivational icons (eg, “Caring for the Earth”) were included within the app feature “What’s Inspiring Me to Quit?,” where users select their top inspirations for quitting smoking (Figure 1B). Regarding the app content, illustrations of an “urge monster” that had been used to indicate the strength of a craving were replaced with a water metaphor, prompting users to select the strength of their urge, ranging from a drop to a flood.

Finally, the avatars that share their quitting journeys were completely redesigned to have Native features (eg, defined nasolabial folds), hairstyles (eg, traditional braids as well as contemporary styles), accessories (eg, silver jewelry), and names (eg, Sterling or Dean, after 1 of the CAB members). Extra attention was given to redesigning “Nancy,” the original iCanQuit guide avatar, into “Winona,” a Native-named elder who would feel more familiar to Native users and be viewed as a source of wisdom and support (Figure 1C and D).

#### Mid-Way External Evaluation of Prototype Designs: Qualitative Interviews

##### Participant Characteristics

Four participants were recruited from 4 US states (Oklahoma, Tennessee, Texas, and Washington). On average, participants were 38.3 (7.8) years old, and 3 of the 4 were women. There were 5 Tribal affiliations represented: Cherokee, Chiricahua Apache, Creek, Lakota, and Zapotec. At the time of the interviews, all 4 participants reported living off-reservation, and 3 had quit commercial cigarette smoking.

##### Summary of Results

Participants provided feedback on the new prototype designs for the app home screen, lessons menu, avatars, and motivation icons. Participants found the home screen attractive and easy to navigate, and the lessons menu layout seemed intuitive. They liked the earth tones and the avatar designs. Participants viewed the guide as strong yet approachable, and 3 associated the image with their own grandmother or community elder. All participants felt the values and associated icons made sense and looked good overall, although revisions were suggested for the family and health icons. Three additional themes emerged during the interviews.

##### Theme 1: Opportunity for Community Building

Multiple participants viewed the app not only as a tool to quit commercial cigarette smoking, but also as an opportunity to connect with their community and alleviate feelings of isolation while quitting. One participant described: “My cousins were smoking. My dad’s still smoking. My aunt’s smoking. Everybody’s smoking...So that was the hardest part. It still is sometimes. Everybody else goes out and smokes and has the little break and then you’re just trapped inside.” This participant suggested the app could be strengthened by allowing users to connect: “It would be really cool to have a community to be able to interact with other users...because you build that community and you know that somebody else is suffering with you at certain points.”

##### Theme 2: Connecting to Native Culture Enhances Motivation

Participants overall provided positive feedback about aspects of the app that had been adapted to better fit Native users, and 2 emphasized that connecting to Native culture can be a motivating force in quitting commercial cigarettes. One participant suggested capturing this with an additional value icon: “...preserving yourself as a person and as a body for your culture’s sake. That could be a motivation because it’s like being extinct or endangered species.”

##### Theme 3: The Importance of Indigenous Contributions and Authenticity

Participants emphasized the importance of Indigenous people being involved in the creation of the app and the smoking-cessation intervention more generally. They also expressed concern that a lack of Indigenous voices in the app’s development could affect its credibility among potential users. One participant said, “I think just knowing that the representation that they worked with Native communities would be more appealing. Just even if it’s a fictional character, but a quote from someone within communities that we worked on this app would be something that would make it more attractive.”

The themes identified through this process of external evaluation of the prototype designs informed additional cultural refinements to the IndigeQuit app: (1) incorporating a social component into the app and its content that normalizes and alleviates feelings of shame or isolation associated with trying to quit; (2) adding a new motivation icon to capture the desire to engage in cultural preservation by caring for one’s body; and (3) clearly indicating that Native people were involved in developing and adapting the app. These suggestions were discussed with the CAB and stimulated additional content iterations described below.

### Iterative Cultural Adaptation of App Content

#### Modifications to Original iCanQuit Content

App content was also culturally adapted. The name of the app was changed from iCanQuit to IndigeQuit, and language throughout was adapted to better resemble the way Indigenous people speak and interact with one another. For example, to begin their quit journey, users now select a button labeled “Skoden,” a common abbreviation of the phrase “let’s go then” in reservation (rez) slang. Additionally, Lakota language phrases such as *Mitakuye Oyasin* (all my relations) were added to boost the familiarity and credibility of the guide avatar, Winona. Daily push notifications to increase app engagement were interspersed with inspiring quotes from Native leaders and poets that reinforce the ACT principles of acceptance, willingness, and living in line with one’s values.

Further elaboration about Winona’s backstory was added to her introduction in response to feedback that, in Native communities, it is common to provide an informal recap of one’s Tribal or Clan affiliation and familial relations when meeting someone new. Winona grew up on a Lakota reservation and now lives in an urban area to increase the credibility of her wisdom among people with diverse backgrounds and life experiences. To further develop Winona’s character and legitimacy, a Lakota actress voiced passages that Winona presents throughout the app, giving users the option to read along or listen to the content.

iCanQuit’s central “Urge Monster” metaphor was overhauled for IndigeQuit. Urge Monster is a classic ACT metaphor that suggests “dropping the rope” when you find yourself in a tug-of-war with a strong craving imagined as an urge monster. CAB members felt it too closely resembled colonial propaganda and demonizing language that had historically been used to oppress Native people, and evoked terrifying mythological creatures in Navajo culture called skin walkers. It was replaced with a new metaphor collaboratively developed by ACT clinicians and CAB members—Dropping Anchor. In this metaphor, resisting an urge is compared to rowing a boat against a river current. Rather than fighting the inevitable flow of nature to the point of exhaustion, the exercise suggests dropping the boat’s anchor and letting one’s urges come and go on their own.

#### New Content Additions

From the beginning of the redesign process, supported by step 1 findings, the distinction between commercial and ceremonial tobacco was considered a necessary change for cultural adaptation. A goal of IndigeQuit was to honor the ceremonial role of sacred tobacco and support the reclaiming of Indigenous practices through its use. A new section on ceremonial tobacco was added to the app and included an explanation of how it differs from commercial tobacco, the federal prohibition of its use until 1978 [5,8], and its roles in Indigenous culture as offerings, for healing, prayer, and ceremonies. In response to the suggested cultural refinements from step 2 interviews, a new “Preserving Traditions” motivation icon was designed and incorporated into the app, and all advertisement materials and app onboarding highlight that the app was created by *Natives for Natives.*

Feedback obtained through the step 2 interviews also urged the inclusion of a feature that facilitates connection between users. Members of the CAB strongly agreed and collaborated on creating a social component named Fireside Chat: a moderated message board where users post questions and share their quitting stories. CAB members noted this would particularly resonate in Indigenous cultures, where the pervasiveness of commercial smoking makes it common to feel alienated for deciding to quit. This feature might also help users enact their values of strengthening community and cultivating their Native identity, both of which IndigeQuit identifies as possible motivations that could bolster users’ resolve to quit. CAB members volunteered to moderate the Fireside Chat feature and were trained to respond to questions as Winona, providing intervention-consistent content and screening user stories for appropriateness before posting them for others to see. Users also have the option to message Winona (ie, study staff or CAB members) privately through the Fireside Chat feature, facilitating another layer of connection between app users and study staff.

Additional resources to support Native users were added on the recommendation of the CAB. These include links to a documentary on reclaiming sacred tobacco; information about the ways that commercial tobacco harms the environment; a powwow dancing video as an alternative form of exercise; an online resource that identifies whose ancestral homelands one resides on; and links to the American Foundation for Suicide Prevention and Mind Mental Health Support: Coping with Trauma. The CAB emphasized the prevalence of trauma among Indigenous communities and how it contributes to commercial tobacco use and addiction more broadly.

**Figure 2:**
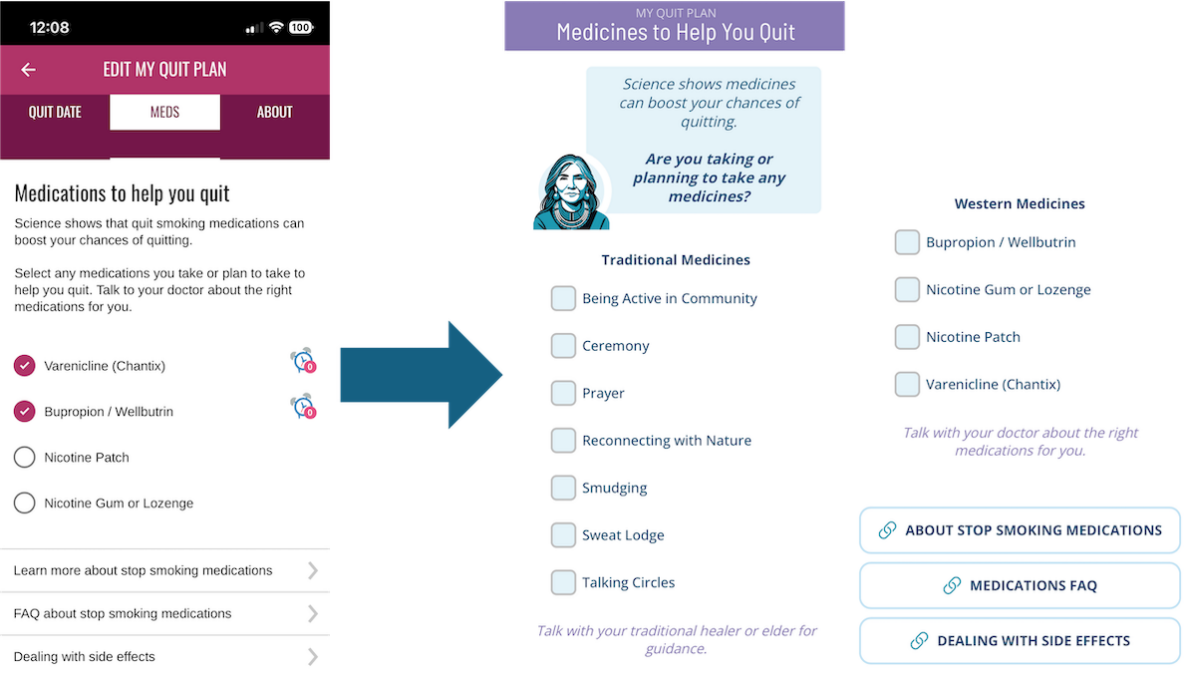
Medicines Tool. The left panel shows the original iCanQuit medication selection screen, which focused exclusively on Western pharmacologic smoking cessation treatments. The right panel shows the revised design, informed by participant and community advisory board feedback, which distinguishes between Western medicines (eg, nicotine replacement therapy, bupropion, varenicline) and traditional approaches to healing (eg, ceremony, prayer, reconnecting with nature, smudging, talking circles).

CAB members advocated that non-Western healing practices be presented alongside pharmaceutical support for commercial smoking cessation. Ceremony, prayer, reconnecting with nature, and sweat lodges were added to the Medications Tool and presented above pharmaceutical options, based on feedback that Indigenous people are more likely to gravitate toward traditional ways of healing than Western pharmacotherapy (Figure 2). The CAB felt it would be refreshing for Native users to see that their non-Western healing practices were treated as legitimate medicines alongside options such as nicotine replacement therapy, among others.

### Step 3: Beta Testing

#### Participant Characteristics

Seven participants were recruited from 6 US states (Alabama, Arizona, Minnesota, North Carolina, Oklahoma, and Washington). On average, participants were 42.6 (11.0) years old, and 2 (29%) were men. There were 6 Tribal affiliations represented: Choctaw, Lumbee, Navajo, Quapaw, Tsimshian, and White Earth Chippewa. At the time of the interview, all participants were currently smoking commercial cigarettes. Five participants completed all 6 daily diary entries, 1 participant completed 5 of 6 entries, and 1 participant completed 4 of 6 entries.

#### Summary of Results

Participants reported that the app was user-friendly and convenient to use. All participants expressed strongly positive views about the look and feel of the app and did not report any negative or critical feedback on this aspect. They praised the Native artistic touches, the user-friendly layout, and the app’s modern and refreshing feel. Participants also noted that the app was clearly tailored to their culture, citing elements such as the Medicine Wheel imagery, the use of Native lingo, and Winona (the guide avatar). Finally, participants appreciated the notifications with inspirational quotes and reminders to stay smoke-free throughout the day. Participant perceptions of each app feature from diary entries are presented in Table 1, along with representative quotes from 1-on-1 interviews.

**Table 1:** Quantitative rating summary and representative quotes for each app feature reviewed^a^.

App feature	Example question	Rating scale (1 to 5)	Ratings summary	Representative quote
Overall Experience	How would you rate your experience with the IndigeQuit app?	Poor to excellent	5 of 7 participants gave an experience rating of 5 across all or almost all entries, with 1 participant giving a rating of 4 on 1 day. 1 of 7 participants gave an experience rating of 4 across almost all entries, giving a rating of 3 on 1 day. 1 of 7 participants gave a mix of experience ratings between 3 and 5, giving a rating of 3 on 2 days, 4 on 3 days, and 5 on 1 day.	“There’s something special about this app that, for Natives, will make it better than any other.”
Information About Ceremonial Versus Commercial Tobacco	If you read about Ceremonial Versus Commercial Tobacco, what is the value of this information?	Not very to very valuable	3 of 4 participants gave a value rating of 5. 1 of 4 participants gave a value rating of 4.	“Having that historical information in there I think is good, especially for those that maybe don't know about ceremonial tobacco use to know that it's not the same. I know I've definitely been around Natives where we're off praying and it's like, ‘All right, who's got a cigarette?’ And we're just going to pop some tobacco out of a cigarette off on the side of a mountain or whatever…I know there's been a couple of us making jokes like, ‘Yeah, we're going to go smudge our lungs.’ Oh my god, that's so bad, but it's so common. So having that distinction in there I think is really good.”
Character Testimonials	How valuable are these stories?	Not very to very valuable	6 of 7 participants gave a value rating of 5. 1 of 7 participants gave a value rating of 3.	“It actually made it sink in a little bit more too, because after growing up being Native and everything, hearing stories is one of the best ways to learn.”
Tracking smoking, urges, and smoke-free days	How likely are you to continue tracking?	Not likely to very likely to continue tracking	5 of 7 participants gave a likelihood rating of 5. 2 of 7 participants gave a likelihood rating of 4.	“That was really helpful, especially giving me little quotes and little tips to actually, like little to-do techniques or tools to use…It's just a moment or at a moment's notice and you just let that moment just pass and you'll be okay.”
Push Notifications	How valuable are the notifications?	Not very to very valuable	4 of 7 participants gave a value rating of 5. 2 of 7 participants gave a value rating of 4. 1 of 7 participants gave a value rating of 3.	“When I got up in the morning, or when it was like a phrase or something, then I'd take a look inside the app, see where I was at…that was pretty cool. I liked that. It kind of got me up in the mornings.”
Track My Progress & Calendar Tracking	What is the overall ease of use of tracking your smoking and urges?	Not easy to very easy to use	2 of 5 participants gave an ease-of-use rating of 5. 2 of 5 participants gave an ease-of-use rating of 4. 1 of 5 participants gave an ease-of-use rating of 3.	“I liked it. I liked it, because it shows progress, and it shows the effort that I've made. That definitely, progress is very important for me, because I like to know how far I have come along, since I have implemented an app that is able to help me quit smoking.”
Journey Statistics & My Quit Plan	How valuable is this information?	Not very to very valuable	3 of 4 participants gave a value rating of 5. 1 of 4 participants gave a value rating of 4.	“I like how it was set up with the amount of money saved. I'm on a 6-day plan just for the testing. The milestone medals were pretty neat, just kind of keeps me busy. It kind of keeps my mind off of the urges and going out and smoking basically.” [edited for clarity]
Audio exercises	If you have listened to audio exercises, how valuable is the information given in the exercise?	Not very to very valuable	2 of 4 participants gave a value rating of 5. 2 of 4 participants gave a value rating of 4.	“You could just hit play and close your eyes and think that somebody's sitting there with you and is from your community and is talking to you, which is nice. I liked that.”
Fireside Chat	If you used Fireside Chat, what is the value of this feature?	Not very to very valuable	2 of 2 participants gave a value rating of 5.	“It's nice to hear from other people from other Nations to express their journey on a smoke-free life and using the app and everything.
Medication Tool	How useful was this information and reminder system?	Not very to very useful	4 of 5 participants gave a usefulness rating of 5. 1 of 5 participants gave a usefulness rating of 2.	“My favorite part has actually been the medications. To see prayer included and to see community included is crazy to me not because it’s crazy but because it’s unheard of to see those things considered in any journey surrounding something like stopping smoking, drinking, or even mental health with apps like these. It’s such a good thing to acknowledge these because I know for me, having tried other apps and the patches before, that at one point prayer and community was all I had but it felt like it wasn’t enough for these apps when documenting information because it isn’t gum or a medication physically. If it’s encouraging to me to get credit for these things I know it will be to others and if that’s all they have? To be credited for it can go a long way.”

^a^For summaries of ratings with fewer than 7 total participants, the remaining participants either did not complete the diary entry in which the question was included, had not yet encountered the app feature at the time the question was asked, or had their response excluded due to their rating explanation indicating they had thought the question was asking about a different app feature.

#### Positive Response to Cultural Tailoring

Participants unanimously felt that the app was designed for them and pointed to several culturally tailored features as the reason. These included the following: (1) stories from testimonial characters, which they found relatable and inspiring and which fostered a sense of community; (2) culturally tailored resources, such as information about ceremonial versus commercial tobacco, which they found informative and engaging; (3) character designs and the app’s inviting interface, which reinforced the sense that the app was truly created for them; and (4) the medicines tool’s recognition of prayer, ceremony, and community engagement as aids to quitting smoking. One participant said: “Right off the bat they're including prayer and ceremony and community. That’s not something that you see with the other apps. I really like that, because it felt like being seen and that recognition is there for something that typically isn't recognized...It goes a long way.”

Many participants also identified the guide character, Winona, as a key example of cultural tailoring. Responses to Winona were overwhelmingly positive. Participants described receiving guidance from her as feeling similar to being spoken to by an elder or older relative, particularly a grandmother. This association was viewed entirely positively and was often described as meaningful and cherished. One participant shared: “My grandma was kind of the same way. She told stuff in stories. She never got really angry. She just always knew the right time to talk and say certain things. So, it was pretty neat. The way I felt about it was getting up in the morning, having breakfast, drinking coffee, and sitting with her and talking. That’s the way it felt to me.” Participants were enthusiastic about Winona’s visual appearance, which they felt conveyed wisdom and healthy aging. They also found her storytelling humanizing and inspiring, making the content feel more authentic and helping them connect it to their own lives.

**Figure 3:**
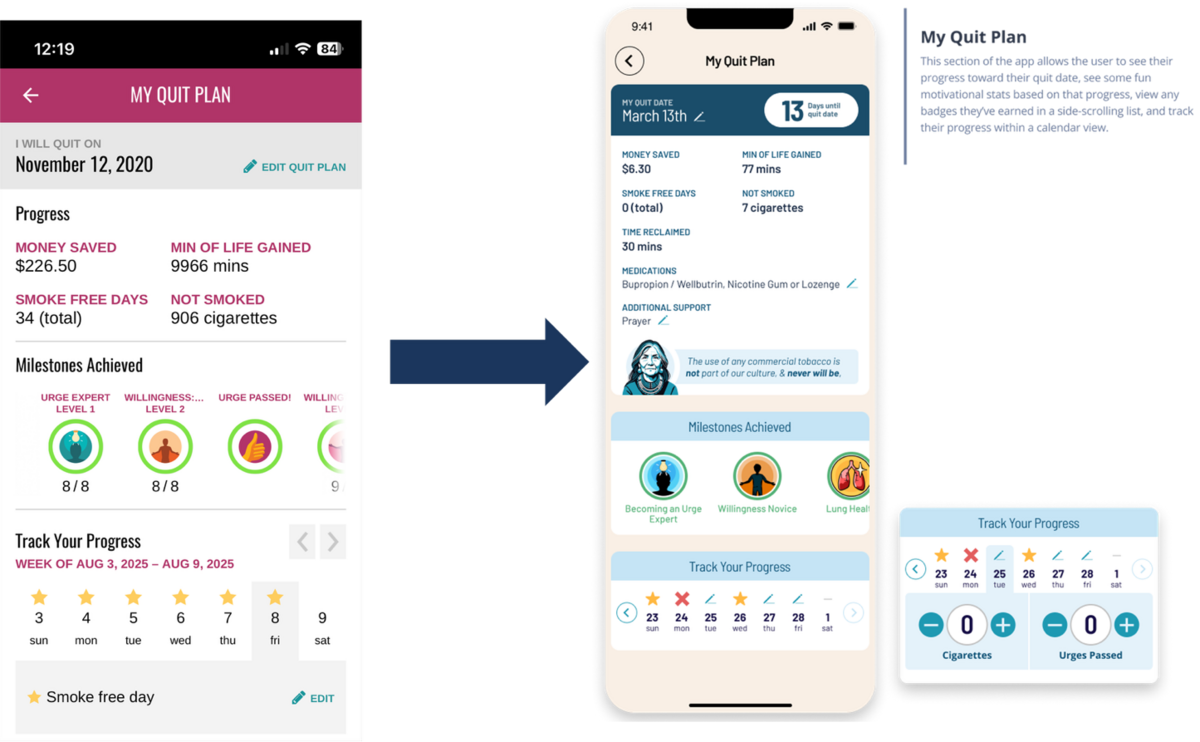
My Quit Plan. The left panel shows the original iCanQuit My Quit Plan feature, which summarizes quit progress using smoking cessation–related metrics (eg, smoke-free days, money saved, cigarettes not smoked) and calendar-based tracking. The right panel shows the redesigned version, which integrates supportive messages from the guide avatar, Winona, to reinforce motivation and engagement.

Quantitative content analysis of diary entries and 1-on-1 interviews with diary study participants generated 20 suggested changes to the app, summarized in Table 2. These results were presented to the CAB and stimulated discussion of additional adaptations. Suggestions supported by 3 or more diary study participants or strongly endorsed by CAB members were prioritized. The remaining suggestions were not implemented because of feasibility and budget constraints. The following changes were deemed both appropriate and feasible and were incorporated into the final version of the app: (1) content on vaping was added to the frequently asked questions (FAQ) section; (2) motivation icons were incorporated into My Quit Plan, and a link to update these motivations was added to the hamburger menu (Figure 3); (3) the number of daily push notifications was increased to 4, including additional content encouraging users to check in on urges; (4) tracking on the home screen was changed from “Yesterday’s Cigarettes” to “Today’s Cigarettes,” and a link to calendar tracking was added to the hamburger menu; (5) a field was added to the Quit Plan where users can update how much they spend on a pack of cigarettes; and (6) the Medications Tool was renamed the “Medicines Tool.”

**Table 2:** Suggested changes based on diary study participant feedback.

Number of participants endorsing each change, n	Suggested change
4	Include vaping frequently asked questions addressing the dangers of vaping and its health impact, how it compares to smoking cigarettes, and how vape nicotine is also commercial. Feature the motivations icons selected by the user more prominently and allow users to edit their list more easily. Increase standard to 4 push notifications per day. If feasible, allow the user to customize the number and time of notifications.
3	Change “Yesterday’s Cigarettes” counter to “Today’s Cigarettes.” Include more push notifications that prompt the user to check in on their urges. Explicitly state that IndigeQuit tools can also be used to quit/reduce other substances, especially alcohol.
2	Ensure more variety in the tips offered in the Urge Help feature. Have a location in the app where users can find the quotes provided in push notifications. Provide more organization in Fireside Chat, such as topic categories or a search bar for key terms. Prepopulate Fireside Chat with quitting stories. Include a Tools menu in the hamburger menu to help users locate specific tools after they are introduced. Include an editable field for price per pack next to “Money Saved” in “My Quit Plan”.
1	Rename the medications tool so it doesn’t discourage people who are not taking medications from interacting with it. Address how marijuana (including edibles) is often used as a replacement behavior. Allow a customizable write-in option under the existing “Additional Support” list (eg, ceremony, prayer, being active in community, other: ____). Add “time of day” to the list of trigger options in Urge Help. Add a new motivation icon called “Community.” Make a full monthly calendar option (or show at least 2 weeks at a time) to better see progress and potentially include more information about urges and triggers tracked in Urge Help. Rename “On the Path” to make it clearer that this menu item refers to content after your quit date. Add visuals to accompany the Leaves on a Stream exercise.

## Discussion

### Principal Findings

This study used a 3-step, user-centered CBPR approach to culturally adapt the iCanQuit app in collaboration with a CAB of AI/AN members, resulting in the development of the IndigeQuit app to help AI/AN communities quit commercial cigarette smoking. Principal findings indicated that honoring the Earth and nature, family and community, and cultural connectedness emerged as key themes throughout the iterative process and therefore became the focus of the app’s cultural refinements.

The main cultural adaptations included AI/AN imagery, refinements to avatar testimonials and intervention content (eg, incorporating “rez” phrases), and the development of new features and tools. New imagery features avatars representative of AI/AN community members, including the guide avatar Winona, who resembles a community elder, and the use of the Medicine Wheel as the basis for the home screen design. Culturally tailored content includes information on the distinction between ceremonial and commercial tobacco, resources and external links to support Native users (eg, a powwow sweat dancing exercise video and an online resource identifying whose ancestral homelands one resides on), a vaping FAQ, and messaging in recruitment and onboarding materials emphasizing that the app was developed by Natives for Natives. New functions include Fireside Chat to facilitate connection between users and a revised Medicines Tool that tracks both Traditional (eg, community engagement, ceremony, prayer) and Western medicines (eg, bupropion, nicotine patch).

The beta testing of the app, which included a 6-day diary study followed by 1-on-1 interviews with a nationally recruited sample of AI/AN adults who smoke commercial cigarettes, indicated that all participants responded positively to the app’s design, praising its Native modern, user-friendly layout and cultural tailoring through Native imagery, language, and the Winona avatar. Participants especially valued the inspirational quote notifications and reminders, with some intentionally keeping them on their home screens for continued motivation.

### Comparison to Prior Work

Digital interventions for smoking cessation are now ubiquitous. However, only a few studies have reported on the cultural adaptation of smoking cessation interventions developed specifically to meet the unique needs of historically underserved AI/AN communities [56-59]. Specific to digital smoking cessation interventions, Carroll et al [60] developed and pilot-tested a cultural adaptation of the NCI smoking cessation app “QuitGuide” for AI adults who smoke commercial cigarettes and want to quit, named “QuitGuide for Natives.” Their cultural adaptation process also included qualitative interviews with the sample population and a second set of interviews with tobacco treatment specialists and public health professionals working in AI community settings in Minnesota and at a Tribal health clinic in Wisconsin.

Importantly, while both the IndigeQuit and QuitGuide for Natives apps were culturally tailored to AI/AN communities, the key distinction is that IndigeQuit is based on ACT [47], whereas QuitGuide for Natives is based on the USCPG [61]. ACT for smoking cessation focuses on the acceptance of cravings to smoke, which is conceptually distinct from standard USCPG approaches that teach avoidance of cravings. In fact, the IndigeQuit RCT is currently testing the efficacy of IndigeQuit in comparison with QuitGuide.

Nonetheless, the suggested cultural adaptations in our study aligned closely with those identified in the qualitative study by Carroll et al [60]. For instance, the importance of ceremonial use of tobacco, being in community, and practicing cultural traditions emerged as important intervention components. Additionally, the portrayal of the Medicine Wheel in the app imagery was suggested in that study, along with highlighting that the app had been developed by Natives for Natives. While in our study we found that caring for the Earth and community were main motivators to quit, in the Carroll et al [60] study, legacy and heritage emerged as the most important reasons for wanting to quit.

One main distinction between the qualitative findings of Carroll et al [60] and ours was that grief emerged as the primary theme with regard to important triggers for smoking. The authors noted that a sense of historical trauma and oppression was related to the concept of grief. While grief, specifically, did not emerge as a theme in the qualitative interviews we conducted, the concept of historical trauma did emerge during our discussions with the study CAB. The CAB emphasized the prevalence of trauma among AI/AN communities and how it contributes to commercial tobacco use and addictive behaviors more broadly. We addressed this emergent theme by adding a link to Mind Mental Health Support: Coping with Trauma in the newly developed Resources Tool in the app.

### Community Impact

The findings from the study that we consider particularly important relate to the use of CBPR principles and the community impact that was integral to the entire process. The incorporation of CBPR principles to collaborate with AI/AN community members in their roles as the study CAB was crucial, given the team’s focus on culturally adapting the intervention to be more relevant, acceptable, and inclusive of AI/AN communities. This goal was achieved through qualitative feedback from nationally recruited AI/AN community members and CAB-driven adaptations.

The importance of building partnerships with community members and stakeholders cannot be overstated. Collaborating with the CAB provided us with constructive suggestions and helped establish a bottom-up approach to community participatory planning by prompting useful modifications to the suggested refinements in step 1. Their input, grounded in lived and professional experiences from diverse Tribal communities, ensured that the app was culturally tailored for AI/AN people broadly, increasing its relevance across Tribal communities. This helped us develop the suggested refinements to more effectively meet the unique needs of AI/AN communities. In the next phase of the study, we are currently implementing the IndigeQuit RCT in strong collaboration with the CAB throughout the duration of the trial and beyond.

### Strengths and Limitations

This study has the potential to advance research on the development of culturally tailored interventions for AI/AN populations. First, the use of CBPR principles within a transdisciplinary research and community team provided contextual relevance and helped address the unique needs of the priority population. Second, the comprehensive 3-step process and the use of both quantitative and qualitative data offer a practical example for future cultural adaptation studies. Third, the inclusion of a geographically diverse group of AI/AN individuals who smoke or formerly smoked commercial cigarettes, in addition to CAB members and AI/AN investigators representing diverse Tribal affiliations, provided diverse perspectives on Indigenous customs, traditions, and viewpoints. Despite the study’s strength in incorporating diverse perspectives, several limitations should be noted. As with all qualitative research, the findings are not intended to be generalizable to other populations and do not represent the full diversity of the 574 federally recognized AI/AN Tribes in the United States. Accordingly, the results should be interpreted as informing culturally grounded design rather than representing all AI/AN experiences.

Second, the findings should be considered in the context of the convenience sampling approach used in steps 1 and 2, which included prior iCanQuit RCT participants. This approach may have introduced selection or social desirability biases. However, these participants’ direct, in-depth experience with the original app uniquely positioned them to identify which elements required cultural adaptation to enhance relevance for AI/AN users.

Third, the study was not designed to prospectively assess thematic saturation, as the interviews were intended to elicit targeted, actionable feedback on specific cultural adaptation elements, and responses were largely consistent across participants. Although saturation was not prospectively assessed in steps 2 and 3, substantial thematic overlap was observed. Given the study’s narrow aims, high-quality and time-intensive diary data, and confirmatory analytic focus, the concept of information power supports the sufficiency of the sample despite limited generalizability [62,63].

Finally, step 1 interviews were conducted in September 2022, whereas the primary 3-step development process occurred between June 2024 and August 2025. This time gap reflects the iterative nature of culturally grounded intervention development, including securing funding, establishing the CAB, and conducting subsequent qualitative work. Importantly, findings from 2022 were reexamined and confirmed through later CAB engagement and diary studies and remained relevant because they reflected enduring AI/AN values and practices, such as the central role of culture, spirituality, family, and community as motivators; the importance of a representative guide character; and the distinction between ceremonial and commercial tobacco use.

### Conclusions

The user-centered and CBPR development process yielded a comprehensive and culturally tailored app called IndigeQuit. The iterative development process led to improvements in app imagery, design, content, and features, enabling users to connect more deeply with the program. Our next step is to test IndigeQuit against QuitGuide in an RCT among 776 nationally recruited AI/AN adults who smoke commercial cigarettes and want to quit.

The principles of cultural adaptation, community engagement, and value-based motivation—while grounded in AI/AN cultural contexts—may inform the design of smoking cessation and digital health interventions for other demographic groups.

The processes presented here provide a guide for developing culturally tailored commercial smoking cessation interventions designed to meet the unique needs of priority populations experiencing tobacco-related health inequities.

## Data Availability

The data will be shared on a reasonable request to JBB (jbricker@fredhutch.org).

## Appendix

Multimedia Appendix 1 Step 1 interview guide.

Multimedia Appendix 2 Step 2 interview guide.

Multimedia Appendix 3 Step 3 interview guide.

## Abbreviations

ACT: acceptance and commitment therapy
AI: American Indian
AN: Alaska Native
CAB: community advisory board
CBPR: community-based participatory research
FAQ: frequently asked questions
HIPAA: Health Insurance Portability and Accountability Act
NCI: National Cancer Institute
OR: odds ratio
RCT: randomized controlled trial
USCPG: US Clinical Practice Guidelines
