# Supplementary material for: Development of a Culturally Adapted Smartphone App (IndigeQuit) Designed to Help American Indian and Alaska Native People Quit Commercial Cigarettes: User-Centered Mixed Methods Study
Source: JMIR Form Res. 2026 Mar 24;10:e88768. doi: 10.2196/88768 (PMC13058535; doi:10.2196/88768)
Supplement: Multimedia Appendix 1 [file formative_v10i1e88768_app1.docx]

**Multimedia Appendix 1.** Step 1 Discussion Guide

AI/AN iCanQuit Discussion Guide (2022)

Method: 1:1 remote moderated interviews with participant slides (discussion guide mirrors prompts in slides)

Schedule

| TOPIC/SCENARIO | TIME ALLOTTED | CUMULATIVE TIME |
| --- | --- | --- |
| Introduction and informed consent | 3 minutes | 3 minutes |
| Warm up - learning about tribal affiliations and culture | 5 minutes | 13 minutes |
| App sentiment ratings | 3 minutes | 16 minutes |
| Feedback on the app | 40 minutes | 45-46 minutes |
| Session Wrap Up | 5-7 minutes | 50-60 minutes total |

**INTRODUCTION & ICEBREAKER (3 minutes)**

[Moderator to explain setup and protocol, think aloud procedure]

- Consent form
- Start recording

###

### **Slide 7 - Warm up (up to 5 minutes)**

**Spend time on this screen learning about the participant - Tribal affiliation and cultural identification**

If you do identify with your tribe or participate in AI/AN traditions, can you tell us more?

What traditions have you participated in?

What is meaningful about these traditions?

How often do you participate in these traditions?

Are there any cultural AI/AN cultural traditions and principles that resonate with you?

ACT Prompts:

The notion of ‘openness to one’s internal experiences?

What does healing look like in the AI/AN culture?

How do you to recognize and manage difficult thoughts?

What does “Living in the present” look like for you on a daily basis?

### **Slide 8 (10 minutes or more)**

**Spend time on this screen learning about the participant’s experience with traditional tobacco. Incorporating traditional Native values (e.g., sacredness of tobacco)?**

**Suggested prompts:**

- What is your understanding of traditional Native values, especially around tobacco?
- Have you experienced any ceremonial use of tobacco?
- What is the cultural role of tobacco in your tribe?
- Is there any education around the use of traditional tobacco?

**Additional questions:**

- What do you think is the difference between traditional ceremonial use and recreational use of commercial tobacco and combustible cigarettes (e.g., adding ‘commercial’ in front of ‘tobacco’ and ‘cigarettes’)
- How important is this to find in the app?
- **Framing cessation as “restoring respect for tobacco”?**

### **Slides 10 -12 Ratings and sentiment**

**Slide 10** - On a scale of 1- 5; 1- not satisfied at all; 5 - very satisfied

If you have used any apps to quit smoking in the past, how satisfied are you with the experience of using these apps?

#### **Slide 11** - How motivated are you to quit smoking in the next 6 months?

#### **Slide 12** - How confident are you that you can quit smoking in the next 6 months?]

### **Slides 13-22 Feedback on the iCanQuit app**

#### **Slide 14**

Spend time introducing the iCanQuit app and ask about smoking behaviors

Prompt:

Now I am going to share my screen to show you an app called iCanQuit. You may have already downloaded and seen the app, and that is great. This is a quit smoking app designed for the general public.

Our goal today is to understand how we may adjust and tailor the app for the American Indian or Alaska Native population, using stories, language, colors and the overall design. Any information that you can share that will help us make the app attractive and relevant to this population would be greatly appreciated.

Any stories, or anything that comes from your experience is exactly what we are looking for. There are no right or wrong answers. Try to imagine this app as if you built it for someone like you.

Have you ever used the iCanQuit app? If so, what was your experience like?

How long did you try to use the app?

#### **Slide 15 (3-4 minutes)**

Please take a look at the onboarding screens.

Does this feel like this was made for you?

If so, what do you like about it?

If not, what can be changed or adjusted so you feel that it is made for you?

Is there anything that should or can be included from your culture that would make this more relevant to you?

#### Slide 16 **Feedback on Nancy (up to 10 minutes)**

We’d like to know your impressions about Nancy and how we might change Nancy to better serve the needs of AIAN smokers. (Show Nancy’s “Allow Me to Introduce Myself” and play the 1:42 long audio)

What are the things that work for you about Nancy?

As an AIAN, what would you change if you could, to make Nancy someone you would want to guide you?

- appearance
- voice?
- name?
- What qualities would show a resemblance to an AI/AN person?

Please read Nancy’s story.

- How relevant is Nancy’s story to you?
- Is there anything that can be changed to make Nancy’s story more appealing or interesting as an AI/AN?

#### **Slide 17 Feedback on the title and colors (up to 5 minutes)**

- From an AI/AN perspective, what title would make it clear that this app is made for American Indians and Alaska Natives people who use commercial tobacco or smoke cigarettes?
- Do you have any apps or other systems that are tailored to AI/AN people that you really like? What do you like about it?
  - How does it make you feel that it is tailored for you?
  - What are the things that it does that you like?
- Are there any colors or visual parts that might make this app feel tailored for you?
  - prompts: Sacred colors in app design - black, white, yellow and red (embody concepts such as the four directions, four seasons, and sacred path of both the sun and human beings)?

#### **Slide 18 Feedback on language, phrases, and greetings (up to 7 minutes)**

Prompt: please take some time to read through the titles and headlines you see here.

Focus on:

**Becoming an Urge Expert**

**Are You Willing**

**What’s Inspiring You to Quit.**

Is there a way that we can incorporate Native language - for example, use tribal language for common phrases and greetings that would make this app feel like it was made for?

Can you give us any examples of a common greeting that would make sense for this app?

How much do the messages about urges and willingness speak to you? Is there any way the language could be changed so it speaks to you better?

#### **Slide 19 Feedback on testimonials/stories (7-10 minutes)**

Prompt: Please take a look at Ty’s story here.

- In AIAN culture, what do stories teach you about changing your behavior?
- How would you include storytelling by other AI/AN people who were successful at quitting the use of recreational tobacco, including cigarettes?
- How appropriate or meaningful would it be to have stories told by an elder? What kind of impact/weight does this have on how someone of AIAN heritage when trying to quit smoking?

#### **Slide 20 - Feedback on the perception of medication for quitting smoking.**

#### Note: there is some evidence that AI/AN are less aware of potential benefits of medication (5-7 minutes)

Prompt: What things do you know about medications to help people quit smoking?

- Is this something that you have used in the past?
- How common do you think that medication is used in AI/AN people to help them quit smoking?
  - How do you feel about using medications to stop smoke?
  - What are ways that we can talk about the benefits of medication to AI/AN smokers?
  - What concerns do you have about offering information about medication?

#### **Slide 21 Conversation about culture and connectedness (leads into ACT acceptance and use) (5 minutes)**

Prompt: Can you tell us your take on Native Culture and Connectedness?

What does this look like in your life, if at all?
Are there specific ways that this shows up in your life, technology, relationships, etc?

How do you connect with other people who identify as AI/AN who are on a quit journey or have been successful at quitting smoking?

Is there a spiritual approach to quitting smoking?

#### **Slide 22 Open ended questions (5-7 minutes)**

**Final Questions**

1. Is there anything in this app that you find uncomfortable?
2. Is there anything you’d like to add that better represents your culture?
3. Does this app content represent traditions of multiple tribes? If so how? If not, what is missing?
4. How would you talk about the idea of being open and willing to have cravings to smoke?
5. How would you talk about the idea of living according your values?

#### **Slide 24 - Ratings for proposed changes**

Prompt: We’d like you to rate how important each of these proposed changes are for the future iCanQuit app. Please give us your honest rating from 0 (not at all important) to 5 (extremely important)

**Changing the…**

Colors

Fonts

Illustrations

Language

AI/AN storytelling

Look and feel of Nancy

**Participant Gratuity and thank you.**

**
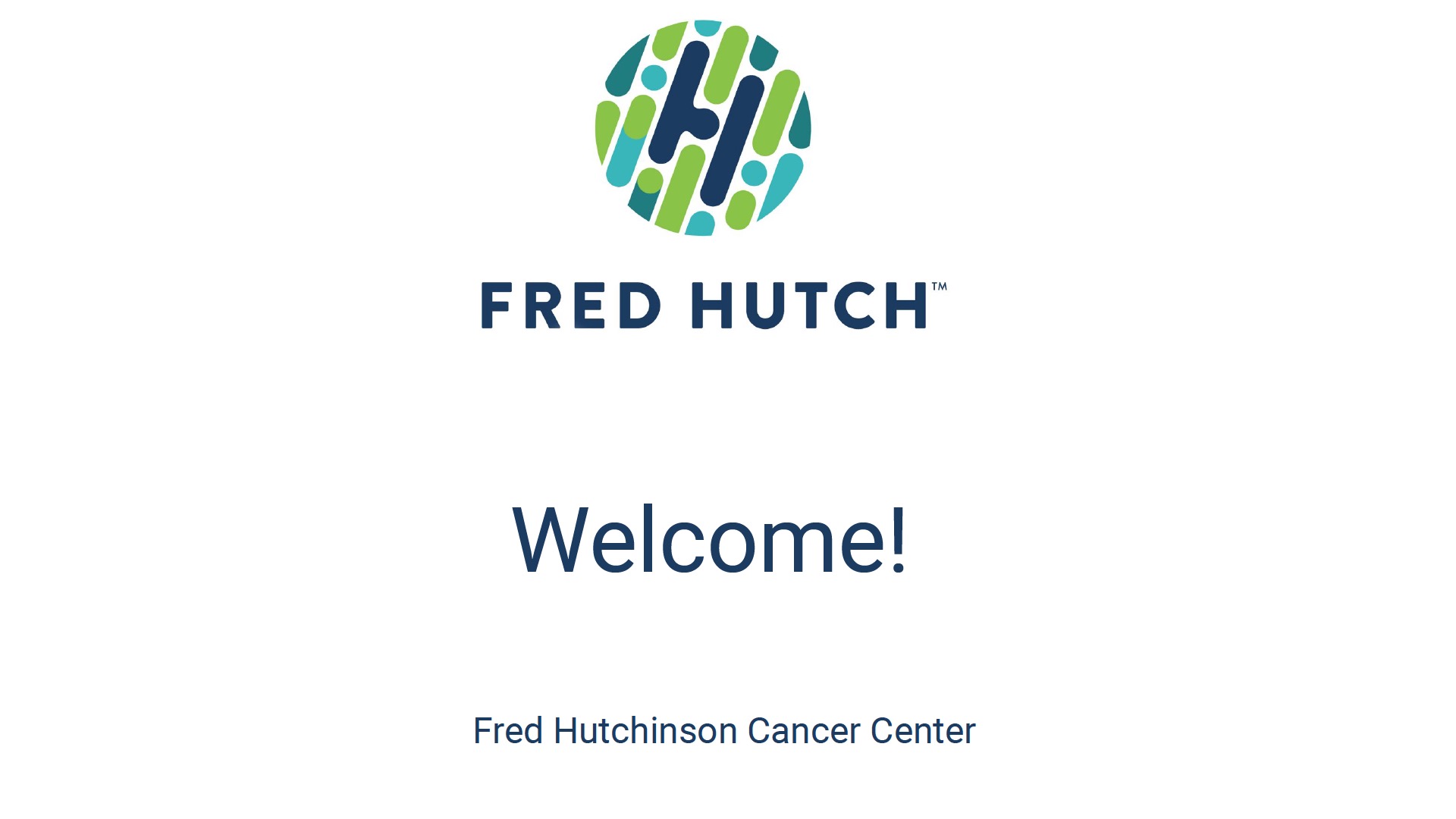

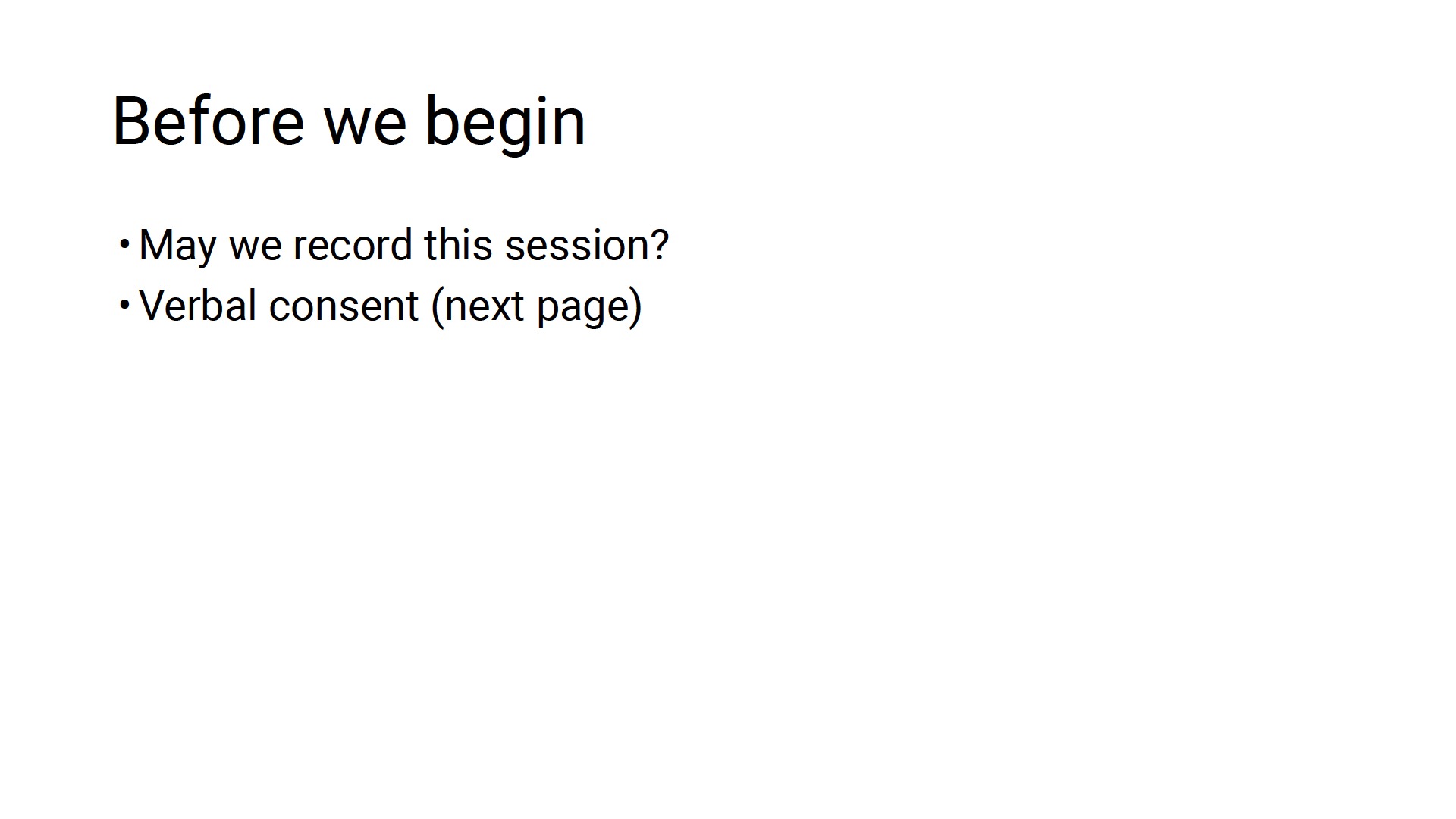

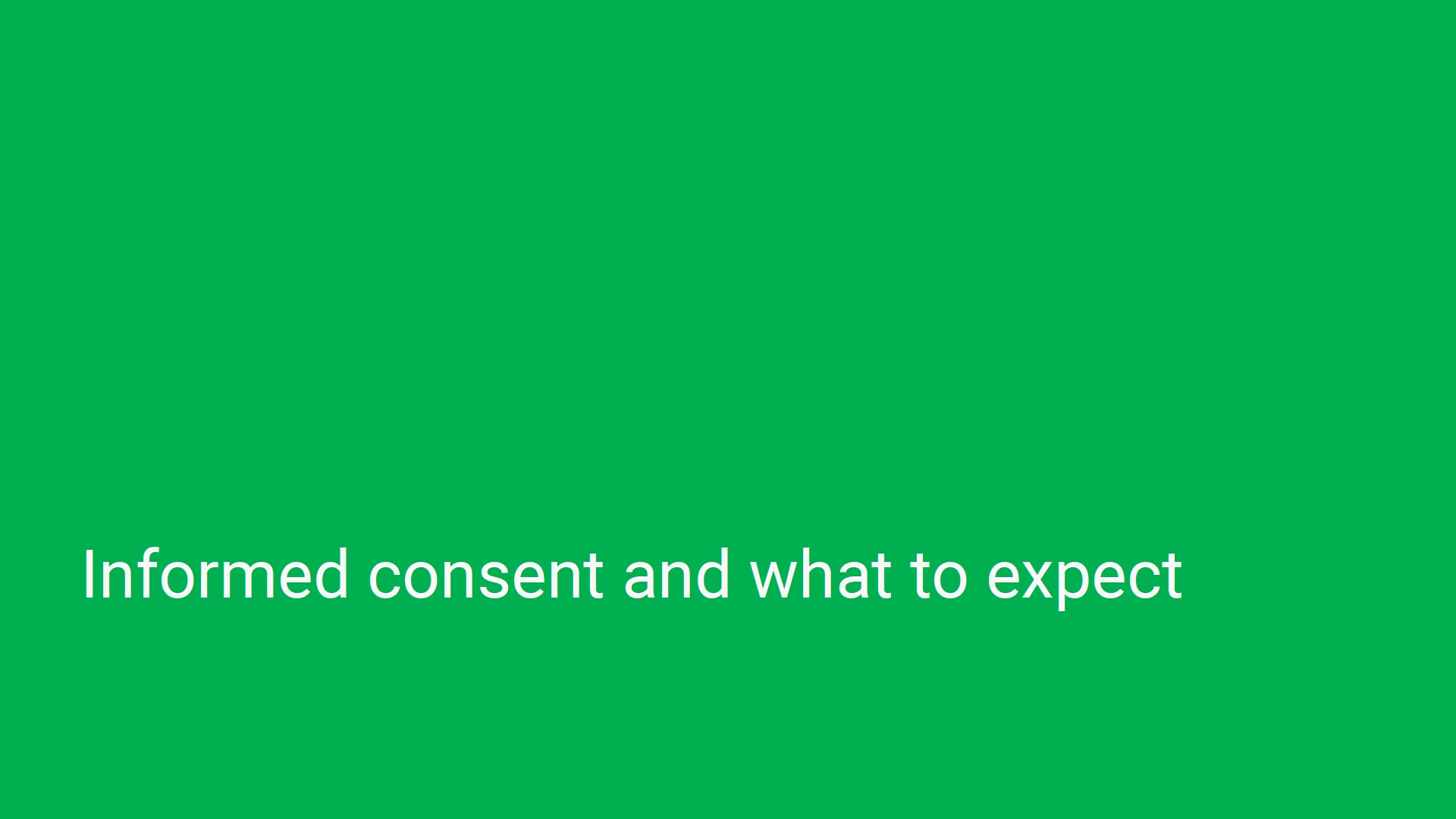

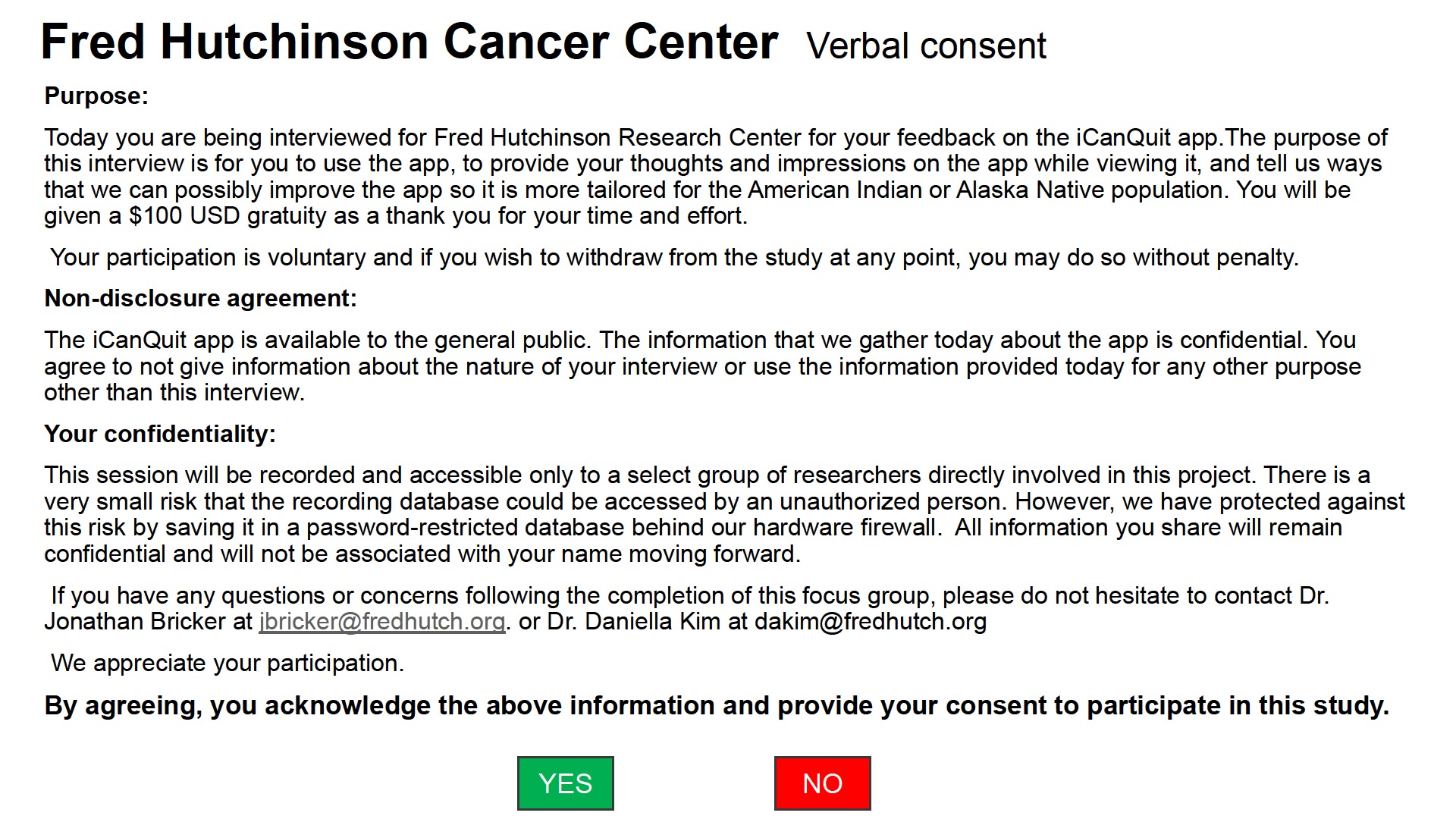

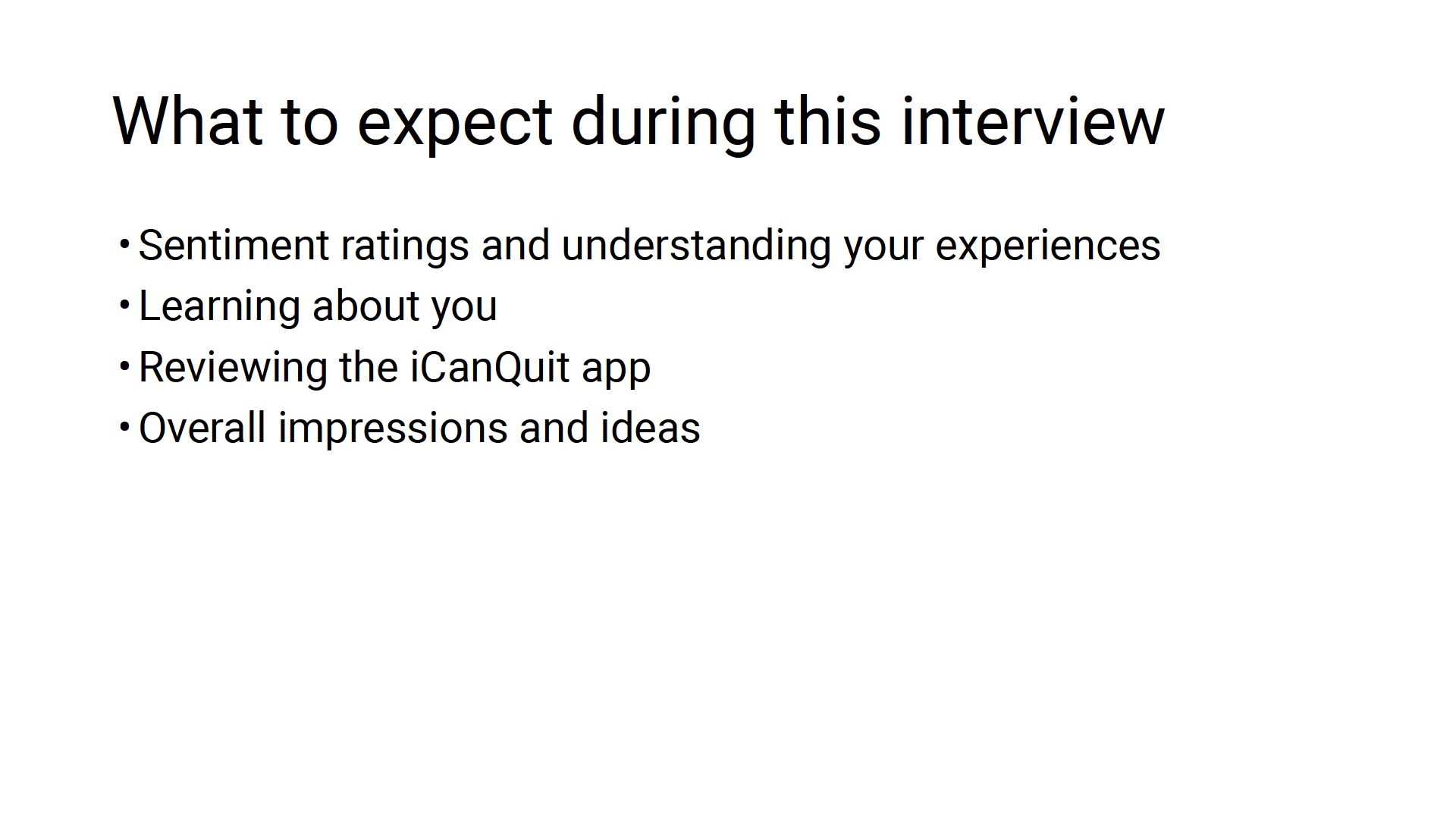

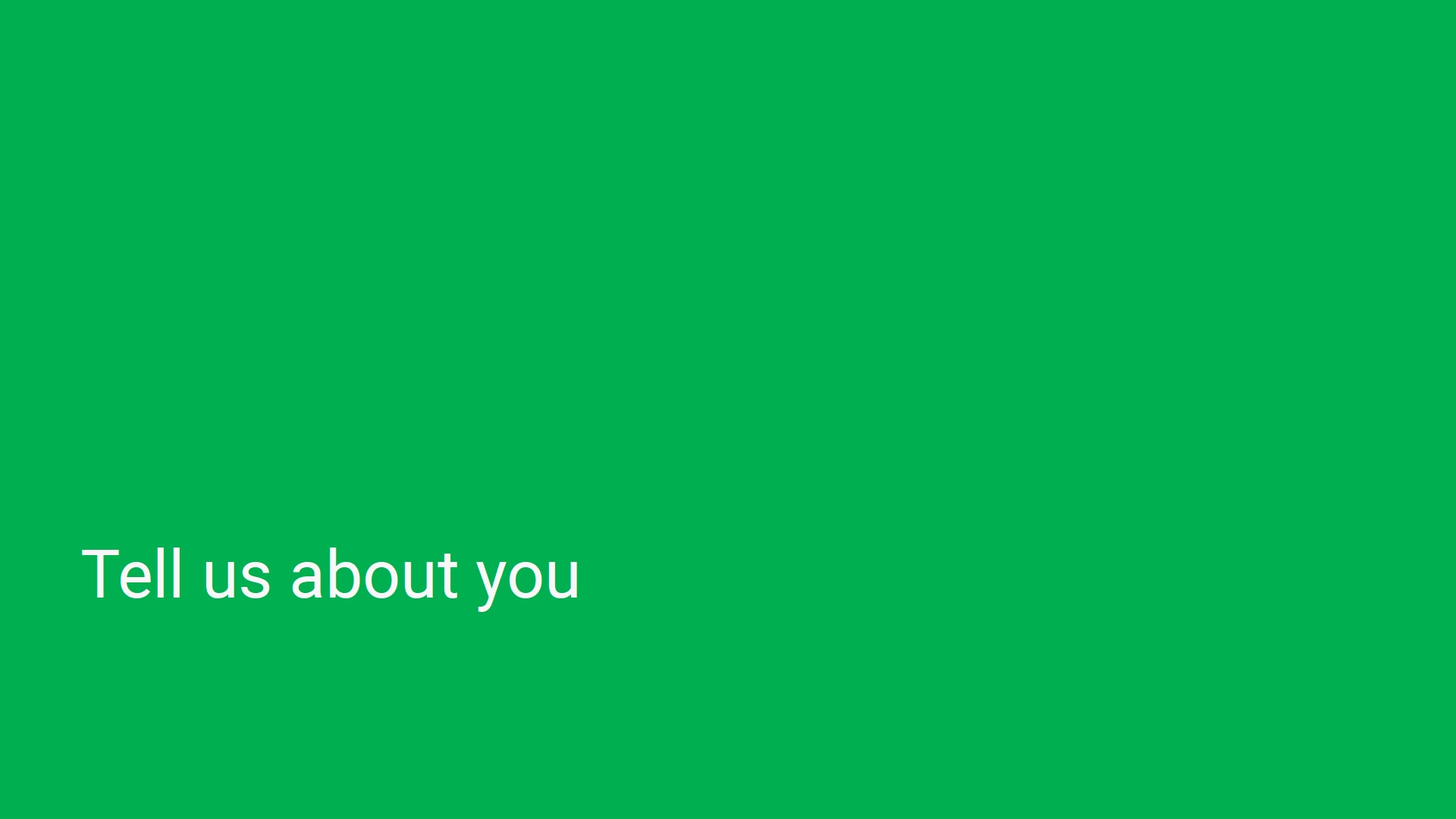

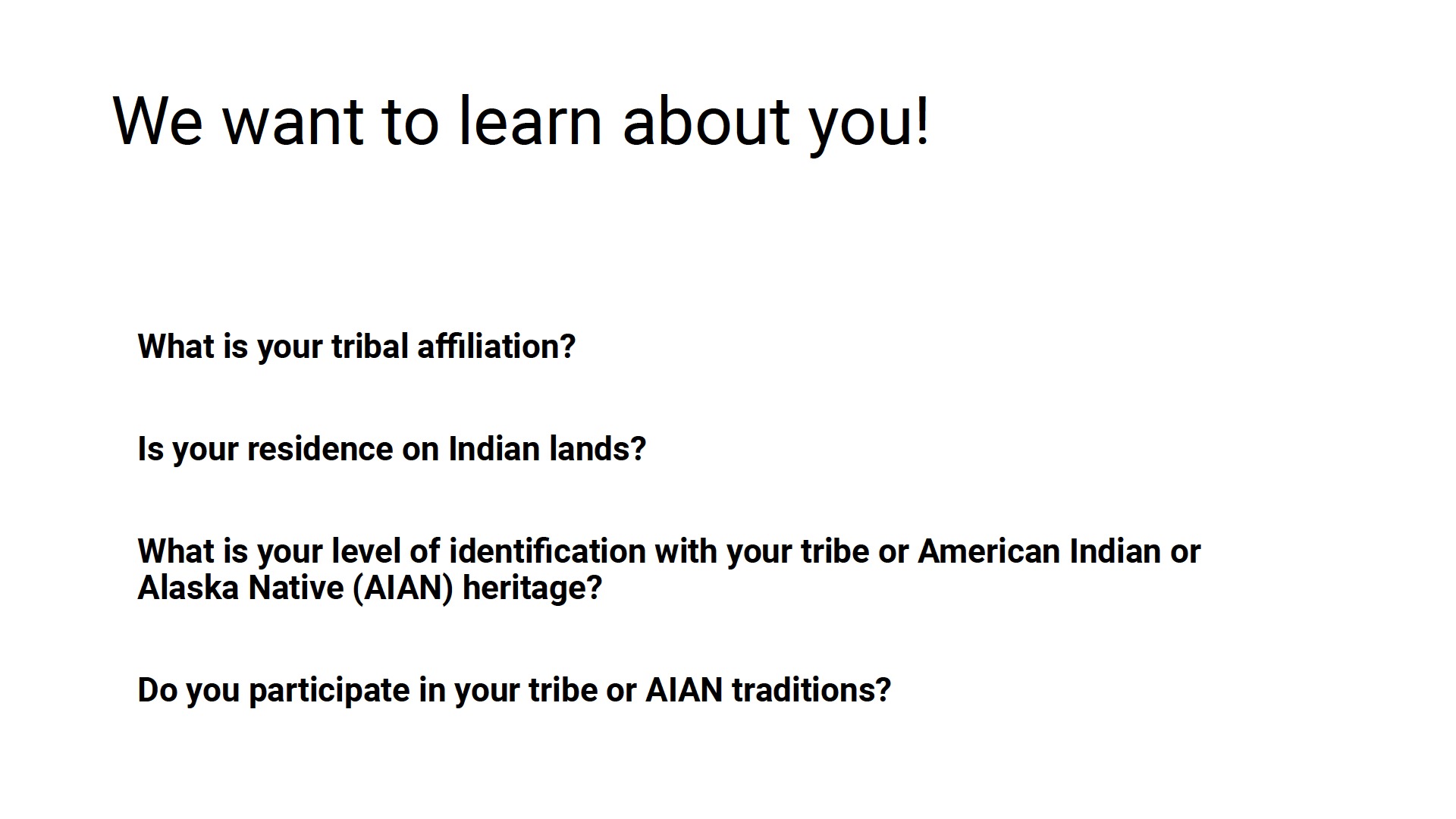

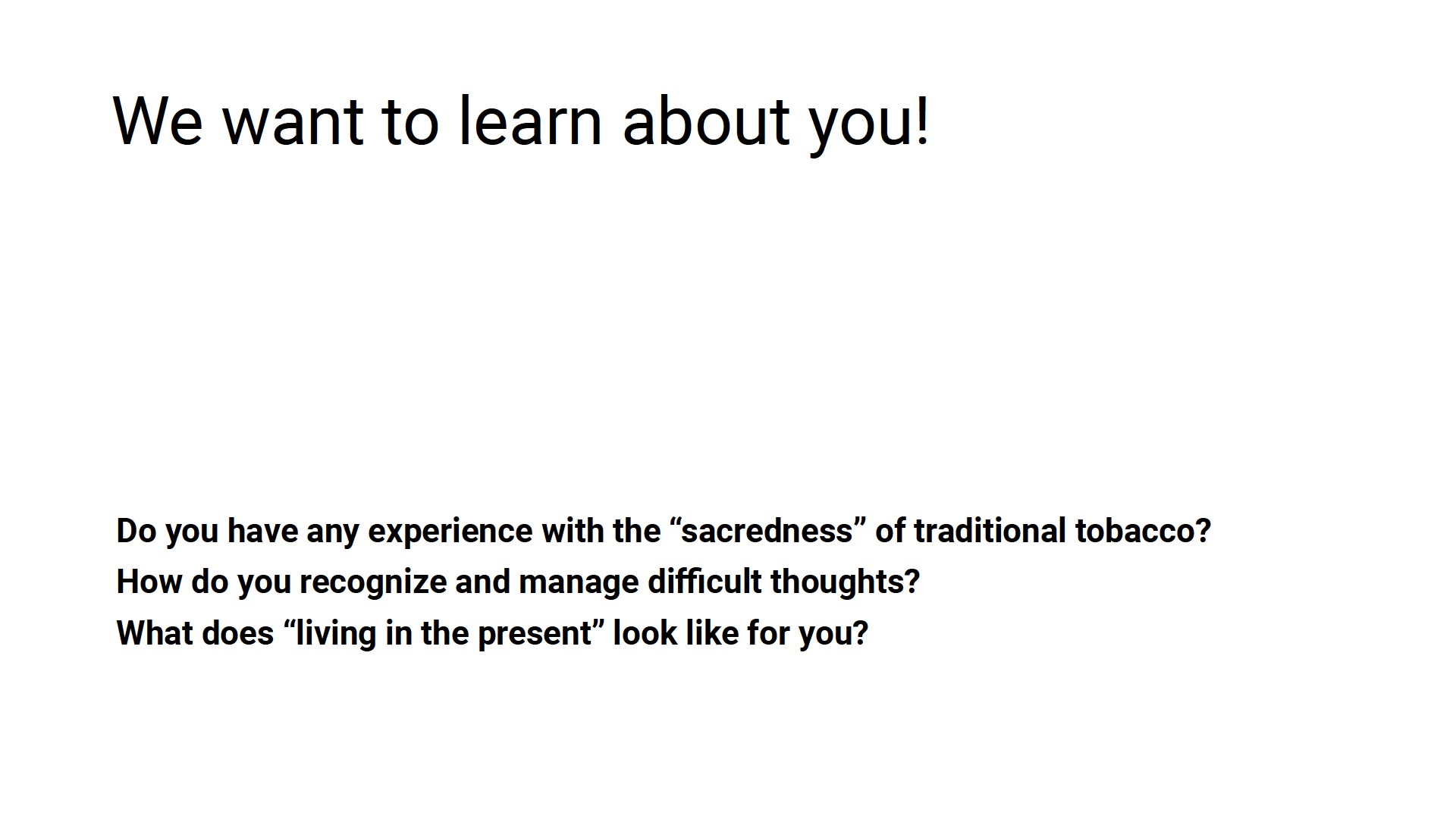

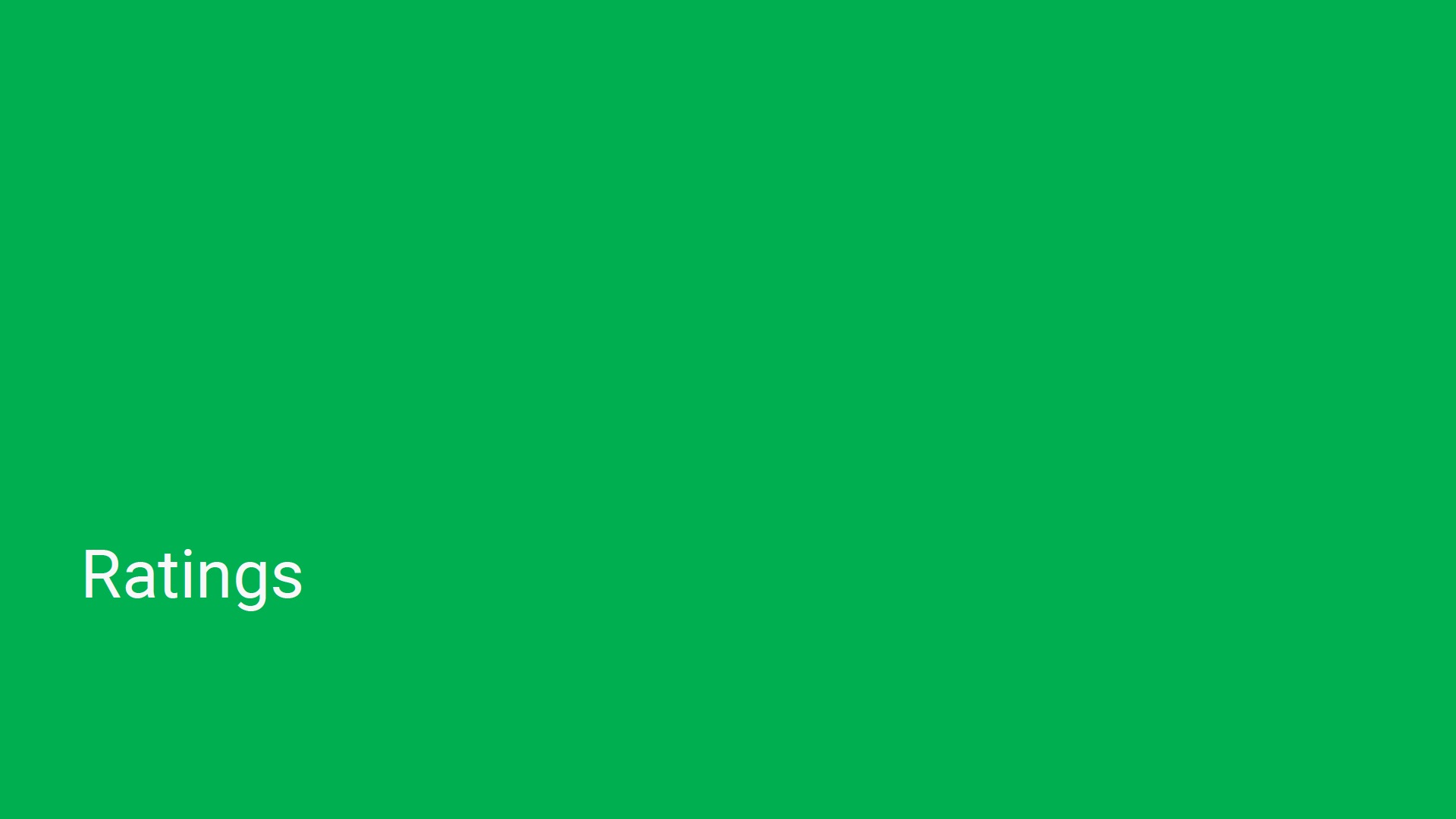

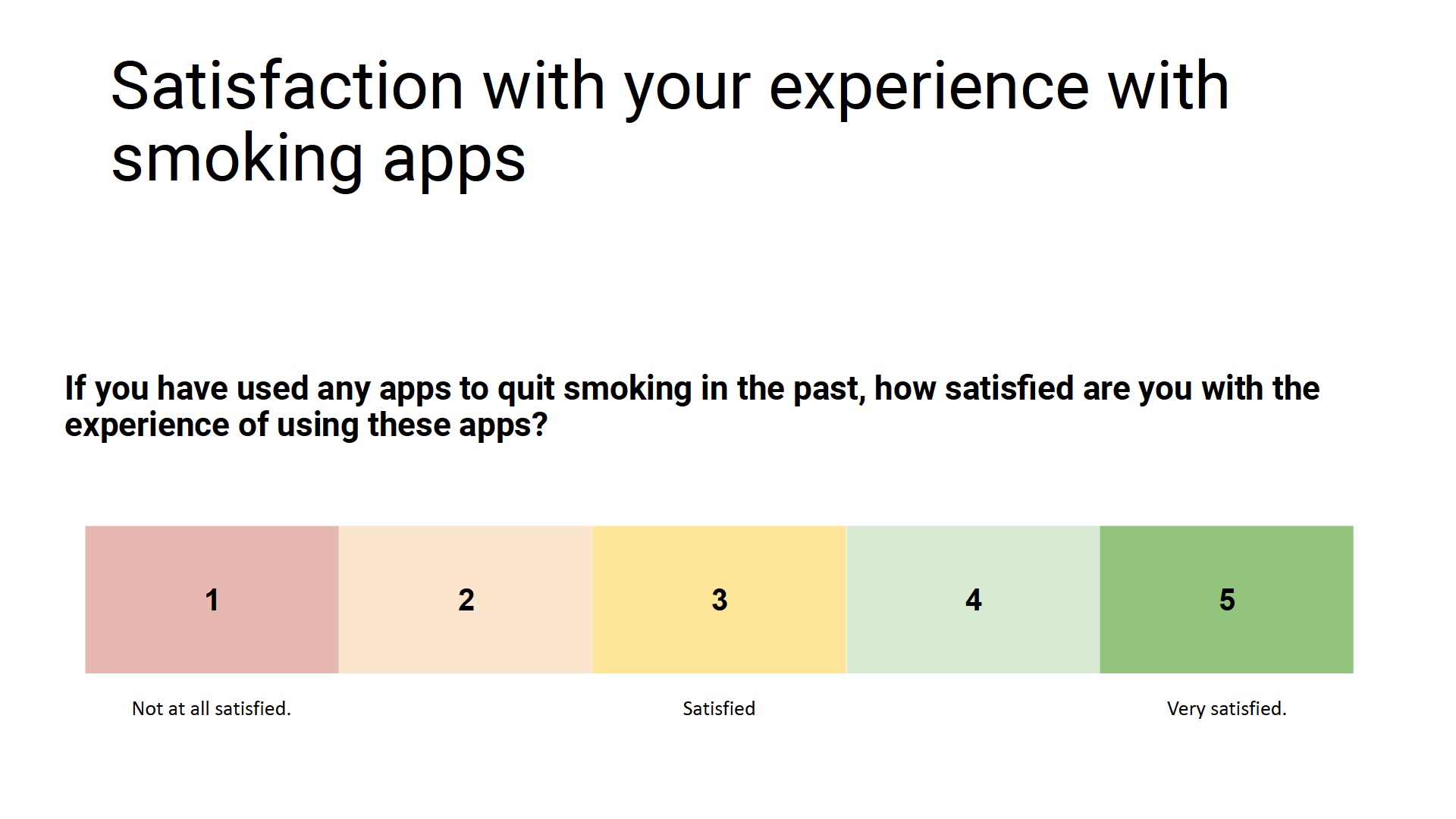

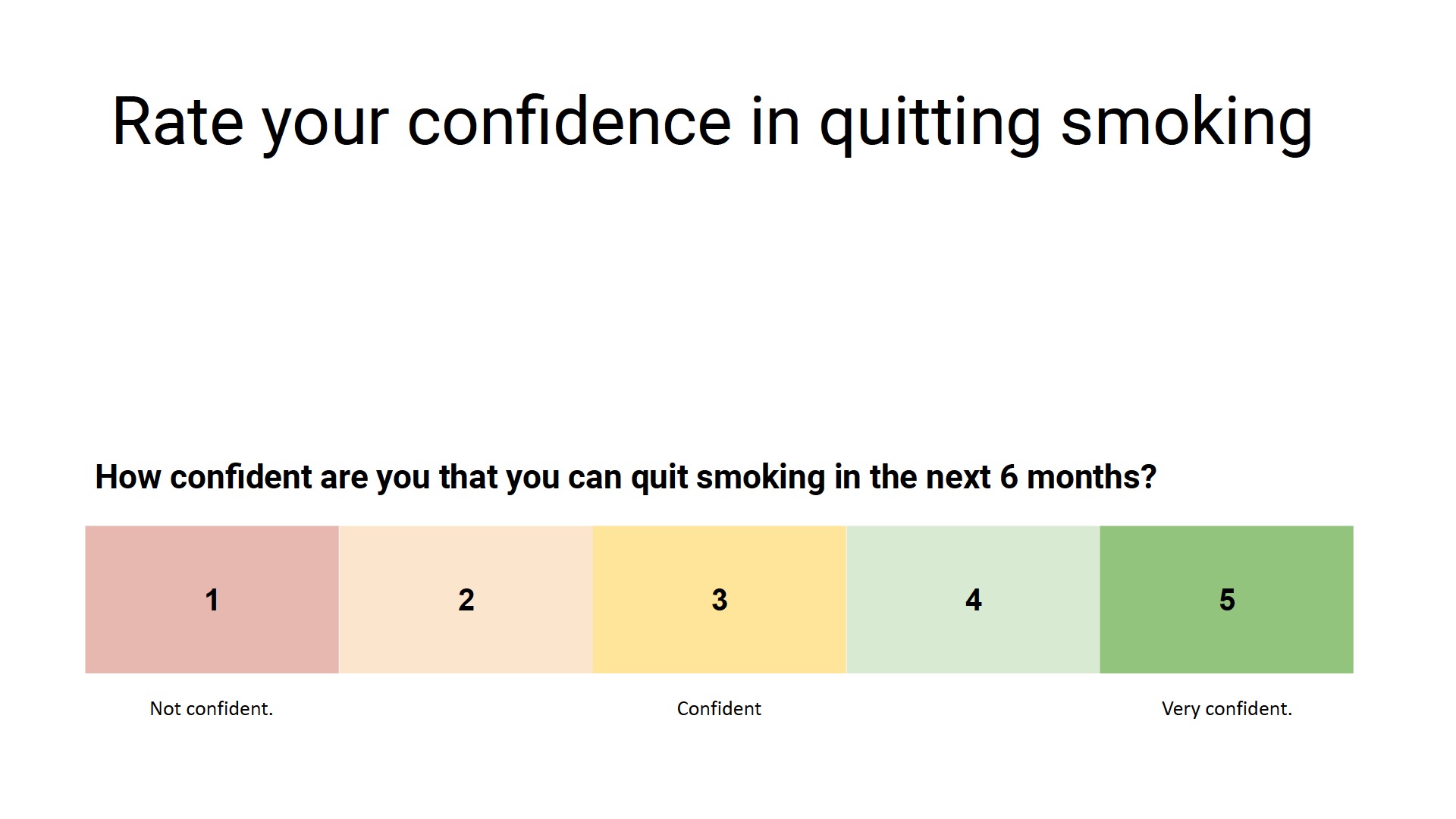

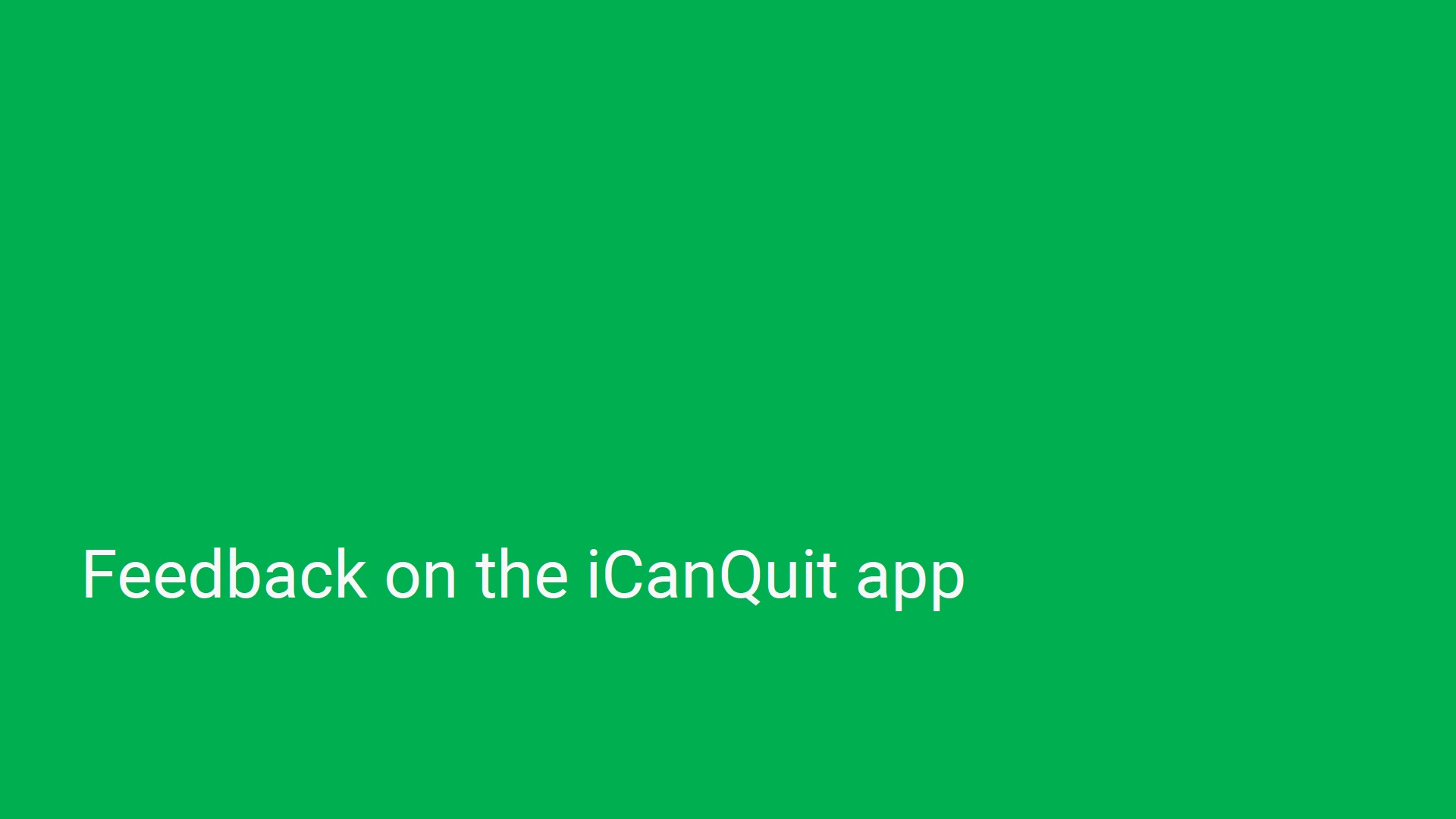

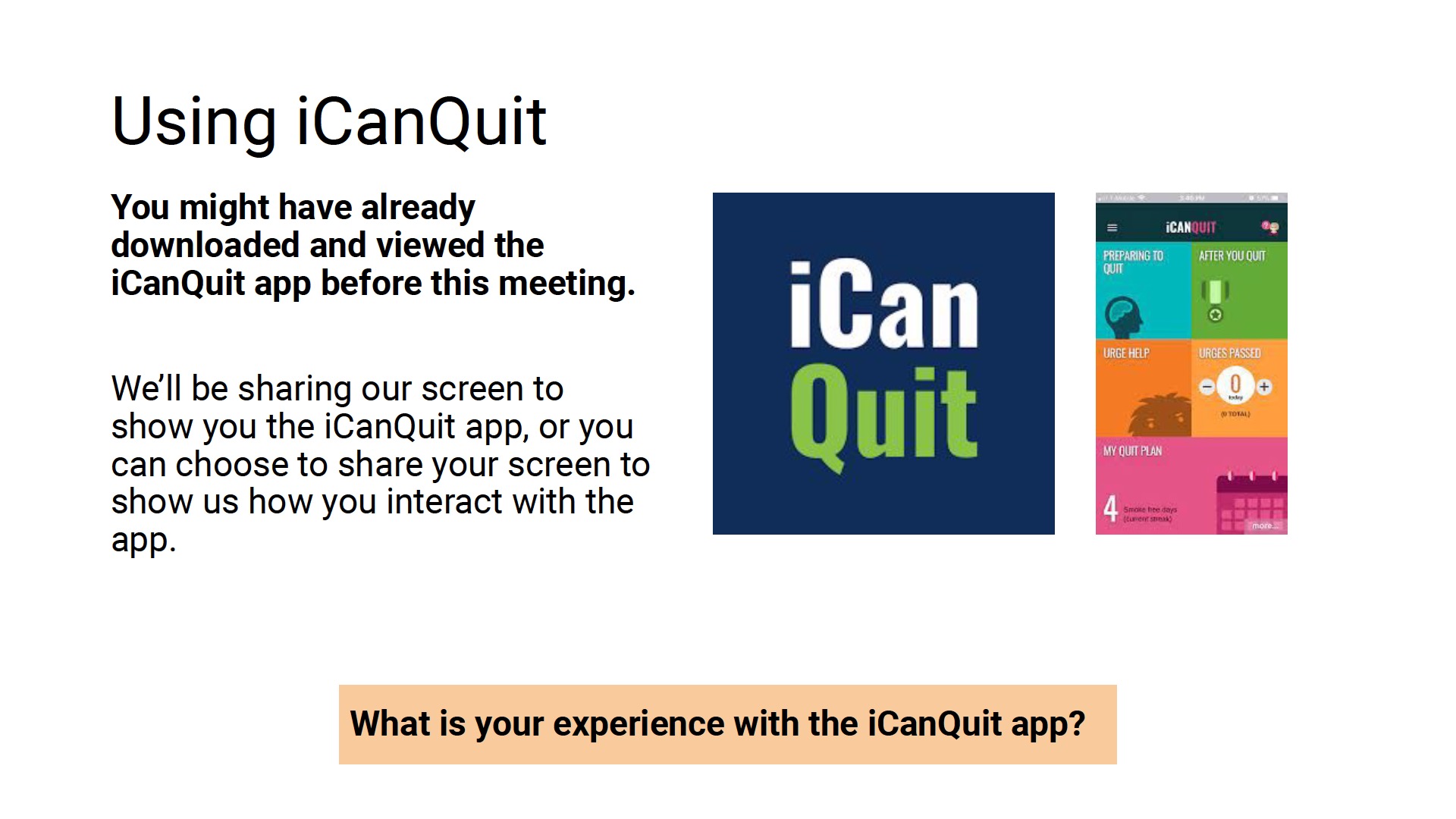

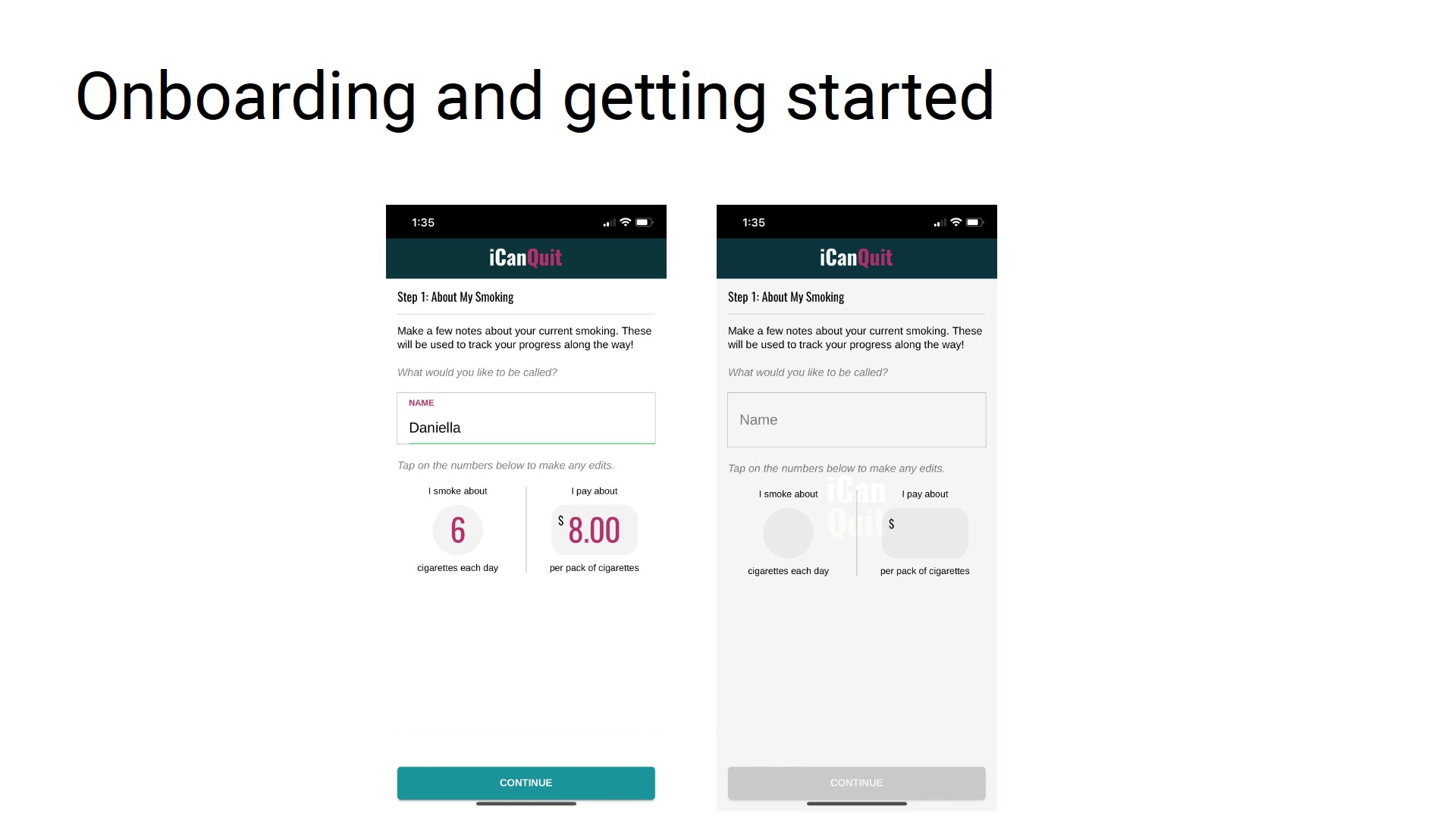

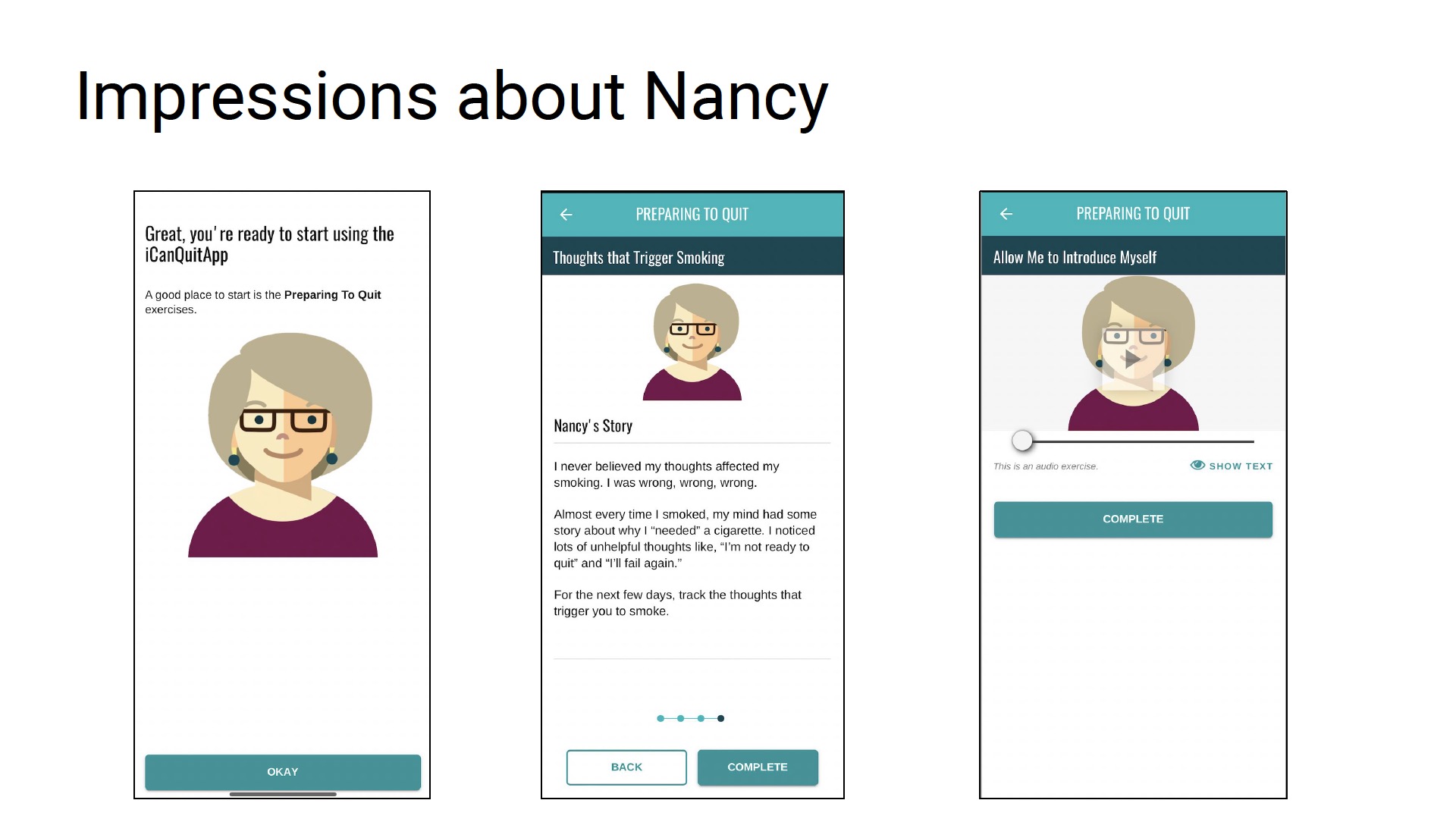

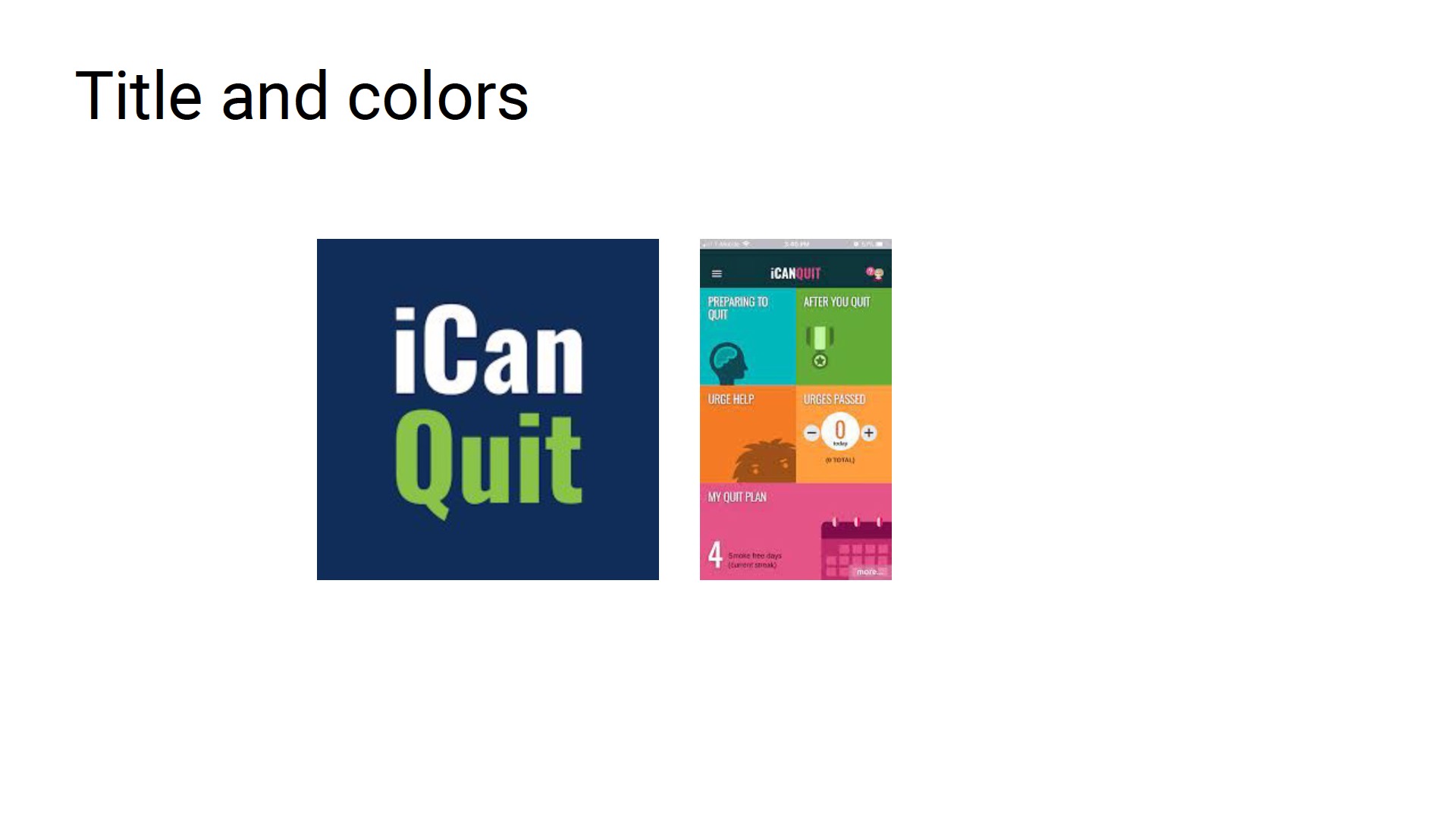

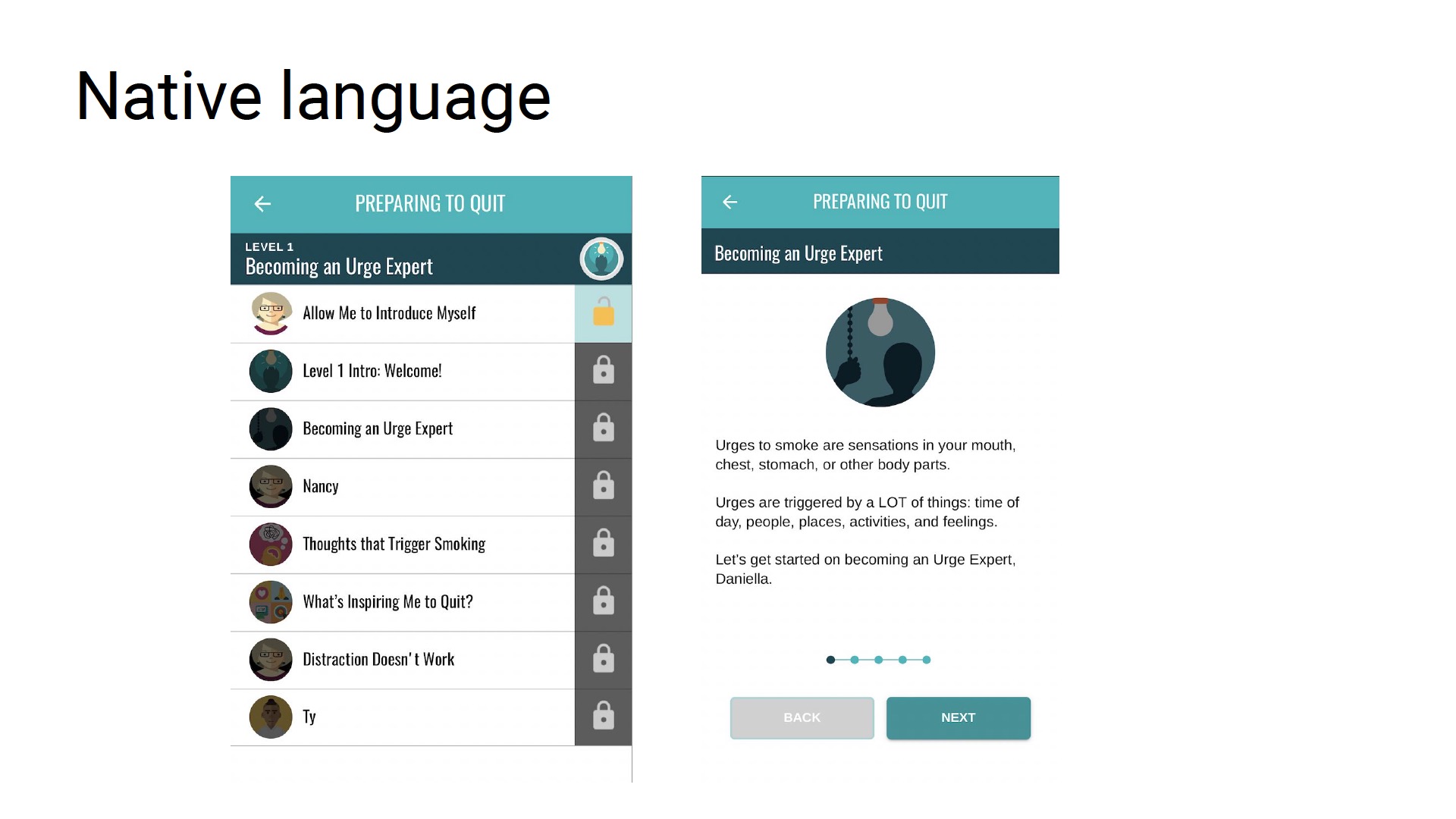

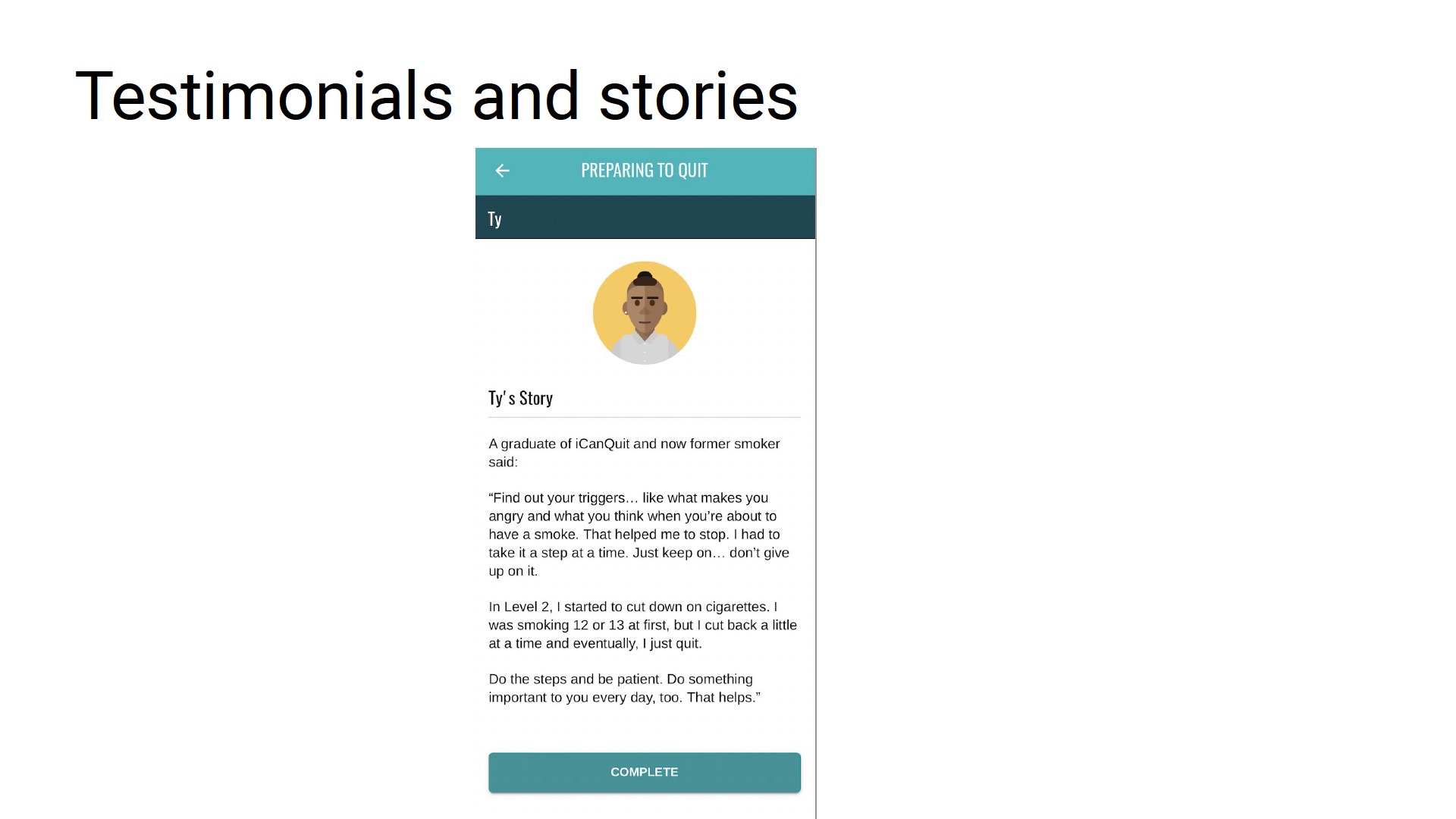

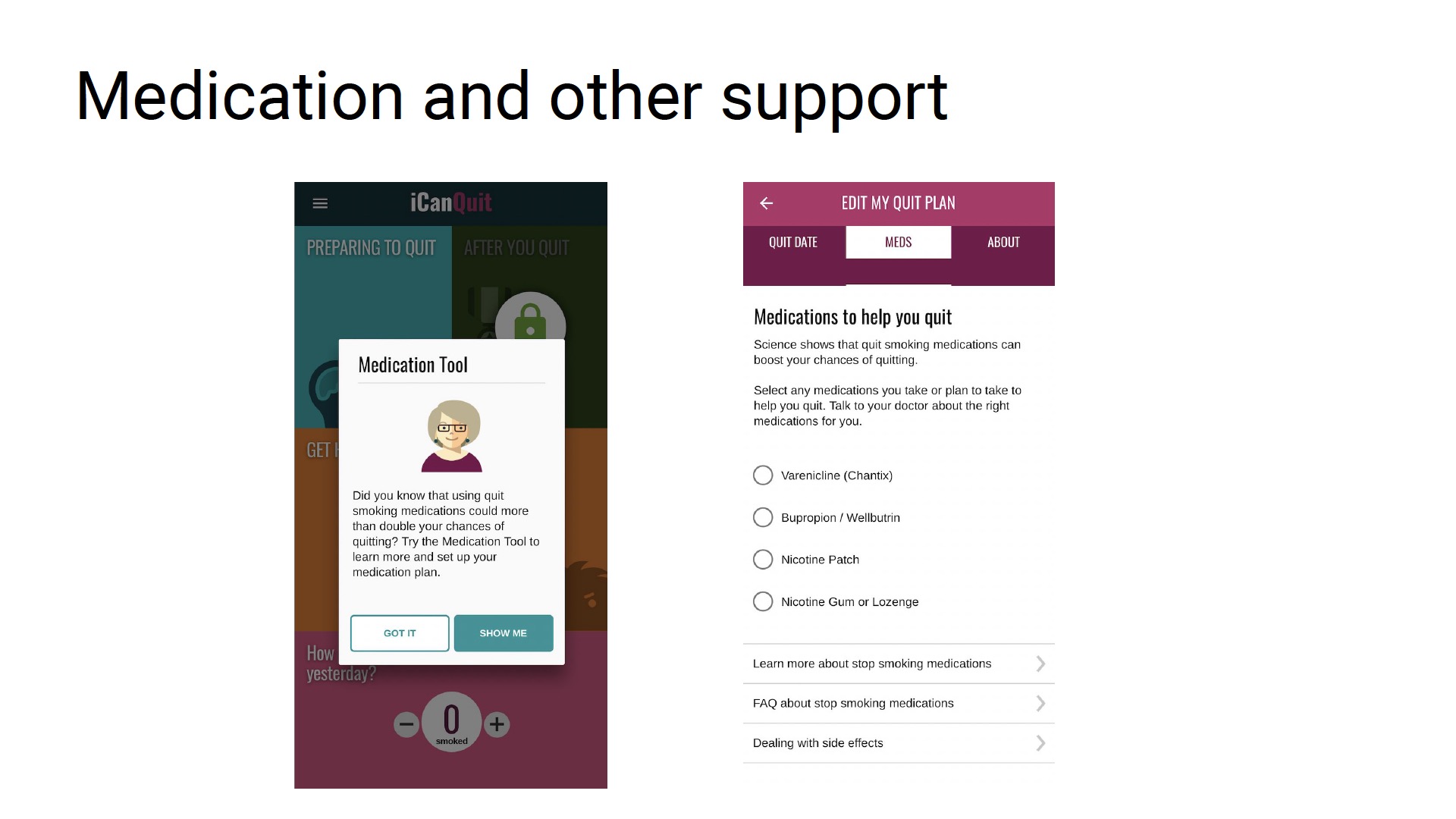

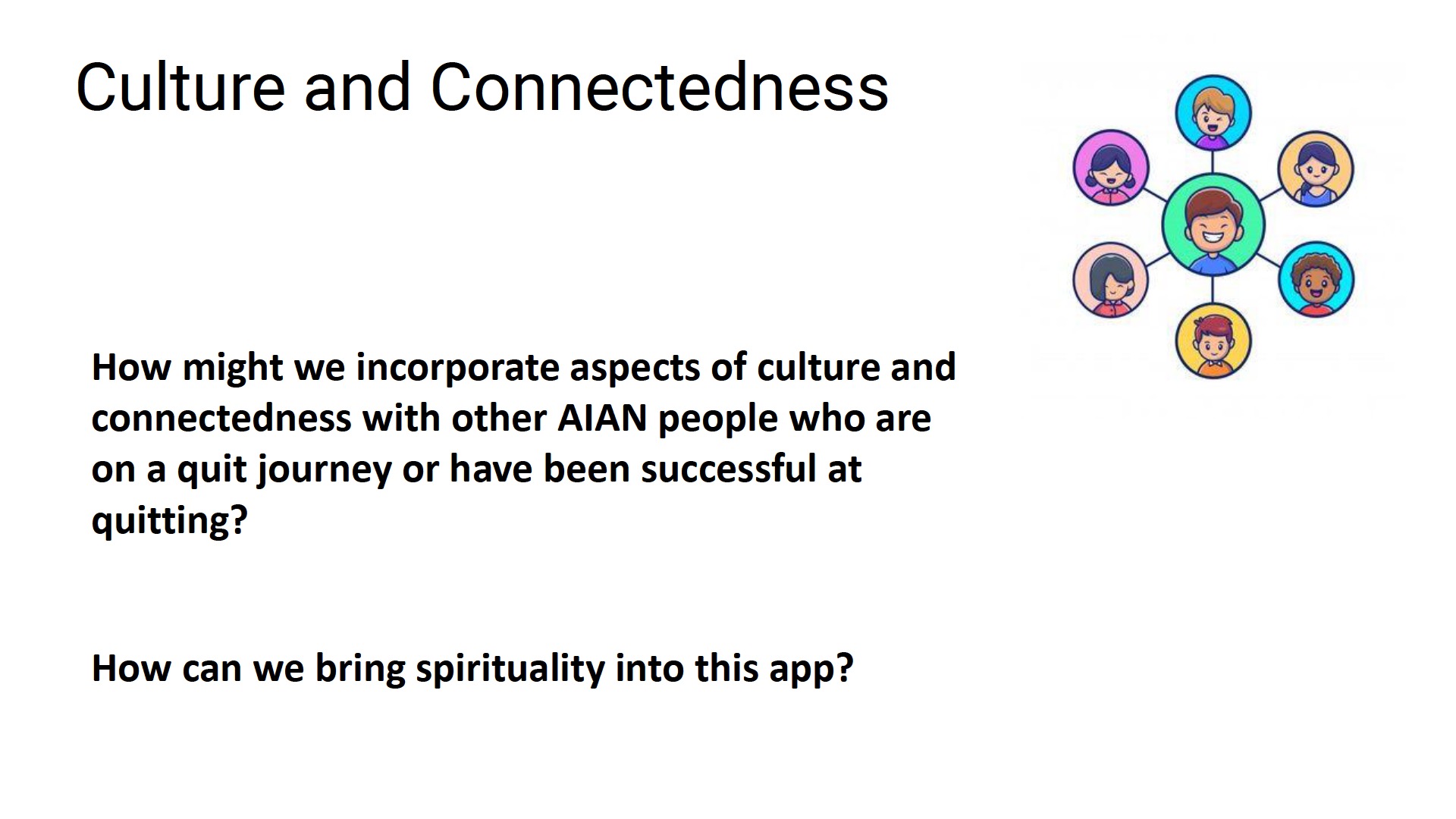

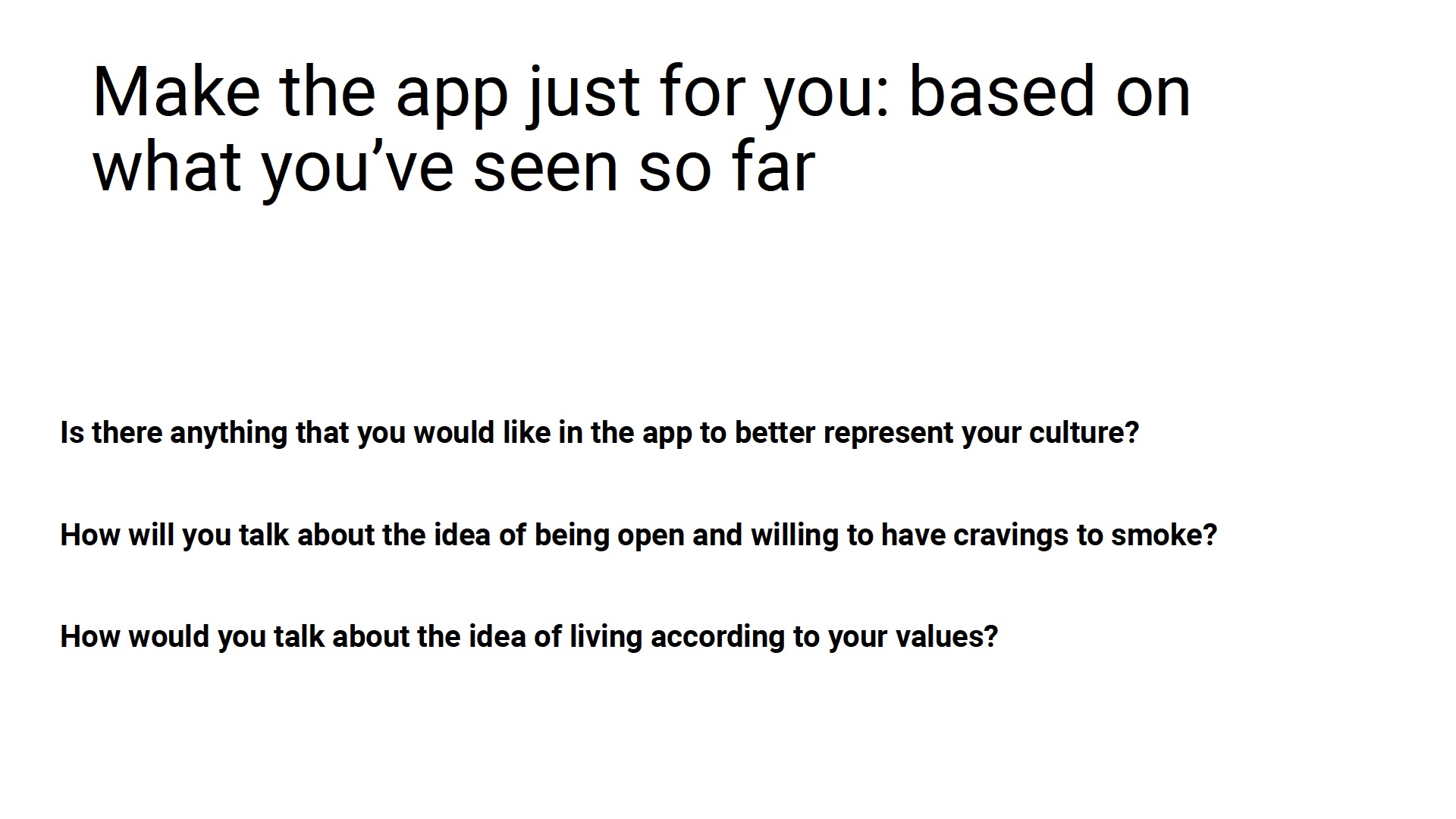

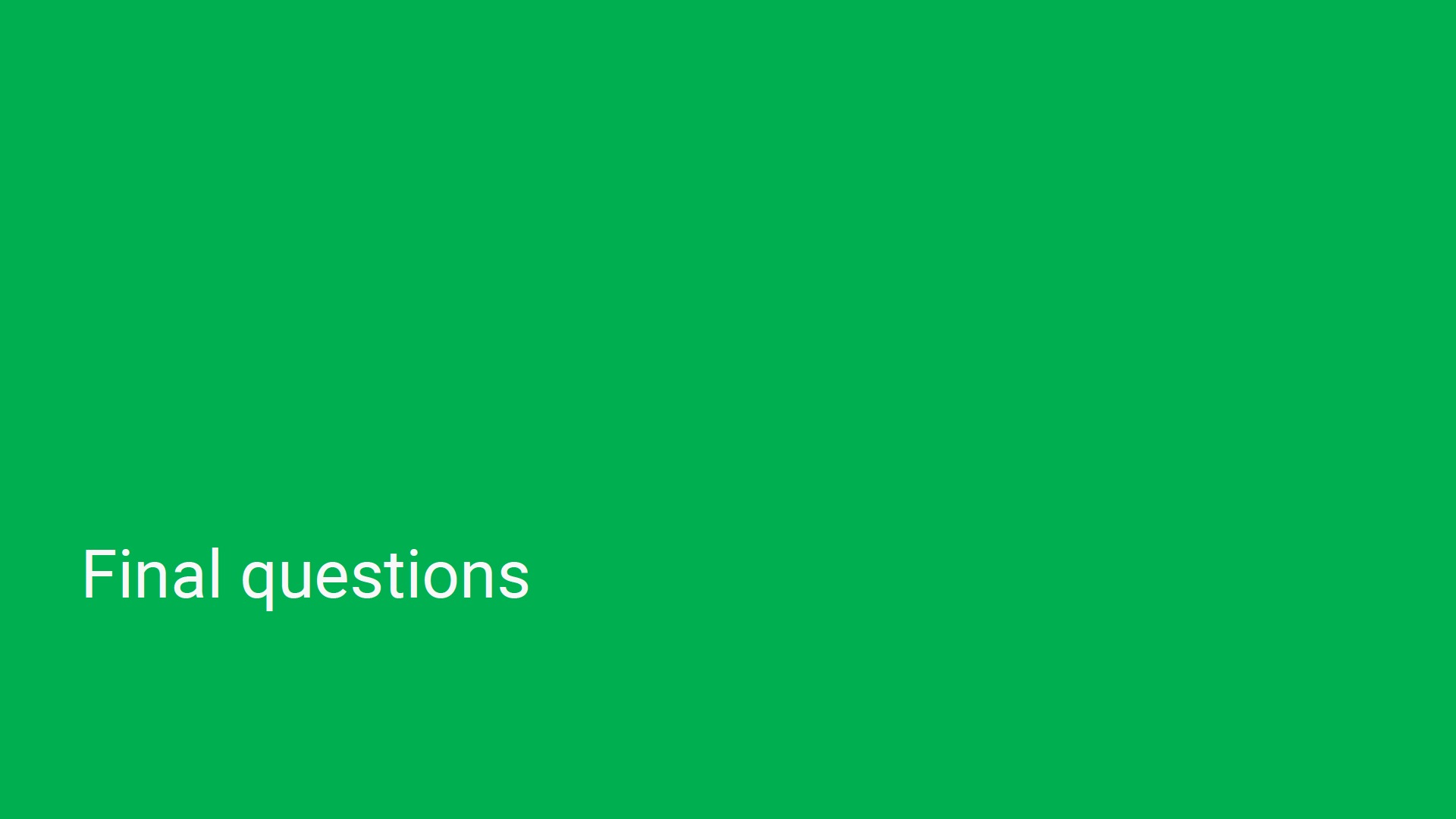

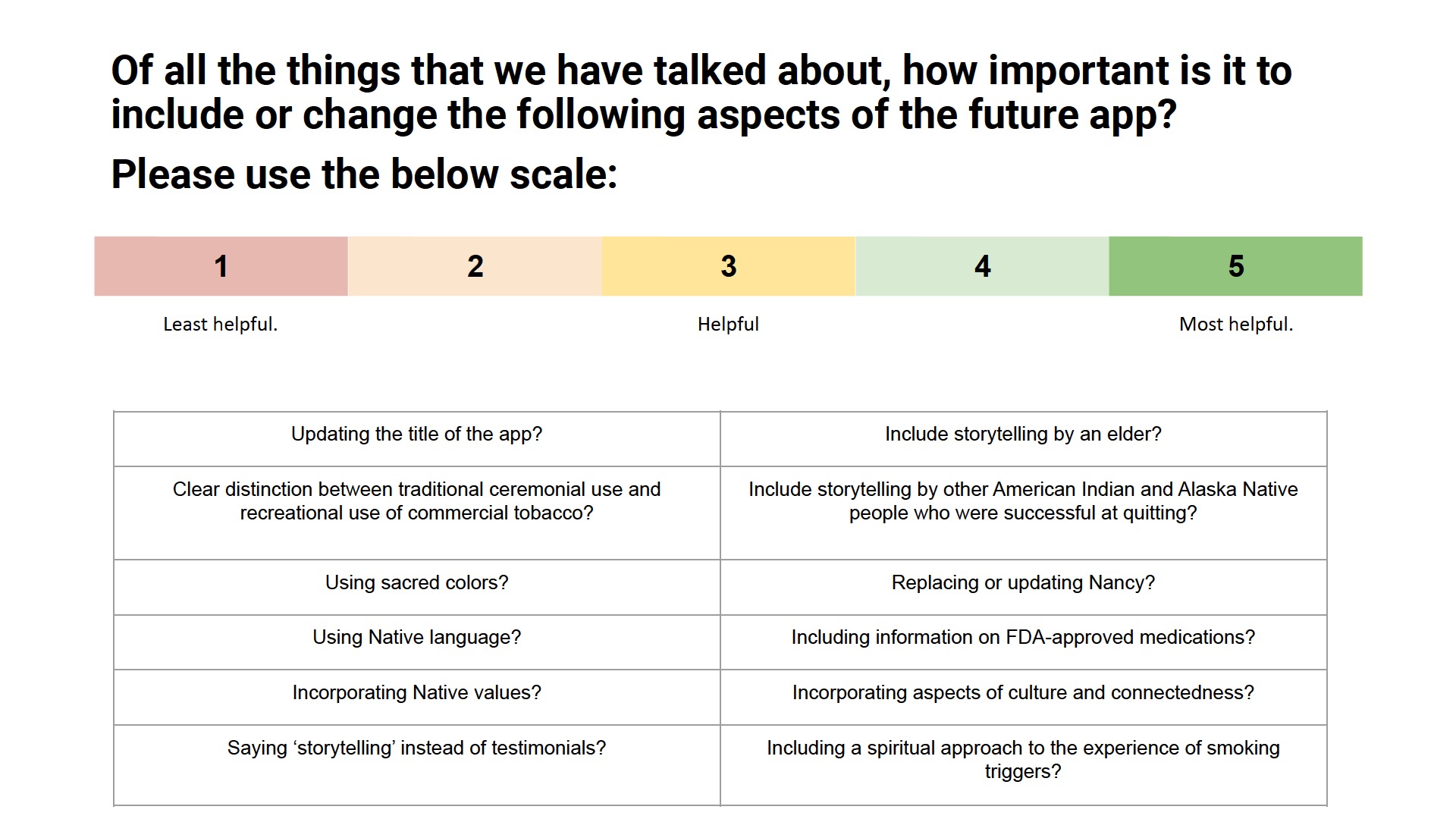

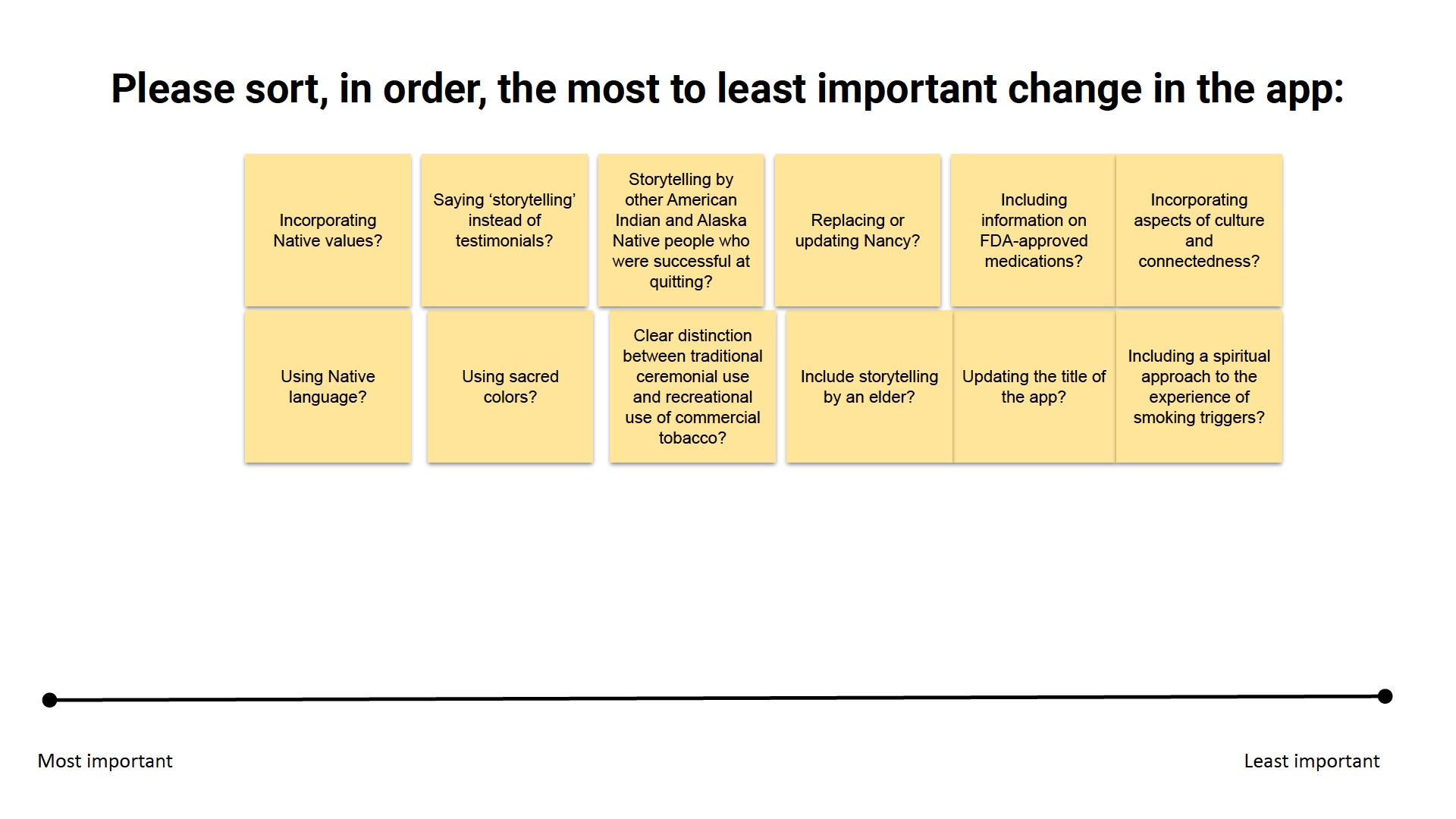

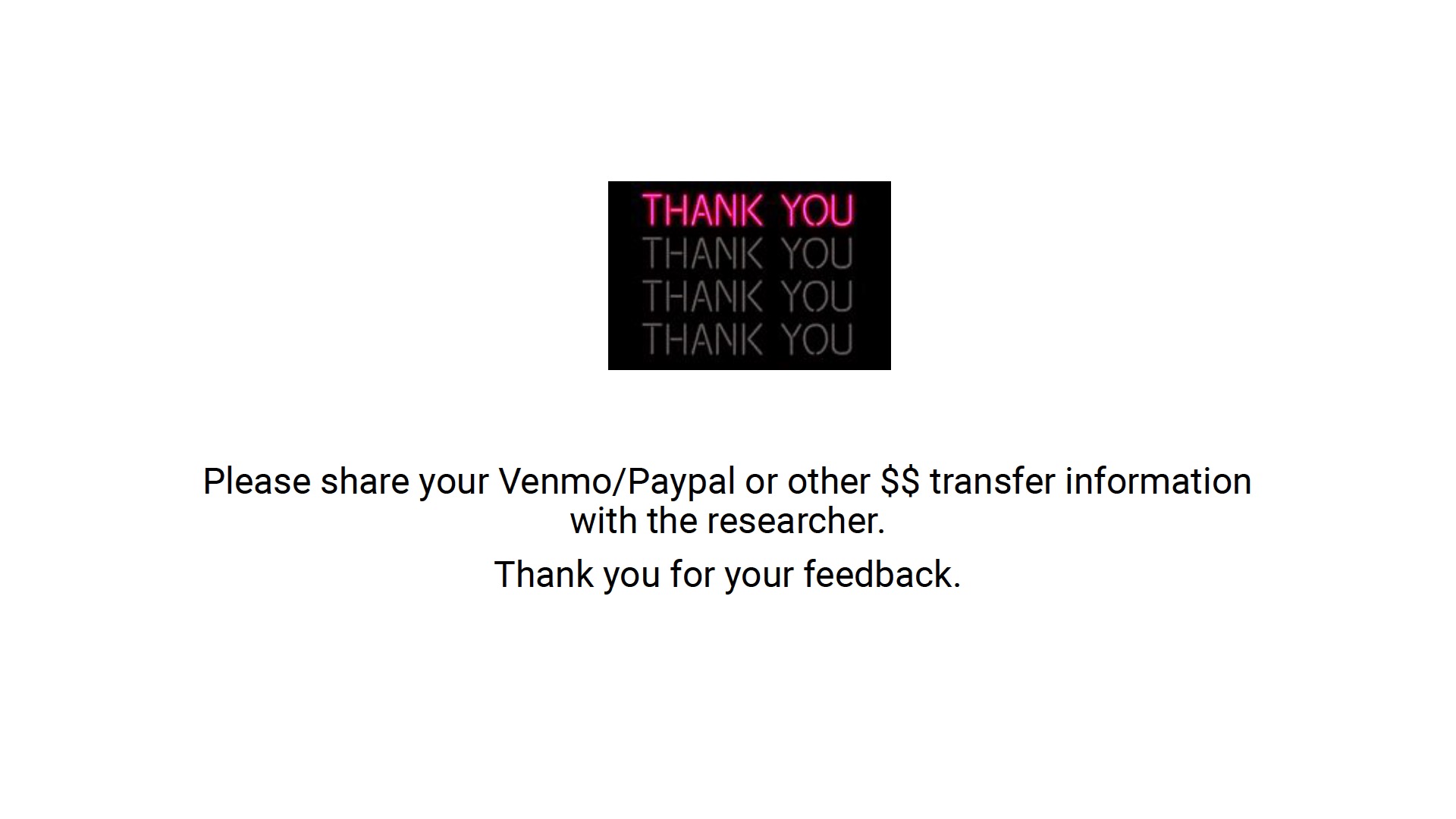
**
