# Supplementary material for: Development of a Culturally Adapted Smartphone App (IndigeQuit) Designed to Help American Indian and Alaska Native People Quit Commercial Cigarettes: User-Centered Mixed Methods Study
Source: JMIR Form Res. 2026 Mar 24;10:e88768. doi: 10.2196/88768 (PMC13058535; doi:10.2196/88768)

**Multimedia Appendix 2.** Step 2 Discussion Guide

IndigeQuit R1 User Testing Discussion (2024)

Method: 1:1 remote moderated interviews

Schedule

| TOPIC/SCENARIO | TIME ALLOTTED | CUMULATIVE TIME |
| --- | --- | --- |
| Introduction and informed consent | 5 minutes | 5 minutes |
| Warm up - tribal affiliations and culture | 5 minutes | 10 minutes |
| Background screens prototype review | 10 minutes | 20 minutes |
| Avatars prototype review | 10 minutes | 30 minutes |
| Values icons prototype review | 10 minutes | 40 minutes |
| Smoking tests feedback | 15 minutes | 55 minutes |
| Session Wrap Up | 5 minutes | 60 minutes total |

**Introduction & Consent (5 min)**

- Introduction (Moderator to explain setup and protocol, think aloud procedure)
  - Welcome to our interview about the IndigeQuit app designs and smoking tests.
  - We are so grateful that we can spend some time and understand your perceptions and experiences to help inform the final version of the app and study design, to make to the app best able to help Indigenous/Native people quit smoking, nationwide.
  - There are no right or wrong answers, and anything that you say will be kept confidential. Your experiences with your culture and how it might help us make the app better are very interesting to us. So please don’t hold back your lived experiences. This is what we’re interested in.
  - This interview will be about 60 minutes long and we have a number of designs to review, plus the feedback about the two smoking tests.
  - You are welcome to stop or take a break at any time, so please don’t hesitate to ask.
- Consent form
- Start recording

###

### **Slide 7 - Warm up (5 min)**

**Learning about the participant - Tribal affiliation, cultural identification, app experiences**

What is your tribal affiliation?

Is your residence in a reservation community? Or off reservation?

Do you participate in your tribe or Indigenous/Native traditions?

What have your experiences been with using smartphone apps to quit smoking or change behaviors (like diet or weight loss apps)?

How has cellular signal or Wi-Fi availability affected your experience using apps in these ways?

### **Slides 8-13 Feedback on the IndigeQuit app designs (up to 30 min)**

#### **Slide 8**

Spend a little time introducing the IndigeQuit app.

Prompt:

We have created these design prototypes to adjust and tailor the app for Indigenous/Native people who smoke and would like to quit. Any information that you can share that will help us make the app attractive and relevant for that purpose would be greatly appreciated.

Any thoughts, feelings, stories, or anything that comes from your experience is exactly what we are looking for. There are no right or wrong answers. Try to imagine these designs and the entire app as if you built it for someone like you.

#### **Slide 9 (Up to 10 min)**

Please take a look at the home screen, and then at the examples of pages of the app.

Possible question prompts:

Does this feel like this was made for you?

If so, what do you like about it?

If not, what can be changed or adjusted so you feel that it is made for you?

Is there anything that should or can be included that would make this more relevant to you?

#### **Slide 10 Feedback on Guide (Up to 10 min total for Slides 10-12)**

This is the image of the main avatar, who will guide people through the IndigeQuit program. We’d like to know your impressions about her, and what you think would be a good name for her.

Possible question prompts:

What are the things that work for you about this design? What things don’t work or could be improved?

What names would you suggest for her (can also go to next slide for options)?

Would knowing her tribal or clan affiliation matter? And to what extent would it matter?

#### **Slide 11 Feedback on female testimonials/stories avatars**

Prompt: In the app, we will share stories about Indigenous/Native people who have successfully quit smoking using IndigeQuit, and how it worked for them. Please take a look at the designs for the people in those stories here.

Possible question prompts:

How do you feel about these images?

How do you feel about these possible names?

Are there any particular elements you’d like to see in these stories? What’s going to make these stories as real and helpful for you as possible?

#### **Slide 12 – Feedback on male testimonials/stories avatars**

Possible question prompts:

How do you feel about these images?

How do you feel about these possible names?

Are there any particular elements you’d like to see in these stories? What’s going to make these stories as real and helpful for you as possible?

#### **Slide 13 – Feedback on Values icons (Up to 10 min)**

In the IndigeQuit app, people will be identifying their three most important values as their motivation for quitting smoking. These icons will represent the important reasons people can choose for their main motivators: Family, Health, etc. We have also added an icon to represent Caring for the Environment.

What are your thoughts about each of these?

### **Slide 15 Feedback on Smoking Tests (up to 15 min)**

Prompt: did you receive the smoking tests that we mailed you? You should have gotten two tests: a saliva test, and a breathalyzer, plus instructions on how to use both of them. For the breathalyzer to work, you would also have had to install the coVita app.

Possible prompt questions:

Were you able to do the tests before this interview?

For those who say NO, they didn’t do the tests:

- What was the problem? Why didn’t this work for you?
- Did you have any concerns about using the tests? What were they? Anything about the device, or how we’ll use the data?
- How could we troubleshoot or help people who have this problem in the future?
- How could the instructions be improved to make testing as easy as possible? (Would a video have helped?)
- Is there anything we could do to increase the chances that people will do the tests?

Also see if they are willing to do the tests in the interview, and give real time feedback about their experience.

For those who say YES, they did the tests:

- What problems did you have in trying to make the smoking test work so you could get a clear result (saliva test or smokelyzer) that you were sure was accurate?
- If you encountered any problems or trouble, what was it? How could we troubleshoot or help people who have this problem in the future?
- What made it work for you to do the test(s)?
- How comfortable were you?
- Did you have any concerns about using the tests?

Test comparison

- Which test do you prefer, and why?
- Which do you think people would be most likely to use?
- Is there anything we could do to increase the chances that people will do the test?
- How could the instructions be improved to make testing as easy as possible? (Would a video have helped?)

### **Slide 17-18 Closing (up to 5 min)**

#### **Slide 17 Final open-ended questions (Up to 5 min)**

Possible Question Prompts:

What else would you like to see in the app to better represent your culture?

What would you like to add that I haven’t asked about yet?

Is there anything in this app that you find uncomfortable?

**Slide 18 Participant Gratuity and Thank you.**

Confirm address and payment details.


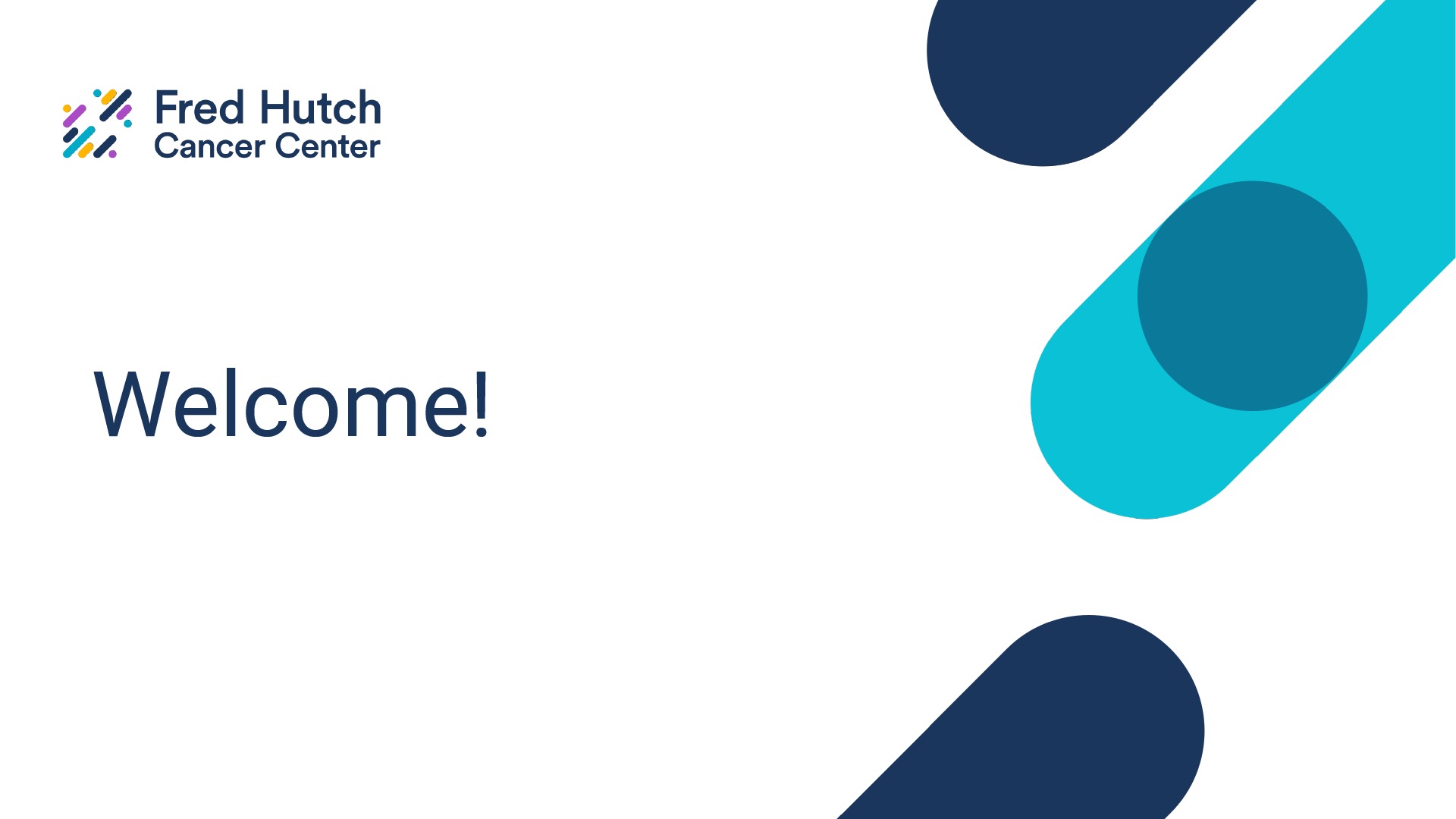

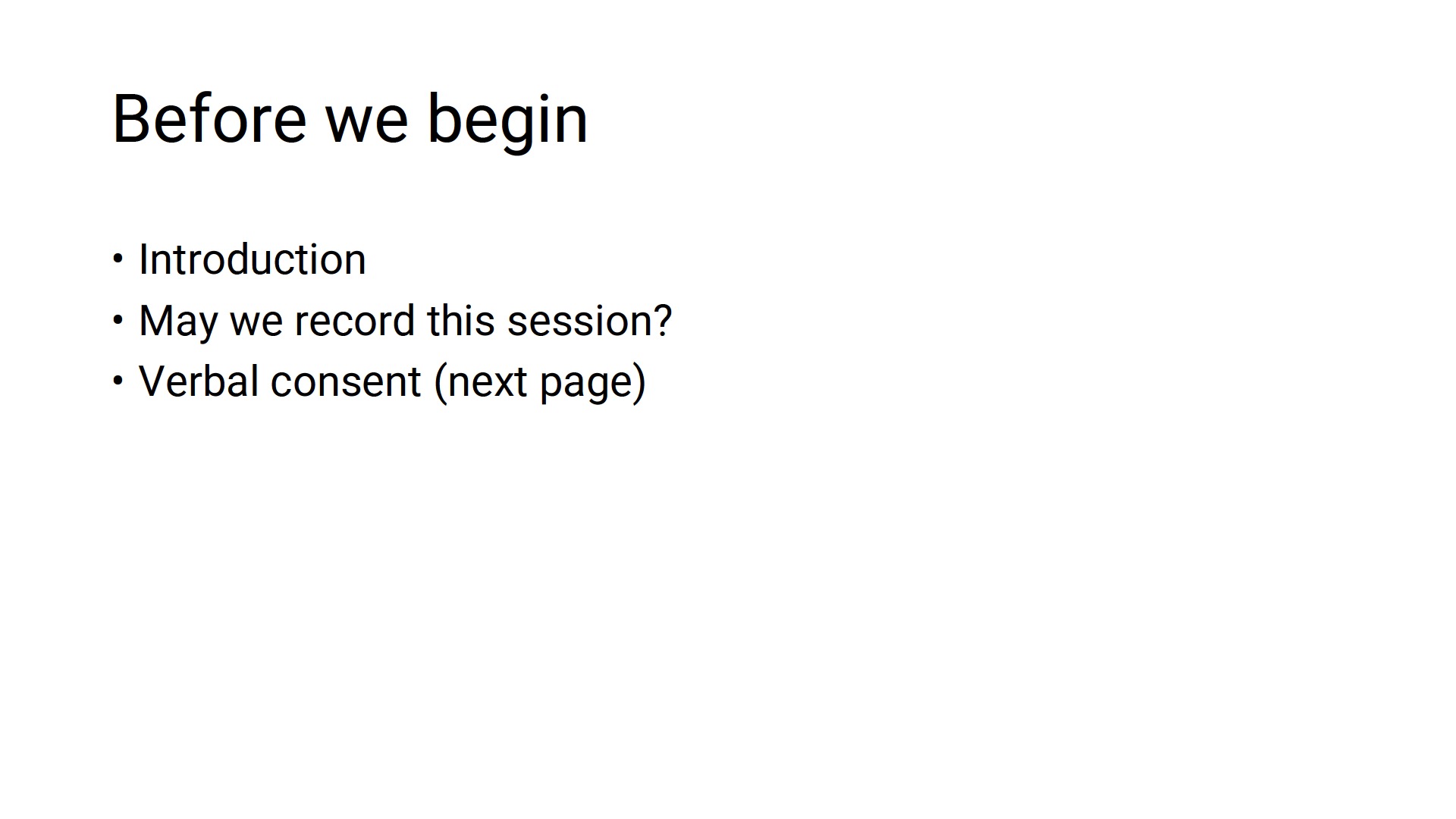

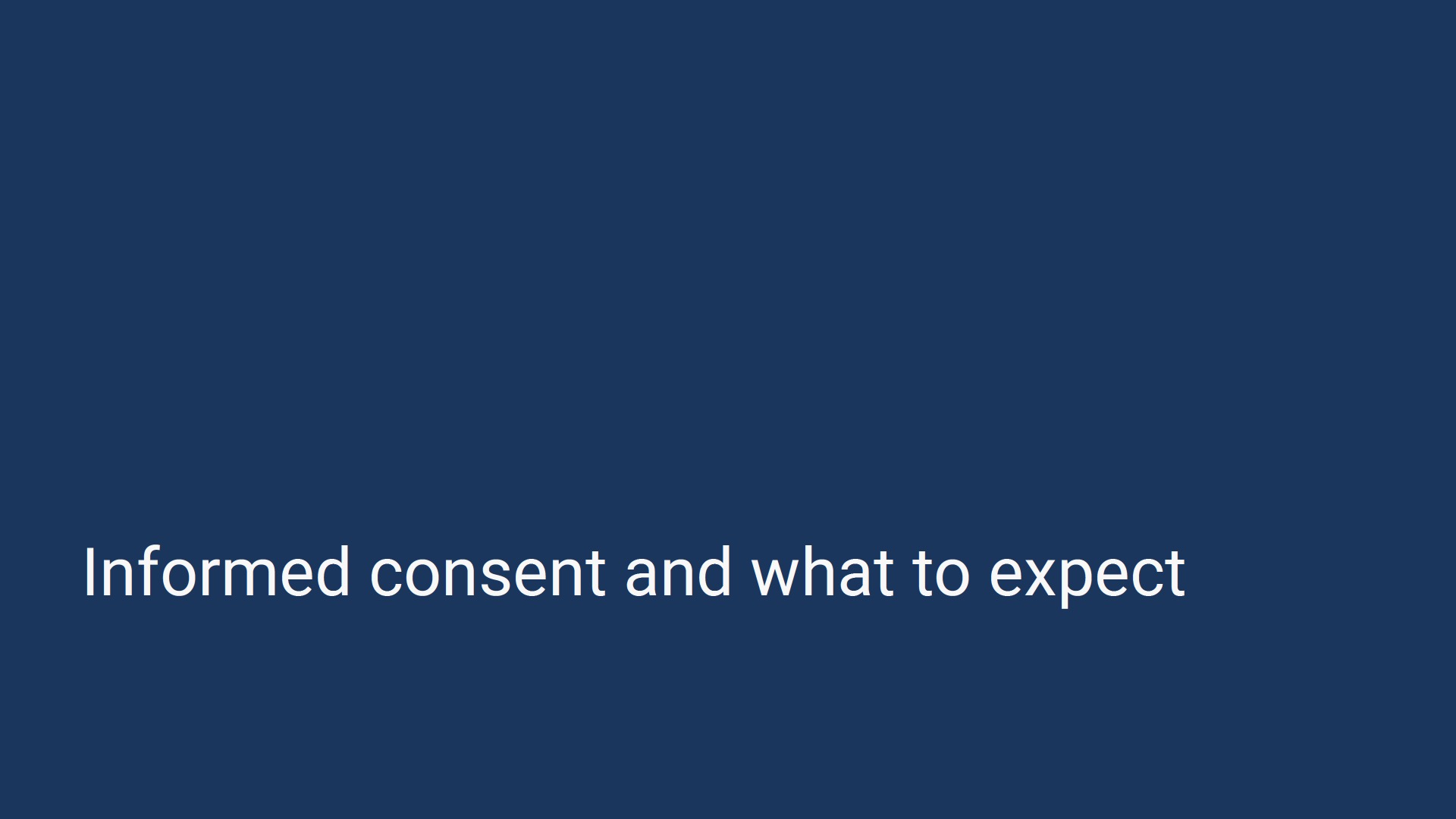

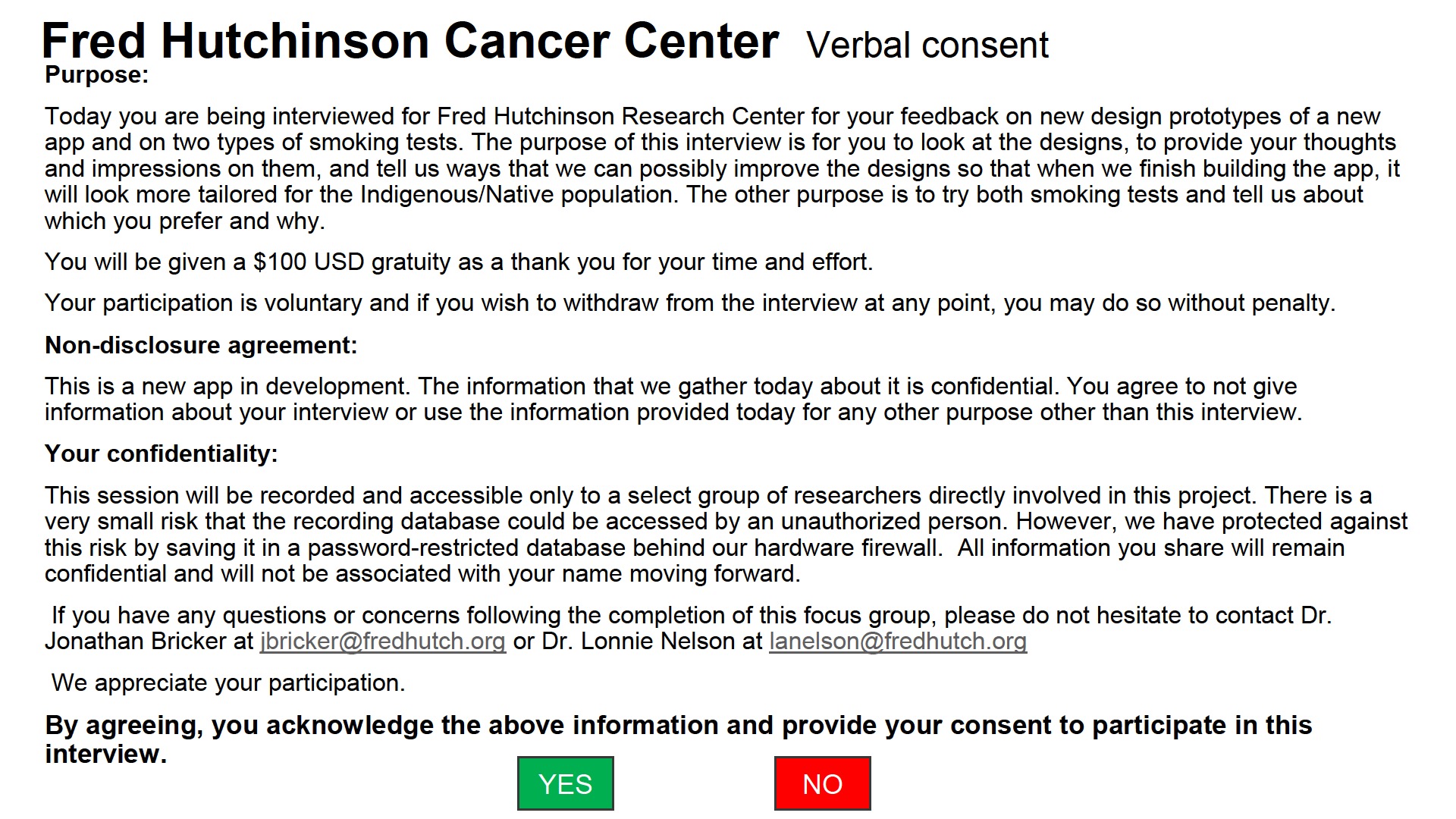

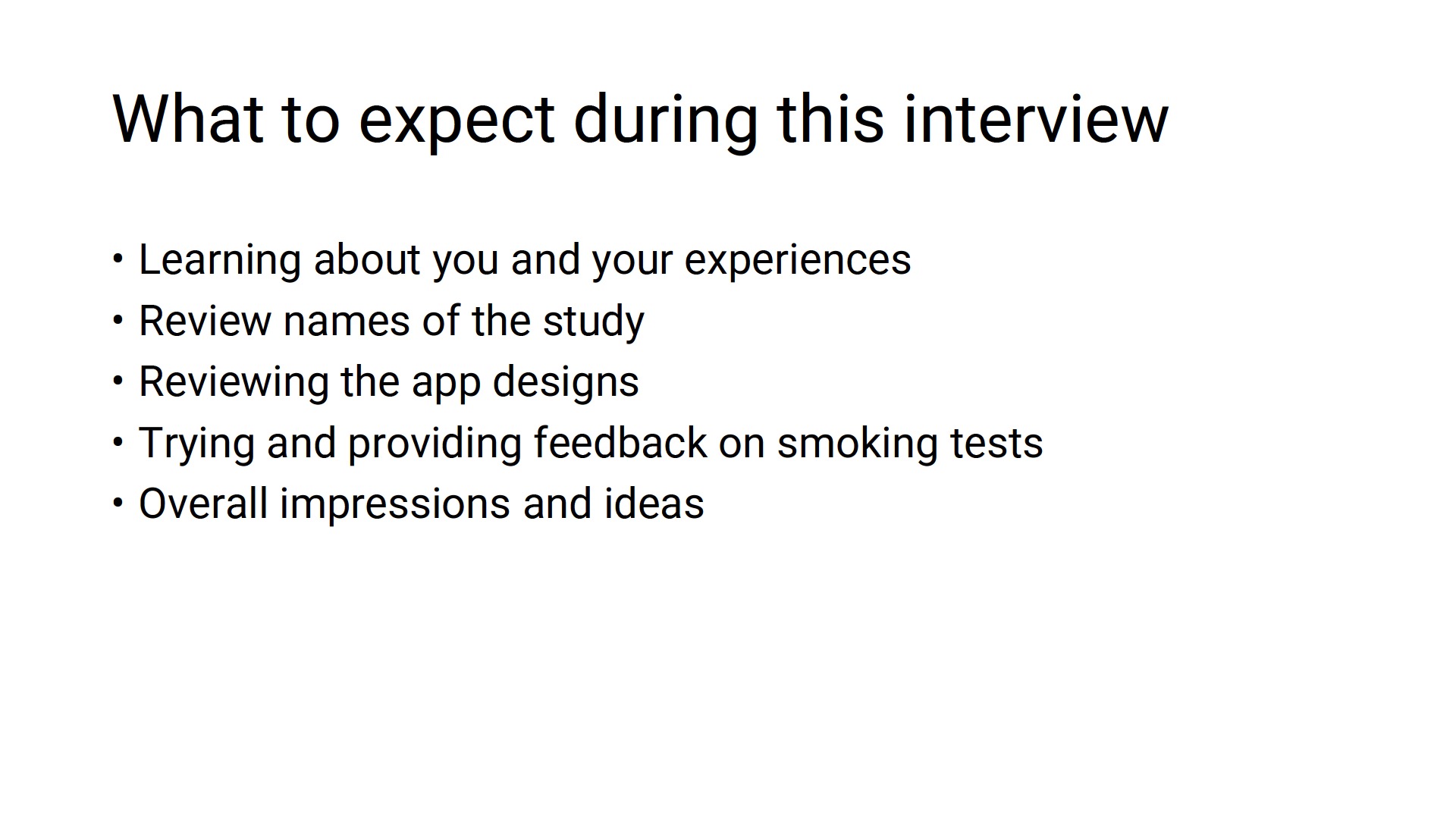

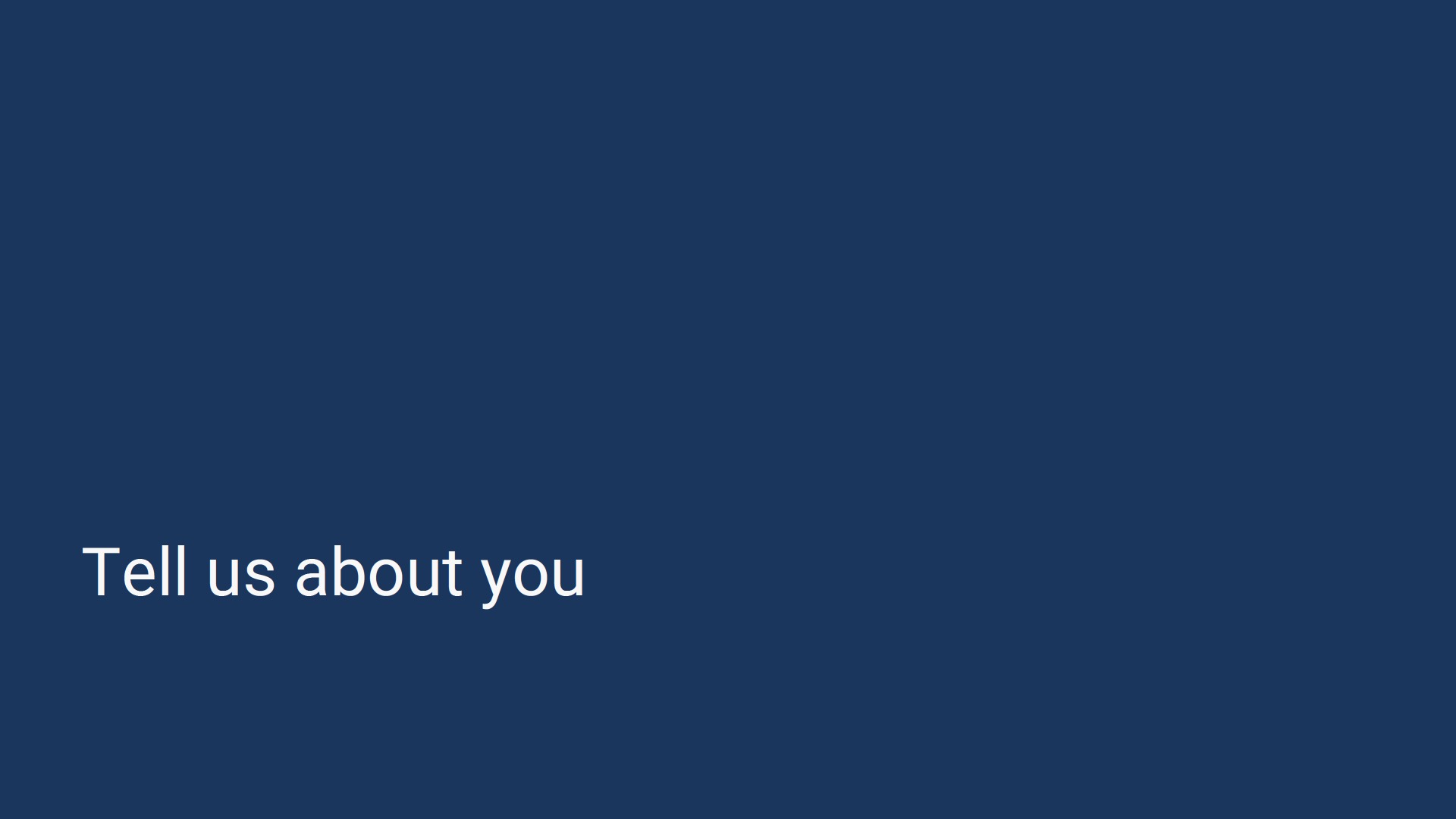

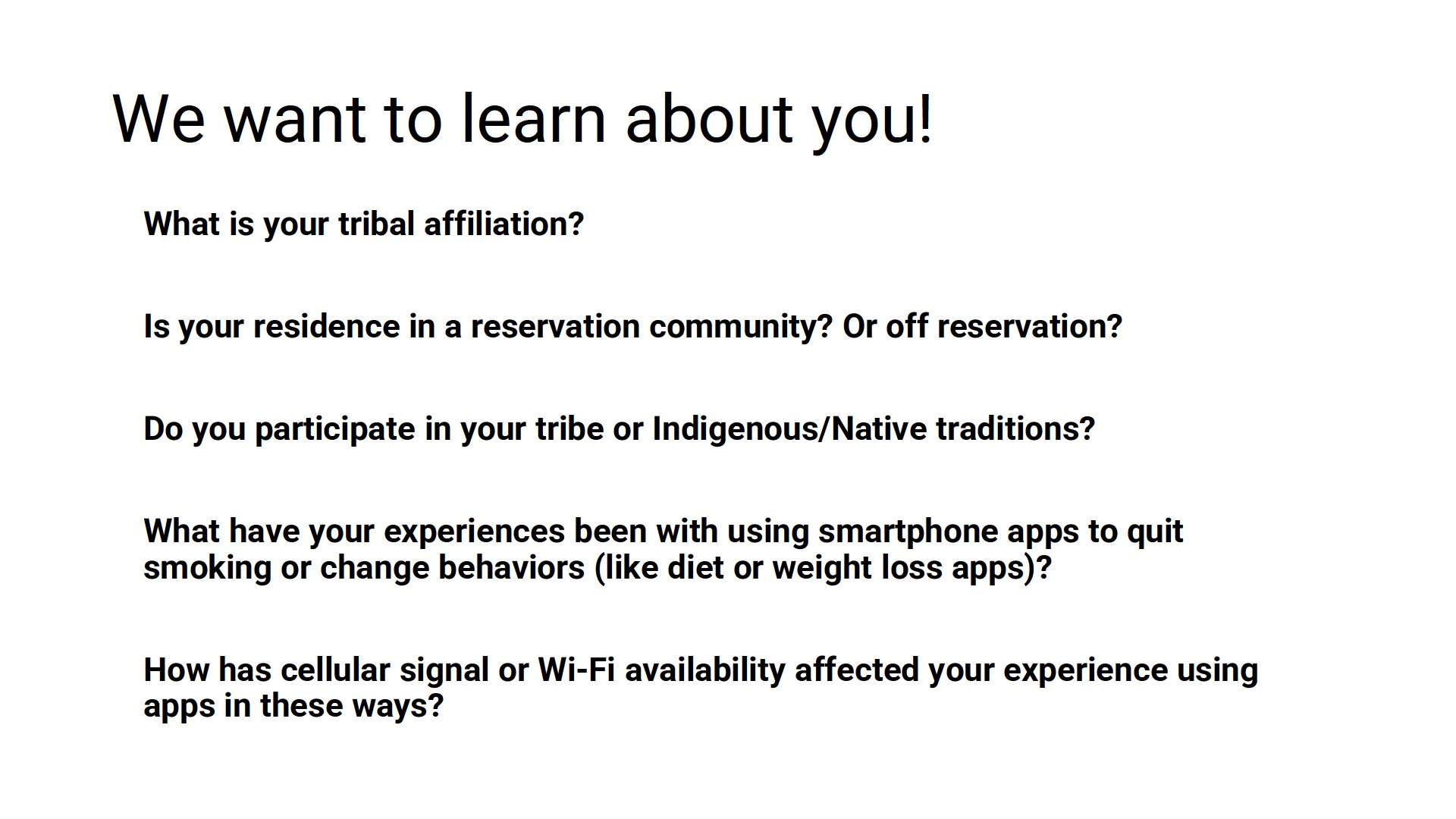

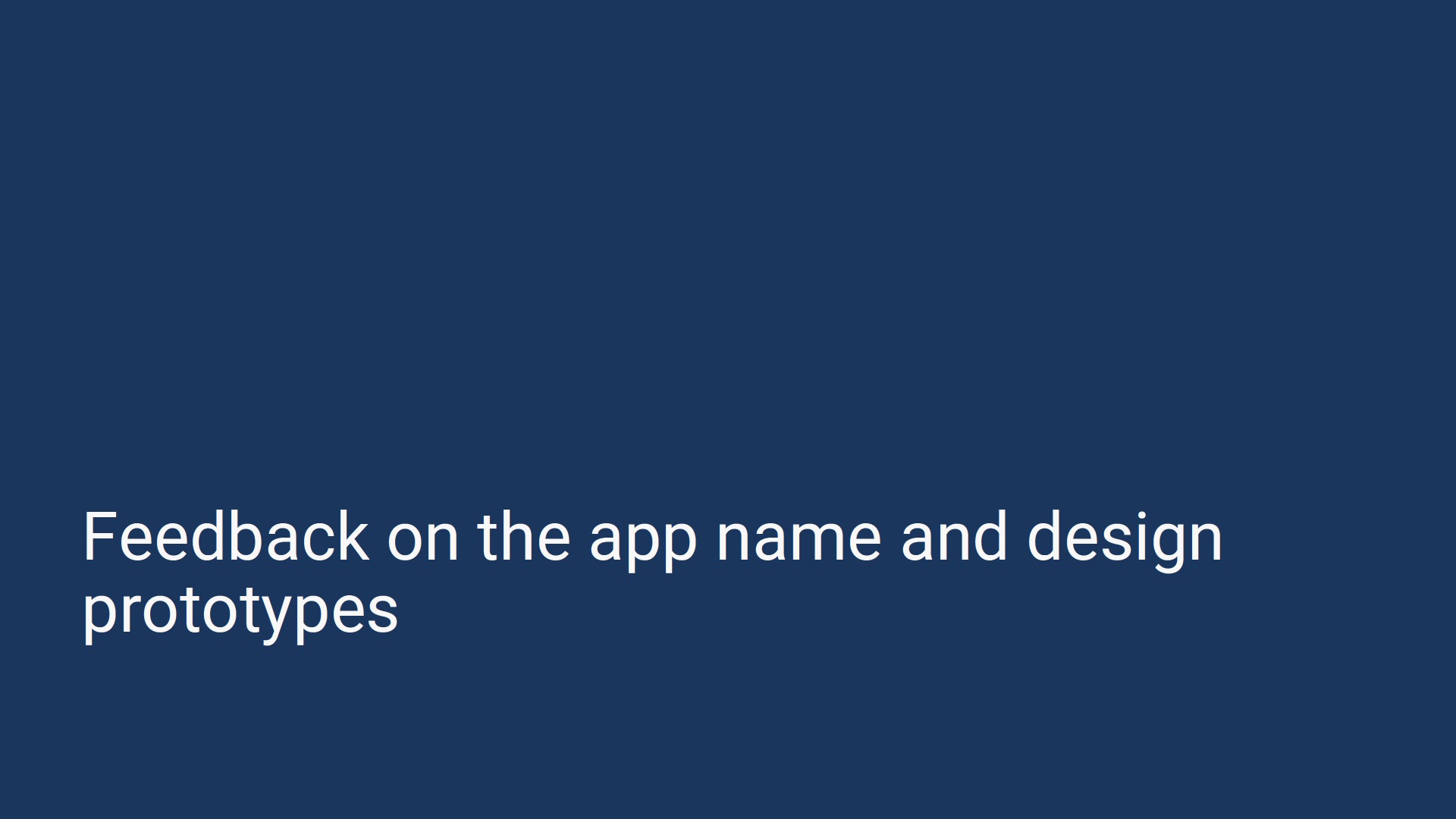

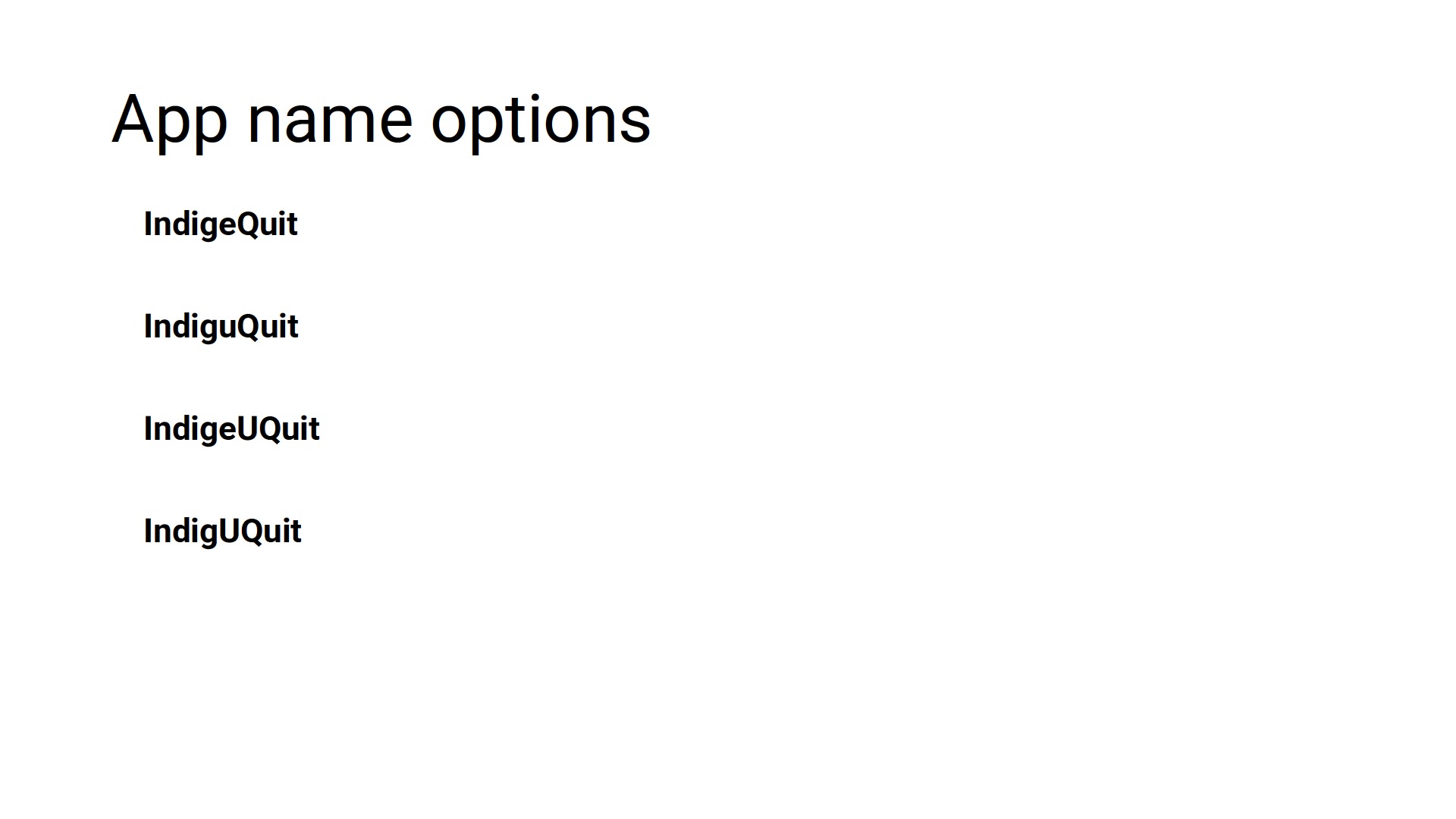

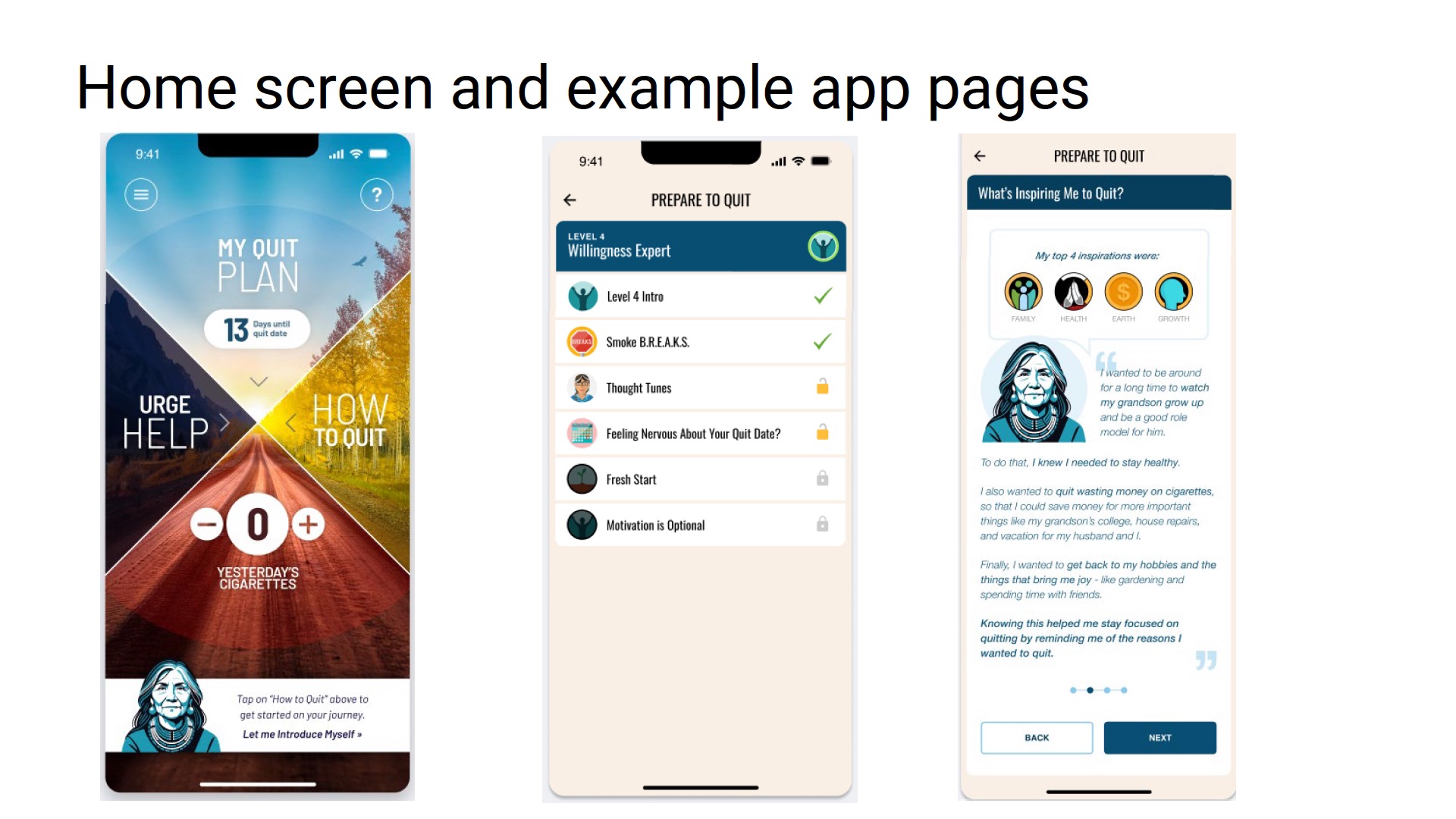

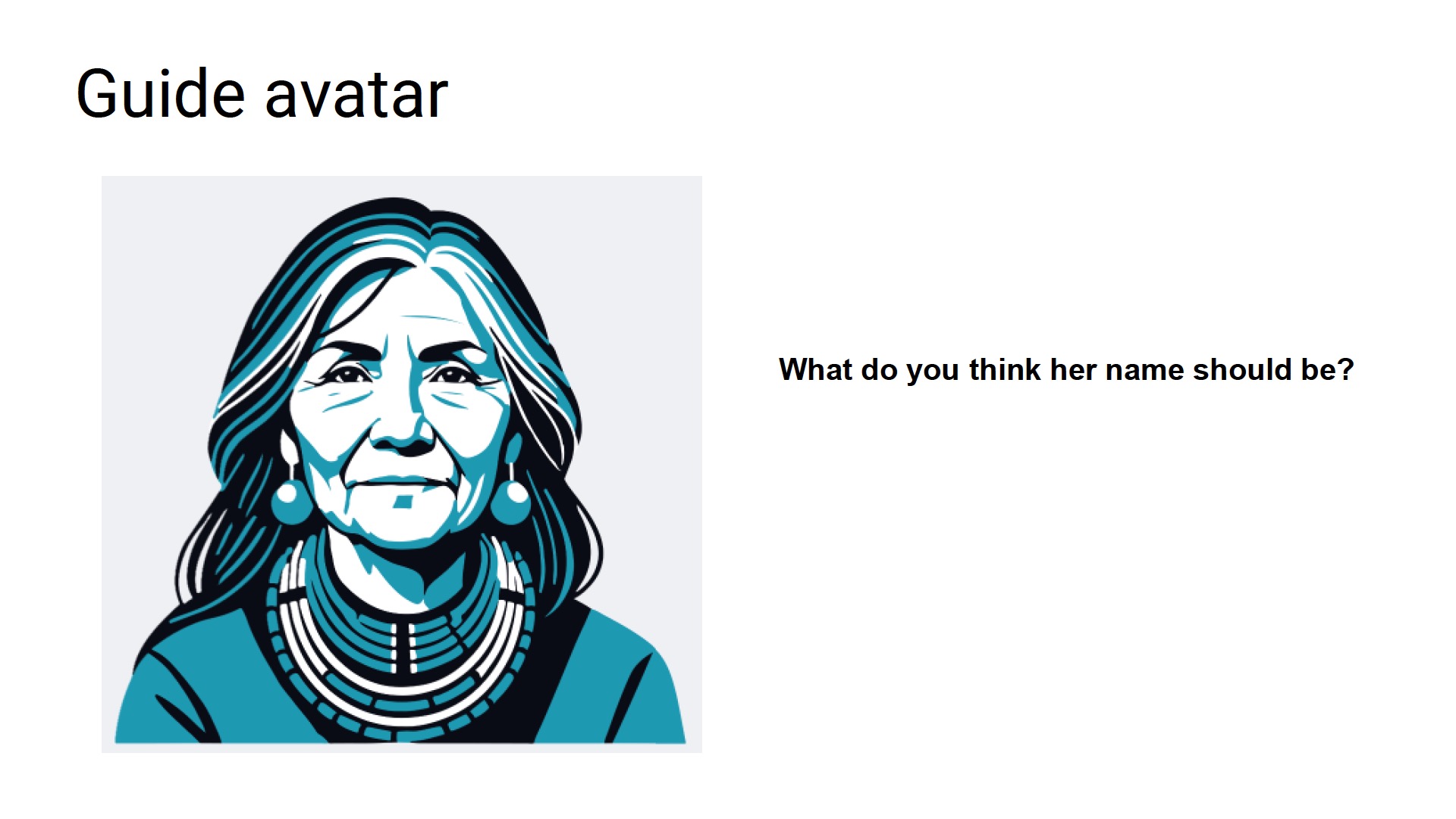

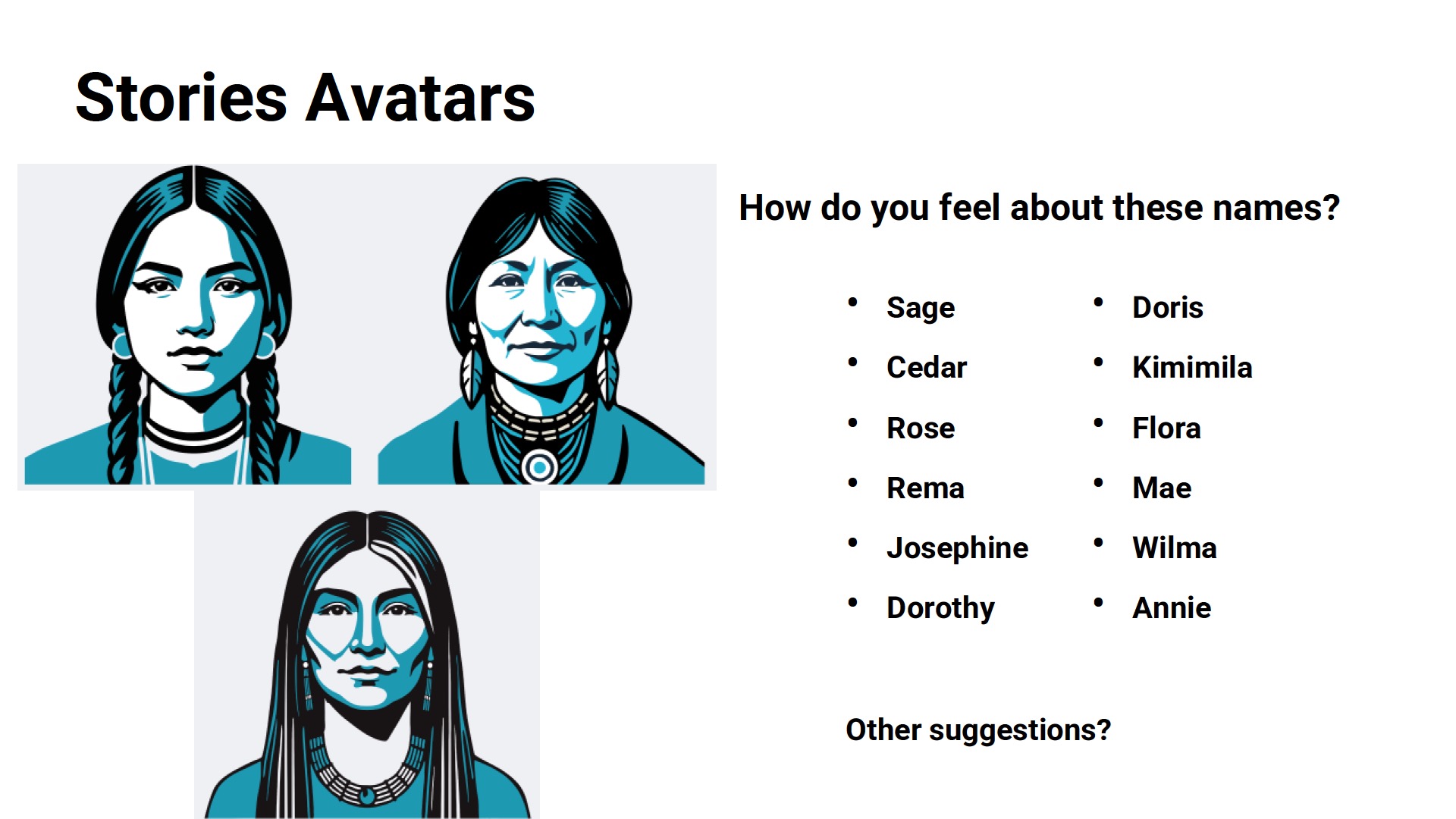

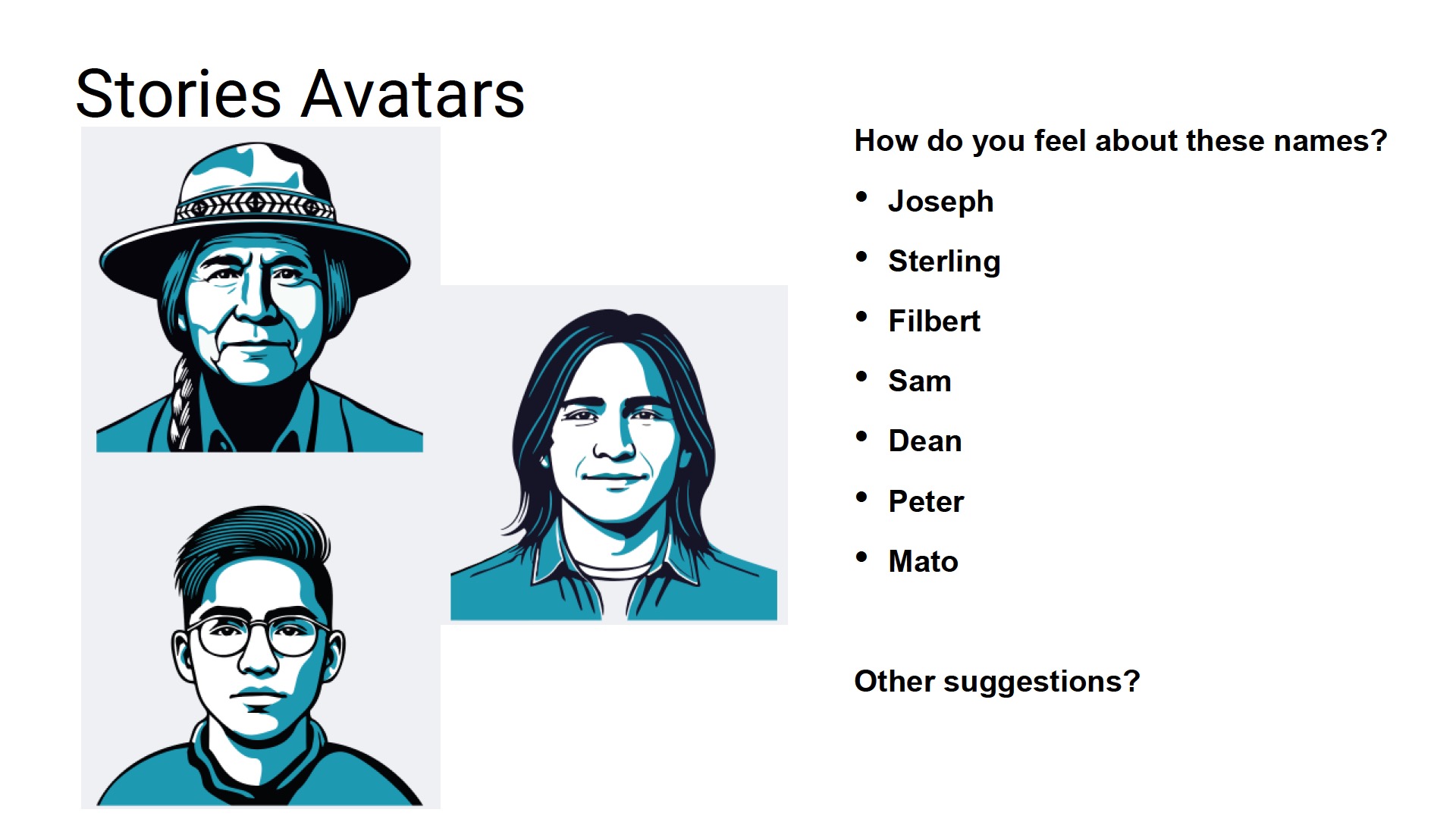

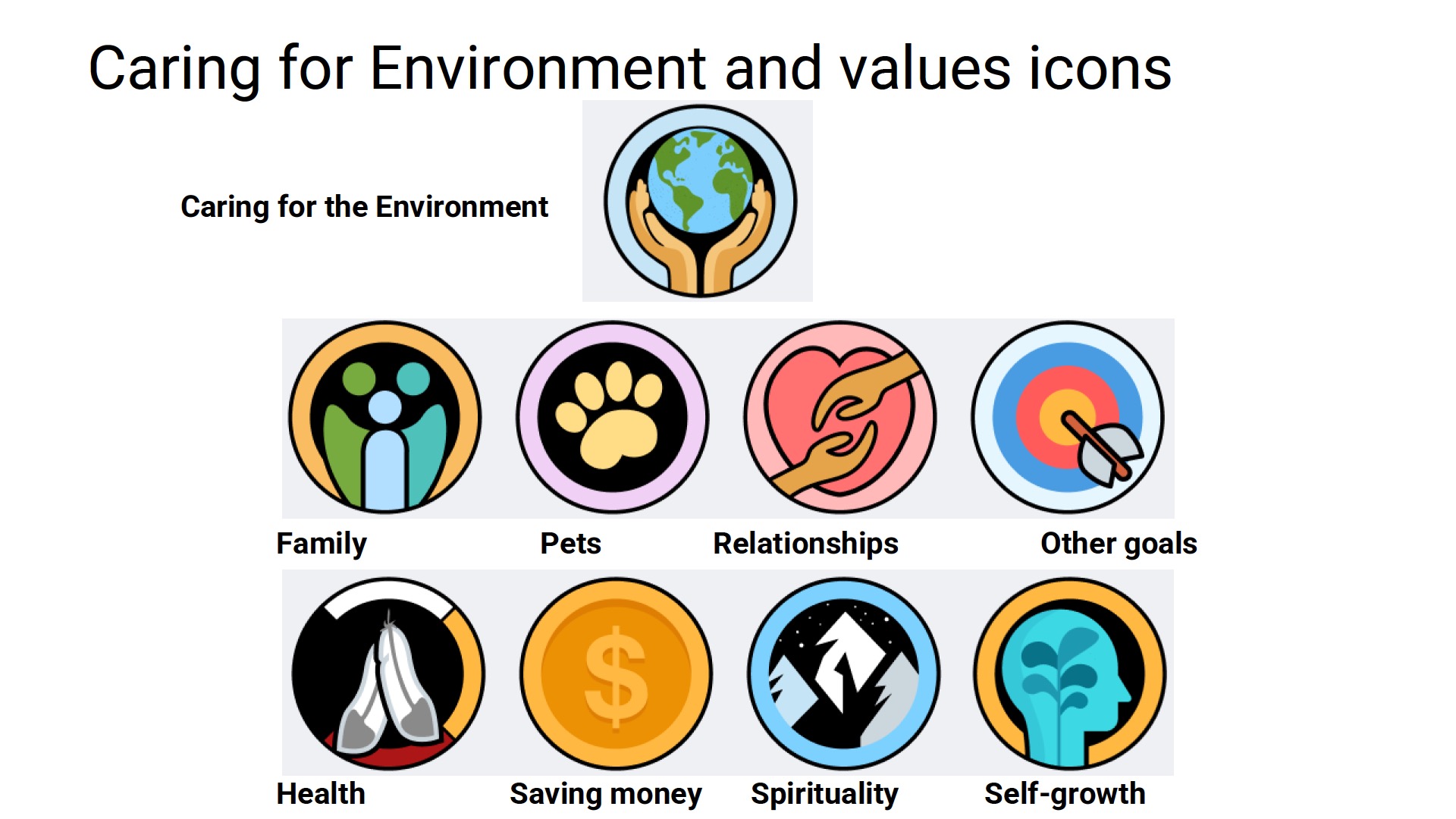

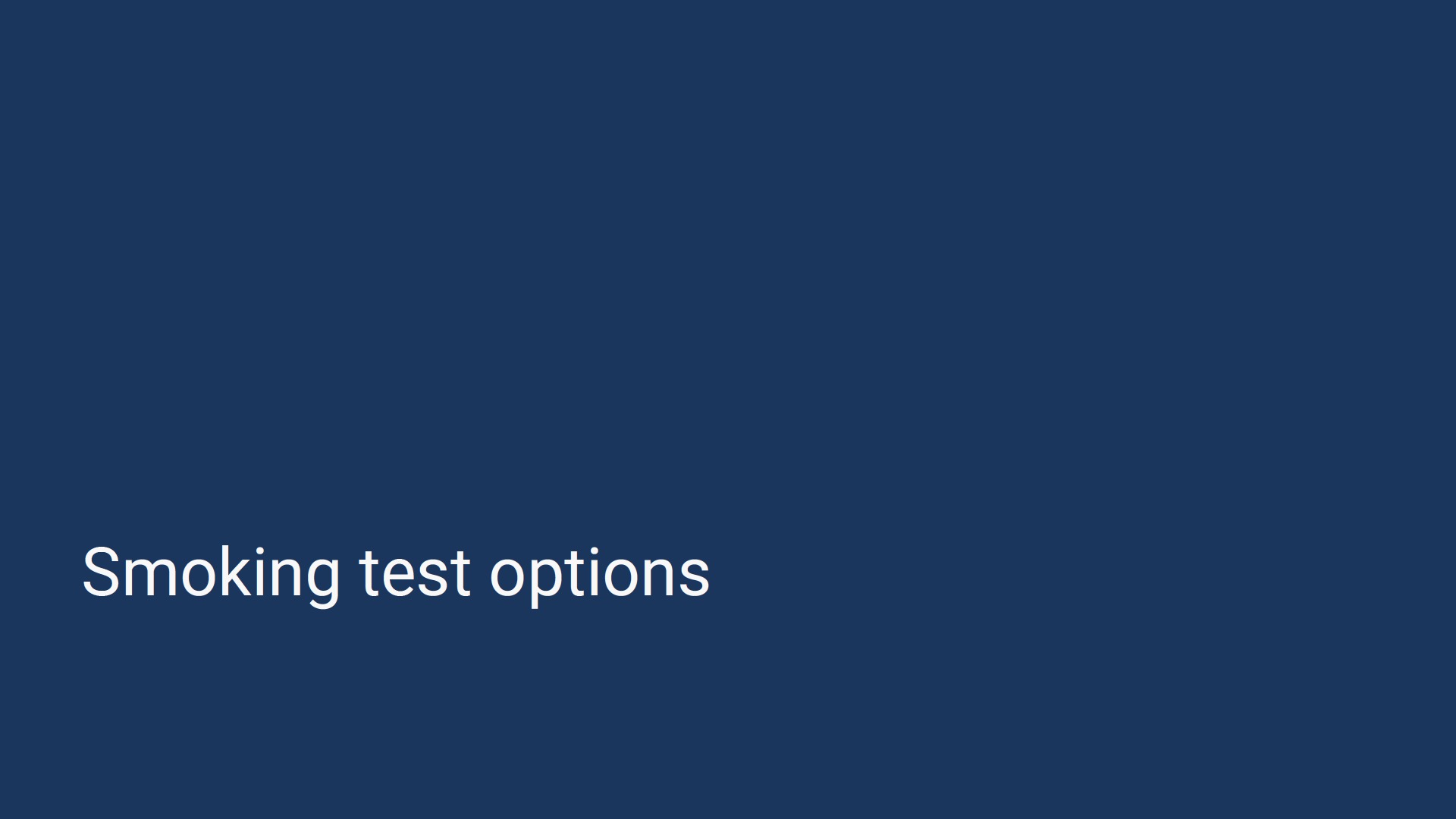

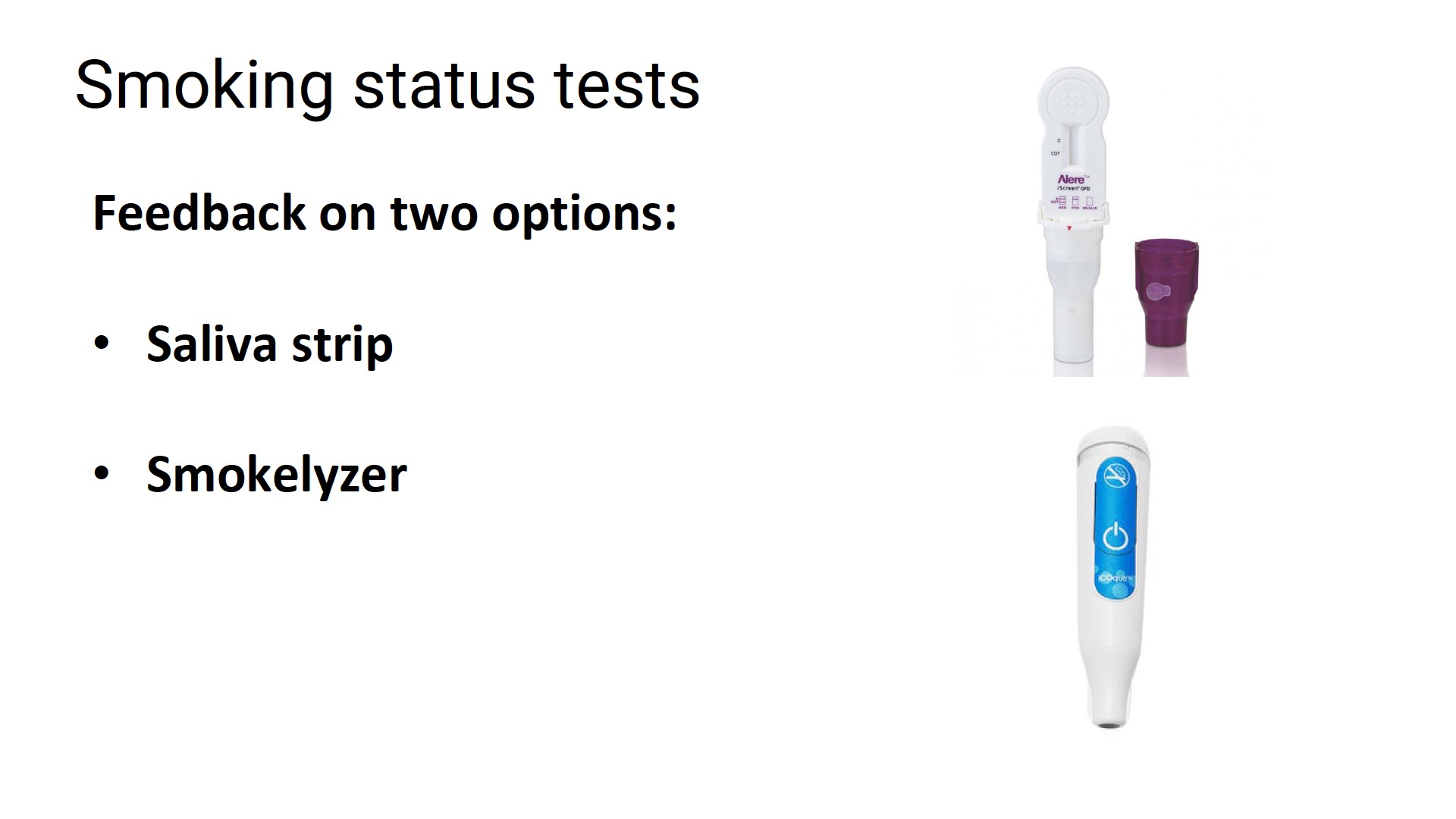

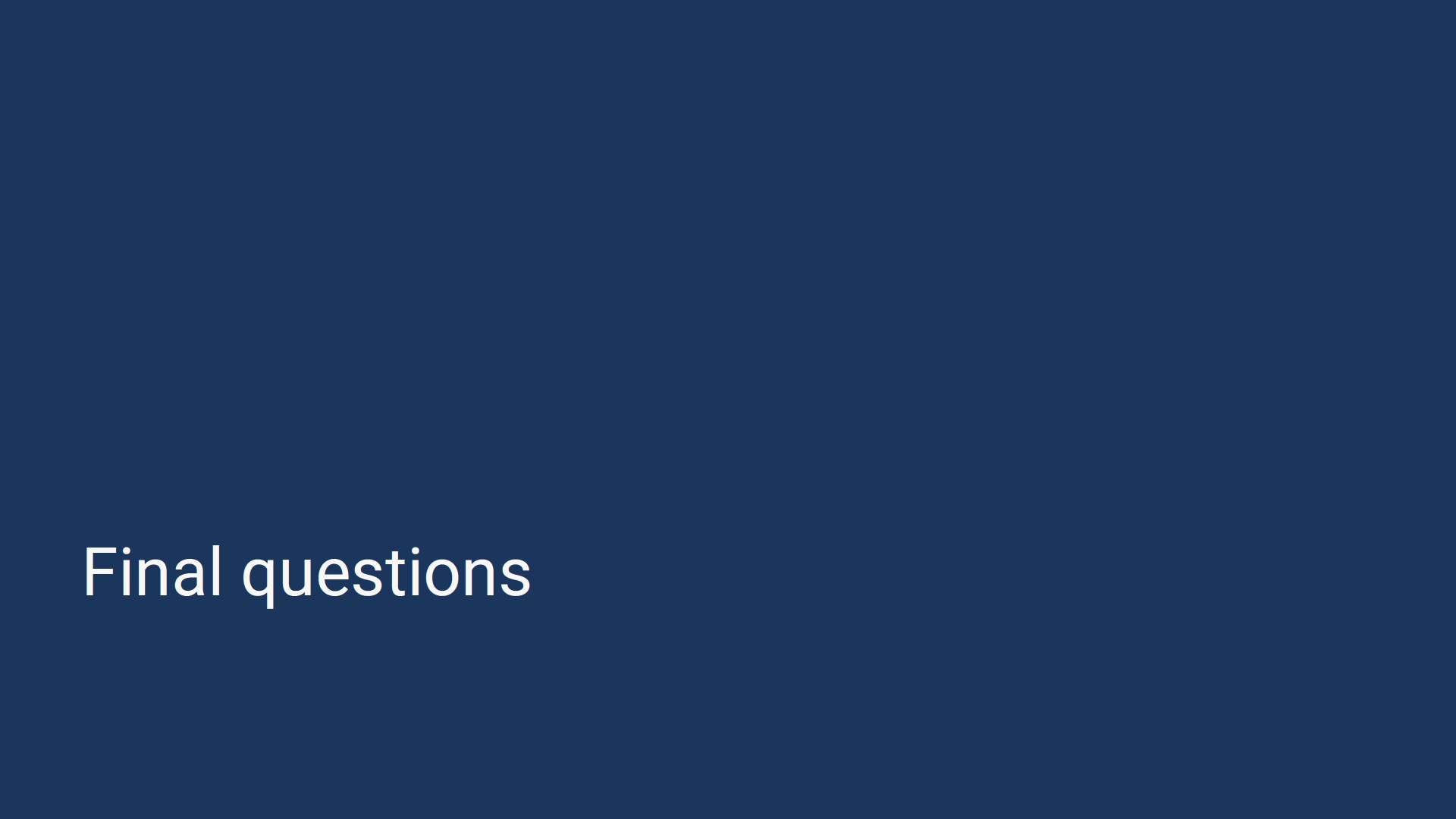

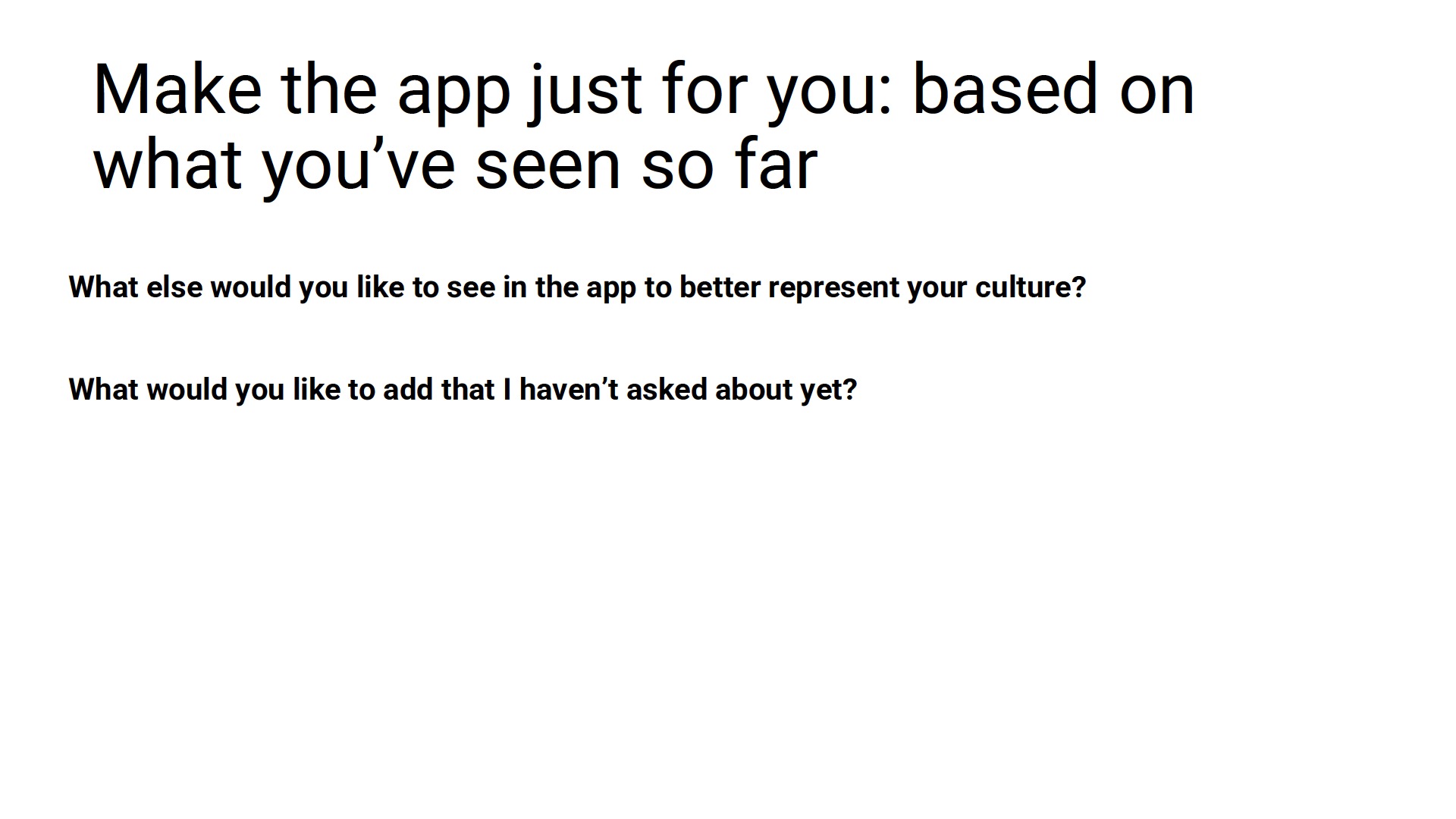

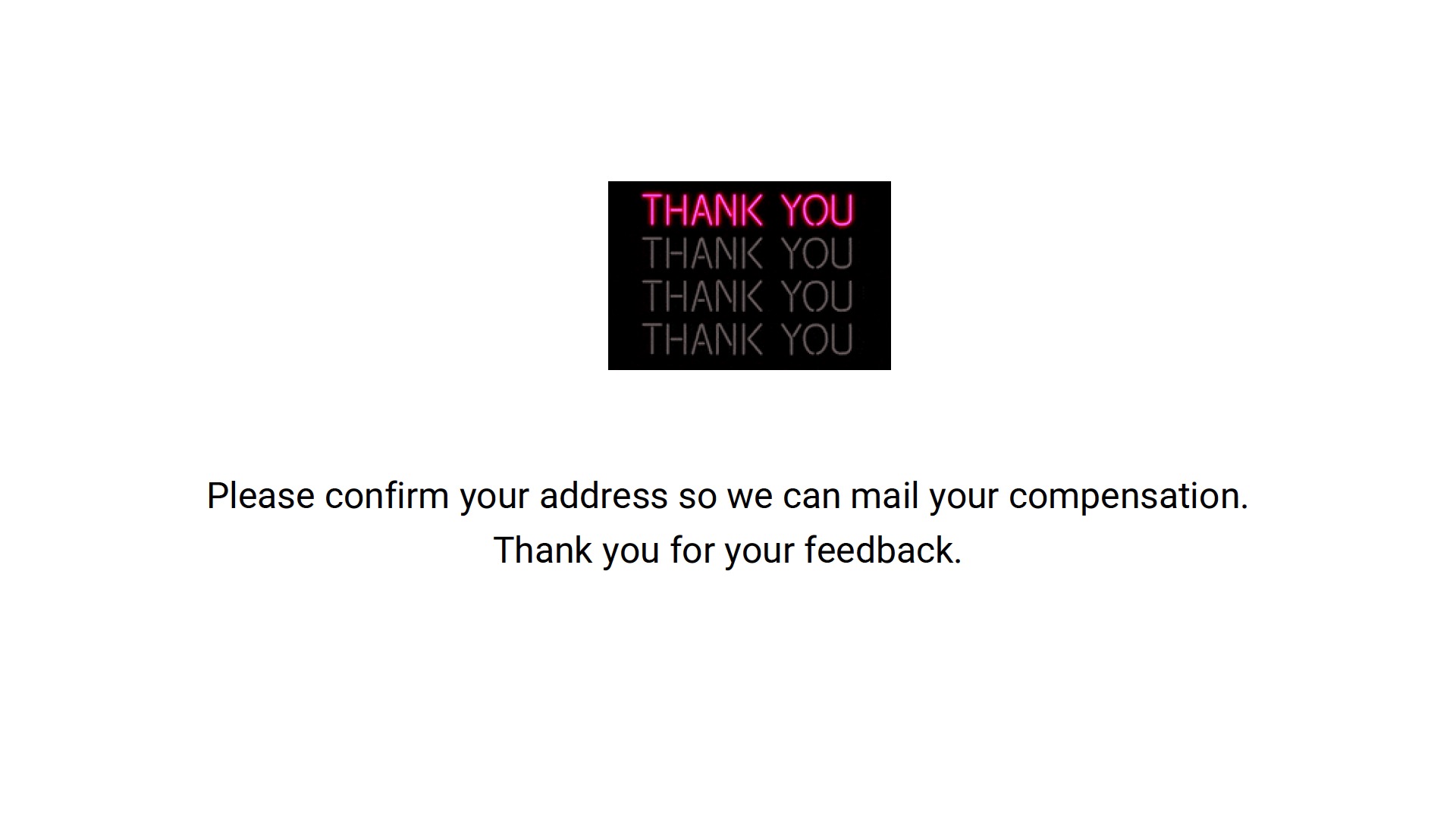

Supplement: Multimedia Appendix 2 [file formative_v10i1e88768_app2.docx]
