# Supplementary material for: Development of a Culturally Adapted Smartphone App (IndigeQuit) Designed to Help American Indian and Alaska Native People Quit Commercial Cigarettes: User-Centered Mixed Methods Study
Source: JMIR Form Res. 2026 Mar 24;10:e88768. doi: 10.2196/88768 (PMC13058535; doi:10.2196/88768)
Supplement: Multimedia Appendix 3 [file formative_v10i1e88768_app3.pdf]

Fred Hutch Cancer Center

Welcome!

Before we begin

- Introduction
- May we record this session?

What to expect

What to expect during this interview

- Learning about your experiences with IndigeQuit
- Reviewing specific features
- What worked best and what could be improved
- Overall impressions and ideas

Feedback on the IndigeQuit app

App Experience

What was using IndigeQuit like for you?

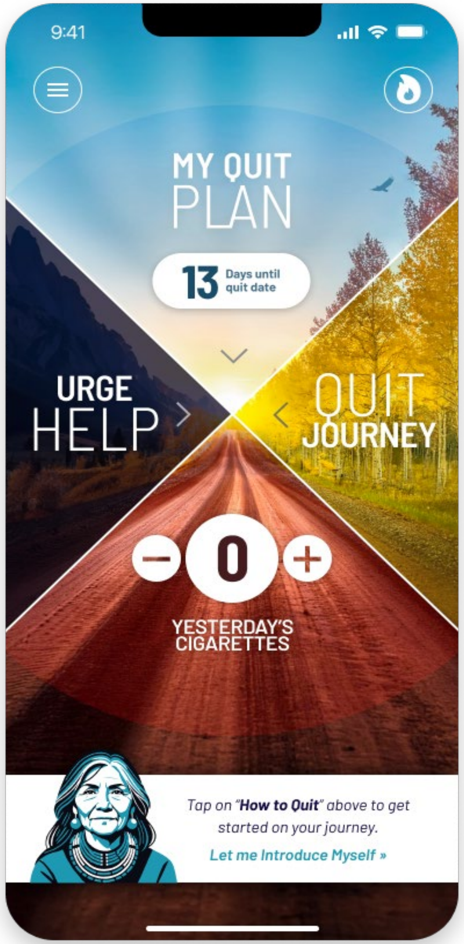

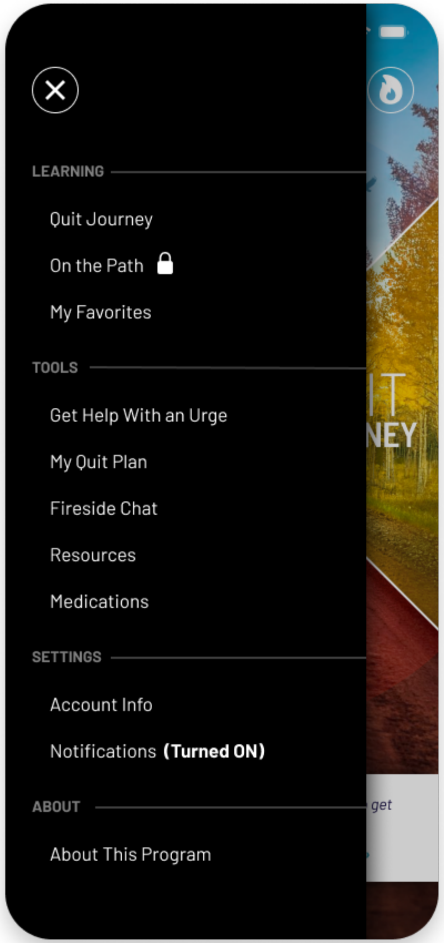

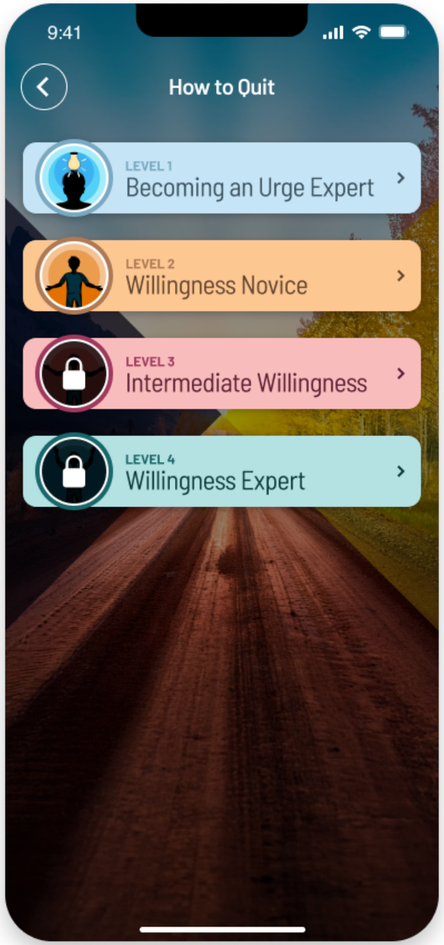

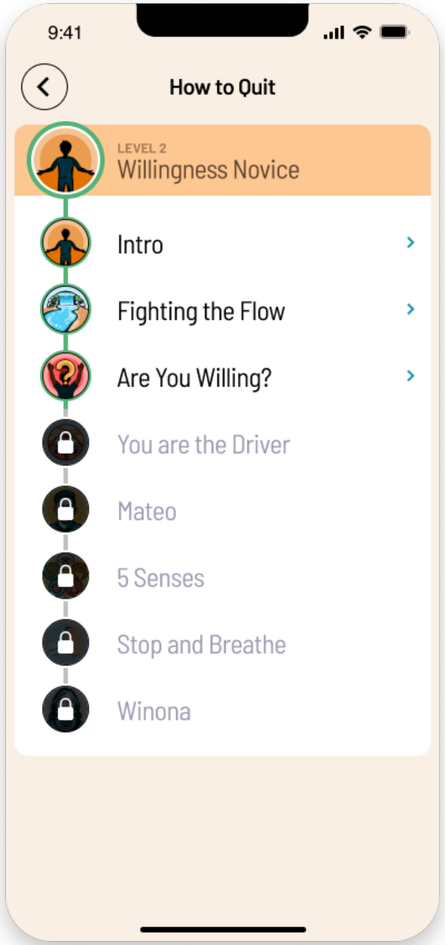

Specific Features

How do you feel about the:

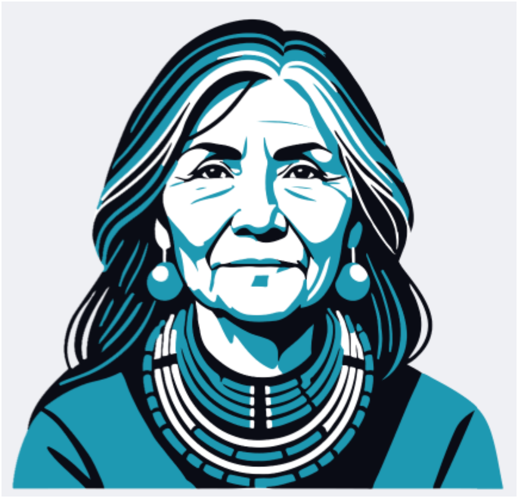

- Quit Guide Winona?
  - How did you feel about how she looks and sounds?
  - What did you think about her stories from her life?

- Push notifications?
  - Were they helpful?
  - How much did you feel like they were made for you?
  - Is there anything that would make you more likely to open the app when you see them?

Specific Features

How do you feel about the:

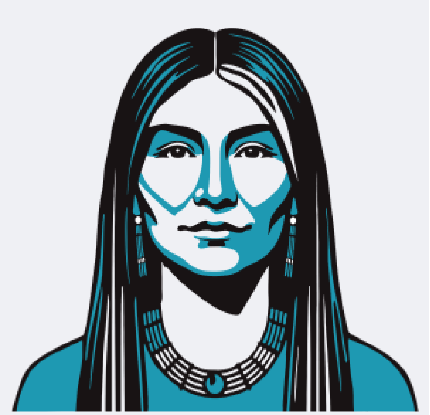

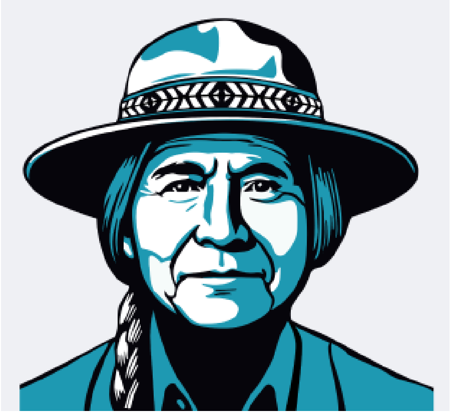

- Stories from the other characters in the app, like Josephine or Sterling?
  - Would anything make the stories more interesting or helpful?

- Urge Help?
  - Would anything make the Urge tips more useful?

- FAQ, especially the section on Ceremonial vs Commercial Tobacco?
  - Was the information helpful? Is there anything else you'd like to see in the FAQ?

Specific Features

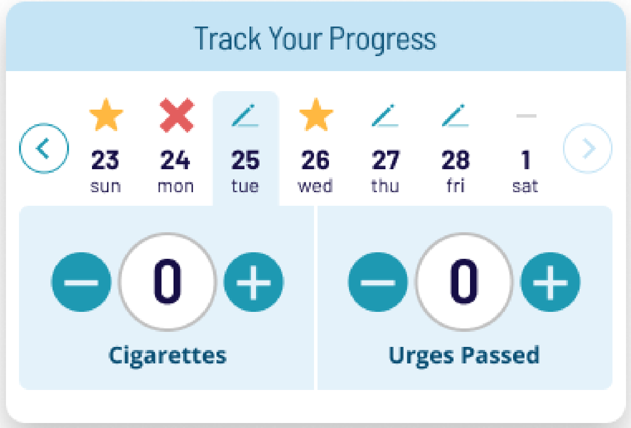

How do you feel about the:

- Calendar tracking (recording cigarettes smoked or smokefree days)?
  - Is there anything that would make tracking better?
  - How was the experience of tracking urges?

- Dropping Anchor tool?
  - Was there anything that should be added or changed to make this tool more helpful?

Specific Features

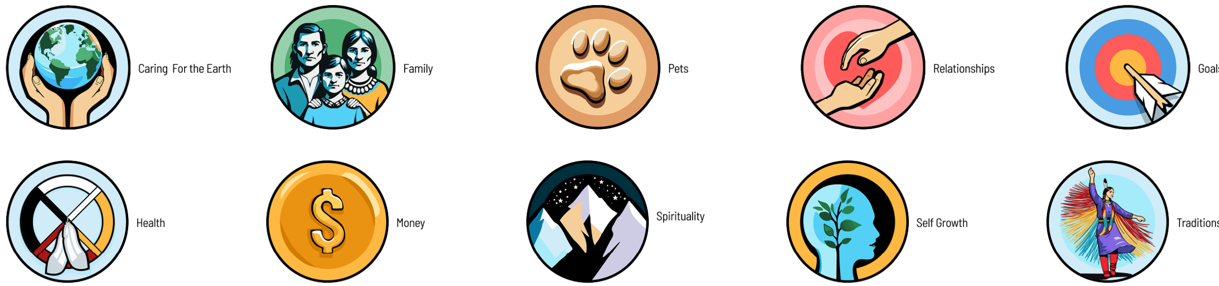

How do you feel about the:

- Motivation for quitting icons?
    - Were the current options helpful?
    - What motivation for quitting is missing?

- Fireside chat?
    - Did you ask any questions?
    - What would make you want to post or why wouldn't you want to post?
    - Was there any other information in the chat that you wanted to see?

Final questions

What parts of the IndigeQuit program seem most helpful quitting smoking?

Notifications	Stories from Winona & other characters	Tools	Tracking urges & cigarettes smoked	Fireside Chat

Audio Exercises	FAQ and medication information	Urge Help	Journey stats/My Quit Plan	Motivations icons

What part of the IndigeQuit program seems least valuable for quitting smoking?

Notifications	Stories from Winona & former smokers	Tools	Tracking urges & cigarettes smoked	Fireside Chat
Audio Exercises	FAQ and medication information	Urge Help	Journey stats/My Quit Plan	Motivations icons

Overall impressions and final thoughts

- What did you like best about IndigeQuit?
- What in IndigeQuit could be improved?
- Any last thoughts or suggestions to share?

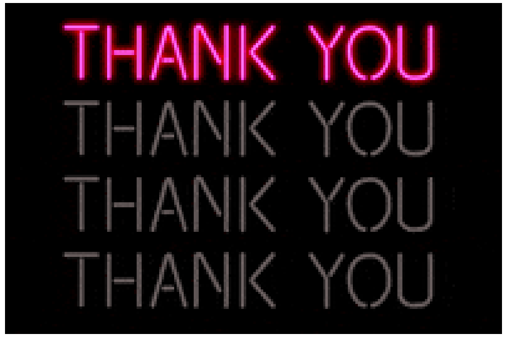

Please confirm your address so we can mail your compensation.

Thank you for your feedback.
